# RNA‐seq analysis of ageing human retinal pigment epithelium: Unexpected up‐regulation of visual cycle gene transcription

**DOI:** 10.1111/jcmm.16569

**Published:** 2021-05-01

**Authors:** Joe M. Butler, Wasu Supharattanasitthi, Yit C. Yang, Luminita Paraoan

**Affiliations:** ^1^ Department of Eye and Vision Science Institute of Life Course and Medical Sciences University of Liverpool Liverpool UK; ^2^ Department of Ophthalmology Wolverhampton Eye Infirmary New Cross Hospital Wolverhampton UK; ^3^ Present address: Center for Experimental and Molecular Medicine (CEMM) Amsterdam UMC Amsterdam Netherlands; ^4^ Present address: Department of Physiology Faculty of Pharmacy Mahidol University Bangkok Thailand

**Keywords:** A2E, ageing, macular degeneration, retinal pigment epithelium, retinol metabolism, RNA‐seq, visual cycle

## Abstract

Ageing presents adverse effects on the retina and is the primary risk factor for age‐related macular degeneration (AMD). We report the first RNA‐seq analysis of age‐related transcriptional changes in the human retinal pigment epithelium (RPE), the primary site of AMD pathogenesis. Whole transcriptome sequencing of RPE from human donors ranging in age from 31 to 93 reveals that ageing is associated with increasing transcription of main RPE‐associated visual cycle genes (including *LRAT*, *RPE65*, *RDH5, RDH10*, *RDH11*; pathway enrichment BH‐adjusted *P* = 4.6 × 10^−6^). This positive correlation is replicated in an independent set of 28 donors and a microarray dataset of 50 donors previously published. *LRAT* expression is positively regulated by retinoid by‐products of the visual cycle (A2E and all‐*trans*‐retinal) involving modulation by retinoic acid receptor alpha transcription factor. The results substantiate a novel age‐related positive feedback mechanism between accumulation of retinoid by‐products in the RPE and the up‐regulation of visual cycle genes.

## 1 INTRODUCTION

Age is a key risk factor for a number of retinal diseases including age‐related macular degeneration (AMD), the leading cause of blindness in higher income countries with ageing populations. One of the critical tissues involved in the pathogenesis of AMD is the retinal pigment epithelium (RPE), a monolayer of pigmented cells located between the vasculature of the choriocapillaris and the neurosensory retina. This unique environment presents the RPE with a number of important functions to support and maintain the visual processing of the retina, including transepithelial transport, absorption of light that has passed through the neurosensory retina, phagocytosis of photoreceptor outer segments and a fundamental role in maintaining the retinoid cycle or visual cycle.^1^ Age‐related changes in the RPE observed through microscopy include the accumulation of more numerous lysosomes, more remnants of phagosomes and morphological changes to mitochondria. However, the most characteristic physical sign of retinal ageing is the accumulation of lipoprotein‐rich sub‐RPE deposits also known as lipofuscin.^2^, ^3^

Lipofuscin accumulation has been implicated in the pathogenesis of atrophic degeneration of the retina, such as dry age‐related macular degeneration (AMD) and Stargardt disease.^4^, ^5^ Key mechanistic insights into the accumulation of lipofuscin were gleaned from observations that its accumulation is dependent on dietary vitamin A^6^ and on a functioning visual cycle.^7^ This latter study showed that in mice with an impaired visual cycle, lacking both copies of the *RPE65* gene, accumulation of lipofuscin in the RPE was abolished. Since then further evidence linking the visual cycle with lipofuscin accumulation and retinal degeneration has been mounting. Impaired clearance of all‐*trans*‐retinal (atRAL), a product of the photoisomerization stage of the visual cycle, has been shown to lead to lipofuscin deposits and retinal degeneration in mice.^8^, ^9^, ^10^, ^11^ If atRAL is not reduced back to retinol, it can inadvertently react to form condensation products such as di‐retinoid‐pyridinium‐ethanolamine (A2E) and all‐*trans*‐retinal dimer. A2E is the product of a condensation reaction between two molecules of atRAL and one molecule of the common phospholipid, phospatidylethanolamine (PE).^12^ The discovery that A2E is a constituent of lipofuscin^13^ provided further evidence linking the visual cycle with lipofuscin accumulation. The accumulation of A2E is detrimental to RPE cells, due to its amphiphilic structure and its photo‐reactivity with blue light.^14^ Photo degradation of A2E generates dicarbonyls (glyoxal and methylglyoxal), which are known to modify proteins by forming advanced glycation end products (AGEs).^15^ In turn, AGEs are well documented to be linked with ageing and a number of age‐related pathologies including AMD.^16^

Quantitative analyses of gene expression changes are essential in furthering our understanding of the mechanisms underlying ageing and age‐related diseases. With respect to the retina, microarray studies have been applied to characterize human RPE transcriptome ^17^, ^18^, ^19^ and more recently both bulk RNA‐seq and single‐cell RNA‐seq have been used to study the neuroretina and RPE,^20^, ^21^ with the latter studies having a particular emphasis on AMD. The current study, to our knowledge, is the first to use RNA‐seq analysis to exclusively investigate the molecular changes underlying the ageing process in the human RPE.

## 2 MATERIALS AND METHODS

### 2.1 Human donor eyes

RPE tissue was dissected from 41 human eyes donated for corneal transplantation and/or medical research through the Manchester Eye Bank, Manchester Royal Eye Hospital, UK, with consent by donors’ next of kin and in accordance with the tenets of the Declaration of Helsinki. Tissue collection and use were carried out under National Research Ethics Service approval obtained through Liverpool Research Ethics Committee and performed in accordance with HTA code of practice regarding utilization of human tissue. Samples from 13 donors ranging in age from 31 to 93 years were used for transcriptome profiling by RNA‐seq and a further 28 samples from donors ranging in age from 45 to 88 years were used for validation by qPCR. Donors with recorded retinal pathology or with over 48 hours between death and RNA isolation were excluded.

### 2.2 Microdissection of RPE/choroid, RPE isolation and RNA extraction

Following enucleation of the globes and removal of the anterior segment as part of the Corneal Transplant Service, the vitreous was removed and the posterior segment was rinsed in phosphate‐buffered saline. The neural retina was peeled away with jeweller's forceps, and the RPE/choroid complex was teased from the sclera with the aid of a binocular dissecting microscope. The RPE‐choroid was then subjected to a modified version of the Simultaneous RPE Isolation and RNA Stabilization (SRIRS) protocol^22^ with appropriate scaling up for human eyes involving placing the dissected RPE‐choroid from one eye in 2 mL RNAprotect Cell Reagent (Qiagen, West Sussex, UK). The solution was agitated for 15 minutes at room temperature allowing the RPE cells to dissociate from the choroid. RNA was extracted using RNeasy Plus Mini Kit (Qiagen, West Sussex, UK). Purity, concentration and quality of RNA preparations were determined using a Nanodrop ND‐100 spectrophotometer (Labtech, Uckfield, East Sussex, UK) and a Bioanalyzer 2100 (Agilent Technologies, Palo Alto, CA, USA), as well as by non‐denaturing electrophoresis. All samples submitted for RNA‐seq had A_260_/A_280_ ratios of the total RNA >2, the ratio of 28S/18S ribosomal RNA bands >1.8 and a RIN value >4. RPE purity was determined using the downstream RNA‐seq analysis; if photoreceptor genes *RHO, SAG, GNAT1, GNB1, GNGT1, PDE6A, PDE6B, PDE6G, ROM1, PRPH2, RCVRN, CNGA1, CNGB1 and GUCA1A* ^23^ cumulatively contributed to more than 1% of the total transcriptome then the sample was excluded.

### 2.3 RNA‐seq analysis: cDNA library preparation and sequencing

Total RNA was processed by the Centre for Genomic Research, University of Liverpool, for library preparation and sequencing using the Illumina HiSeq 2000 platform. Ribosomal RNA (rRNA) was depleted from total RNA using the Ribo‐Zero rRNA Removal kit (Epicentre, Madison, WI, USA). cDNA libraries were prepared with the ScriptSeq v2 RNA‐Seq library preparation kit (Epicentre) by using 50 ng rRNA‐depleted RNA as starting material. One lane of the Illumina HiSeq 2000 was used to sequence eight samples using paired‐end sequencing (2 × 100 bp). The raw sequence data and processed counts data were deposited in the GEO repository (https://www.ncbi.nlm.nih.gov/geo/) under accession number GSE159435.

### 2.4 Mapping and gene level analysis

The raw fastq files were trimmed for the presence of Illumina adapter sequences using Cutadapt version 1.2.1.^24^ This trimmed the 3' end of any reads which match the adapter sequence for 3bp or more. The reads were further trimmed using Sickle version 1.200 (window quality score > 20). Reads shorter than 10bp after trimming were removed. Quality control checks and measures were performed using FastQC.^25^ Mapping of the paired reads to the human reference genome (hg19) was performed using TopHat2 version 1.3.0 KIM201, using default settings. Read counts were measured with HTSeq,^26^ using the genome annotation by Ensembl (GRCh37.72) (n = 62,893).

Gene level analysis was carried out using DESeq2,^27^ which automatically filters out weakly expressed genes (to improve performance by reducing multiple testing). There remained 20,154 genes after filtering. As we were interested in the effect of age on gene expression, DESeq2 was implemented to perform a linear regression between age and the read count of each gene. Age was the variable of interest; however, we also included the additional covariates of gender and sequencing batch (1 or 2), making this a multi‐factor design. As our independent variable (age) is continuous rather than dichotomous, we find it more appropriate to define a significantly correlated gene (SCG) rather than the conventional differentially expressed gene. An SCG is any gene with an expression level significantly correlated (positively or negatively) with age. Specifically, we define an SCG as any gene with an expression level correlated with age such that its Benjamini‐Hochberg (BH) adjusted *P*‐value < .05 and its |β_age_| > 0.025. Applying the standard adjusted *P*‐value cut‐off of .05 filters out 98% of all genes, then the β_age_ cut‐off of 0.025 (which excludes genes with a small gradient) filters out less than 1% of the total number of genes.

### 2.5 Validation of gene expression data by real‐time quantitative PCR (qPCR)

To remove any remaining genomic DNA after RNA extraction, the independent set of 28 human donor samples were treated with DNA‐free DNA Removal Kit (Invitrogen, UK). Complementary DNA was then synthesised with First Strand cDNA Synthesis Kit (Life Technologies/Thermo Fisher Scientific, UK), following the manufacturer's standard protocols. qPCR was performed with technical triplicates using a modified protocol described previously^28^ with MESA BLUE qPCR Mastermix Plus Kit for SYBR assay (Low ROX; Eurogentec, Southampton, UK) and a Stratagene MX3005P^®^ qPCR System (Stratagene, La Jolla, CA, USA). The primer sets (Table S1) were custom synthesized (Eurogentec, Southampton, UK). The melt curve analysis and agarose gel electrophoresis were used to confirm the specificity of amplification reactions. The comparative expression value of the gene of interest was calculated using the efficiency‐corrected ddCt method, with normalization against three house‐keeping genes (*GAPDH*, *RPL5* and *ACTB*) for the in situ data and four house‐keeping genes (*GAPDH*, *RPL5*, *ACTB* and *TUBB*) for the hfRPE in vitro studies. All house‐keeping genes were selected and combined into a single standard using BestKeeper version1.^29^

### 2.6 Gene set and pathway enrichment analyses

To test whether a KEGG pathway was enriched with SCGs we used the hypergeometric test, applying Benjamini‐Hochberg adjustment to cater for multiple testing. SCGs were rendered onto the relevant KEGG pathway map using the Pathview package in R/Bioconductor.^30^ Gene set enrichment analysis (GSEA)^31^ was applied to a single pathway of the Reactome database.^32^

### 2.7 Validation using publicly available microarray gene expression data

Gene Expression Omnibus dataset (GSE29801)^33^ was downloaded, and only patients with no ocular disease were selected. To combine both macular and periphery data, we implemented a mixed model for each gene where retinal position and age are the fixed effect predictors and the patient ID is the random effect predictor.

### 2.8 In vitro measurement of effect of A2E, atRAL and Am580 on *LRAT* expression

Human foetal RPE (hfRPE) cells were grown to confluence in hfRPE media (15% FBS) and then maintained in 5% FBS as previously described.^34^ Stock solutions (10 mmol/L) of Am580 (Sigma‐Aldrich, Merck Life Science UK Limited, Gillingham, UK), all‐*trans*‐retinal (Sigma‐Aldrich) and A2E (gift from T. Michael Redmond, synthesised as previously described^35^) were prepared in DMSO vehicle. A2E and Am580 were diluted to a final concentration of 25 μmol/L, and all‐*trans*‐retinal to 5 μmol/L, in hfRPE media containing 5% FBS. Cells were treated with a single dose of A2E, Am580, all‐*trans*‐retinal or vehicle control. All conditions were performed in triplicate wells. After the assigned time, RNA was extracted using RNAprotect Cell Reagent (Qiagen, West Sussex, UK) followed by standard RNeasy Plus Mini Kit (Qiagen, West Sussex, UK). All manipulations involving A2E, all‐*trans*‐retinal, Am580 and vehicle were conducted under dim (20‐40 lux) red (638 nm) safelight.

## 3 RESULTS

### 3.1 Overview of RNA sequencing data

**FIGURE 1:**
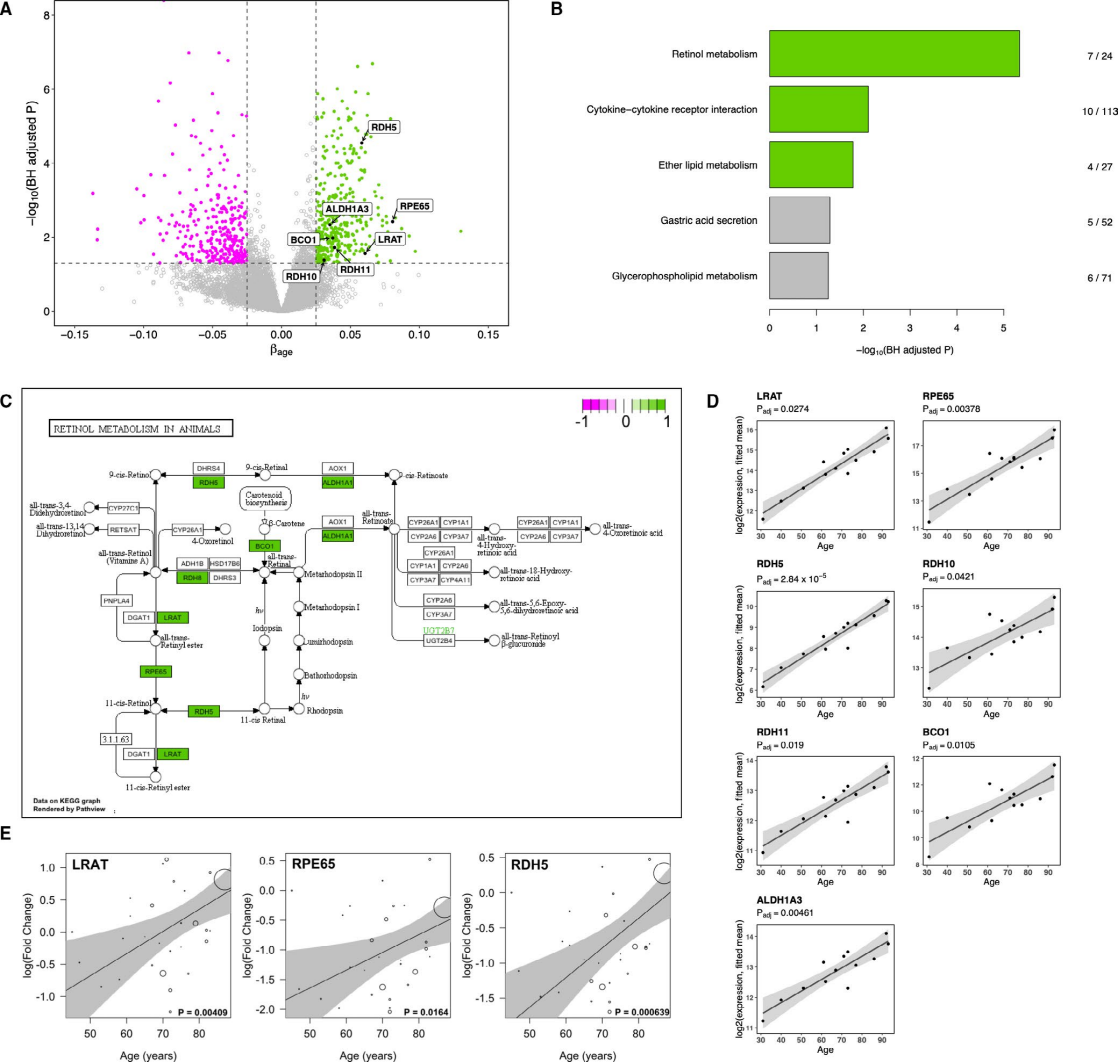
RNA‐seq of RPE reveals visual cycle genes are transcriptionally up‐regulated with increasing human age. A, Volcano plot showing up‐regulated SCGs in green and down‐regulated in magenta. Genes in the retinol metabolism KEGG pathway are labelled. B, Pathway enrichment analysis of 341 KEGG pathways identifies retinol metabolism (map00830) to be the most significantly enriched with positively correlated SCGs. Pathways with a BH‐adjusted *P* < .05 are highlighted in green. Fraction represents the number of SCGs in the pathway over the total number of genes in the pathway. C, KEGG pathway map of retinol metabolism (map00830), with positive SCGs highlighted in green. D, Scatter plots of the expression of 7 retinol metabolism genes from human donors (n = 13) ranging in age from 31 to 93 years. Regression line (black) with 95% confidence interval (grey shading). E, qPCR validation of the positive correlation between age and transcription level of the core visual cycle genes (LRAT, RPE65 and RDH5). Validation carried out in a separate set of 28 human donors aged 45 to 87 years. Regression line (black) with 95% confidence interval (grey shading); regression was weighted by the RNA integrity score of each sample shown by size of circle

The set of RPE expressed genes on which all following analyses were carried out comprised of 20,154 genes obtained through RNA‐seq analysis of human donor RPE cells, followed by filtering out weakly expressed genes by DESeq2. Out of this set, we defined a significantly correlated gene (SCG) to be one in which its expression level is correlated with age such that its Benjamini‐Hochberg (BH) adjusted *P*‐value < .05 and |β_age_| > 0.025, where β_age_ represents the estimated change in gene expression per year of life. Using this definition, we determined a total of 822 SCGs (Table S2). Of these, the majority (n = 659) were protein coding. The non‐coding SCGs included 93 long non‐coding RNAs (lncRNAs), 35 pseudogenes and six small nuclear and small nucleolar RNAs (snRNAs and snoRNAs). The fact that the expression of 93 lncRNAs is significantly correlated with age corresponds with mounting evidence showing the importance of these RNAs in ageing.^36^ Of the total 822 SCGs, 447 were up‐regulated with increasing age (positively correlated) and 345 were down‐regulated with increasing age (negatively correlated) (Figure 1A). These results demonstrate that the transcriptome of the human RPE exhibits significant age‐related transcriptional changes.

### 3.2 KEGG pathway enrichment analysis reveals multiple age‐related pathways and identifies retinol metabolism to be the most significantly up‐regulated pathway

We next performed pathway enrichment analysis using the 341 human KEGG pathways, separately for positive SCGS and negative SCGs. For the positive SCGs, we found that the most significantly enriched pathway is the *retinol metabolism* (hsa00830) pathway, in which seven out of the 24 pathway genes are positive SCGs (BH‐adjusted *P* = 2.58 × 10^−5^) (Figure 1B,C). The seven SCGs in this pathway are lecithin retinol acyltransferase (*LRAT*), retinoid isomerase (*RPE65*), three retinol dehydrogenases (*RDH5, RDH10* and *RDH11*), beta‐carotene oxygenase 1 (*BCO1*) and a retinaldehyde dehydrogenase 3 (*ALDH1A3*) (Table 1). For each of these seven genes, the positive correlation between its expression and age of the 13 donors is shown in Figure 1D. The significant positive correlation with age of the expression of all these genes indicated that the retinoid cycle in the RPE is transcriptionally up‐regulated with increasing human age. We further investigated the expression of three of these genes (*LRAT*, *RPE65* and *RDH5*) by qPCR in an independent set of 28 human donors ranging in age from 41 to 90 years. For all three genes, a significant positive correlation between donor age and RPE transcription level was determined (Figure 1E).

**TABLE 1:** KEGG pathways most significantly enriched with up‐regulated SCGs

Pathway	KEGG ID	*P*	BH.adj.*P*	Genes in pathway	SCGs in pathway	Gene symbols of SCGs in pathway
Retinol metabolism	hsa00830	1.62 × 10^−7^	4.6 × 10^−6^	24	7	*ALDH1A3, BCO1, LRAT, RDH10, RDH11, RDH5, RPE65*
Cytokine‐cytokine receptor interaction	hsa04060	.000298	.00783	113	10	*ACVR1C, BMP4, BMP6, BMP7, BMP8B, CXCR2, GDF11, IL16, IL17RB, TNFRSF10C*
Ether lipid metabolism	hsa00565	.00078	.0166	27	4	*ENPP2, PLA2G5, PLA2G7, SELENOI*
Gastric acid secretion	hsa04971	.00303	.0517	52	5	*CAMK2B, HRH2, PRKCB, SLC26A7, SLC4A2*
Glycerophospholipid metabolism	hsa00564	.00346	.0561	71	6	*GPAM, GPD1, PCYT1B, PLA1A, PLA2G5, SELENOI*
Cysteine and methionine metabolism	hsa00270	.00424	.0628	39	4	*BCAT1, CDO1, CTH, GOT2*
Bile secretion	hsa04976	.00527	.0691	41	4	*SLC22A8, SLC4A2, SLC4A5, SLCO1A2*
Mineral absorption	hsa04978	.00527	.0691	41	4	*ATP2B2, CLCN2, TRPM6, TRPM7*

The second most significantly up‐regulated pathway is *cytokine‐cytokine receptor interaction* (hsa04060; BH‐adjusted *P* = .0018) (Table 1). The genes up‐regulated in this pathway include bone morphogenetic proteins (*BMP4, BMP6, BMP7, BMP8B*) that are part of the transforming growth factor‐beta superfamily, interleukins and chemokine receptors (IL16, IL17RB and *CXCR2*). This finding is consistent with the increasing body of evidence of increased inflammation in the ageing RPE,^37^ and increased expression of the chemokine signalling in AMD.^38^ The third most significantly up‐regulated pathway is *ether lipid metabolism* (hsa00565). One of the most important classes of ether lipids is plasmalogens, and their function in the RPE may be involved in protection against oxidative stress; plasmalogen deficiency is associated with early onset retinal abnormalities and/or retinopathy.^39^

Many pathways were found to be significantly down‐regulated with age (Figure S1A; Table 2), the most significant one being *protein digestion and absorption* (Figure S1); this pathway is annotated in relation to nutritional homeostasis and as such this finding may reflect some similarities between the RPE and the intestinal epithelial cells such as phagolysosome function. Interestingly, five of the eight significantly down‐regulated genes in this pathway are collagens (Table 2) and two in particular have been found to have relevance for ocular ageing. Collagen type IV alpha 6 (*COL4A6*) is a major structural component of basement membranes and is found in most ocular basement membranes.^40^ Collagen type II alpha 1 (*COL2A1*) is a fibrillar collagen found in the vitreous humour and mutations in this gene are associated with Stickler syndrome in which patients suffer from ocular pathologies such as vitreous degeneration, high myopia, retinal detachment and cataracts.

**TABLE 2:** KEGG pathways most significantly enriched with down‐regulated SCGs

Pathway	KEGG ID	*P*	BH.adj.*P*	Genes in pathway	SCGs in pathway	Gene symbols of SCGs in pathway
Protein digestion and absorption	hsa04974	5.33 × 10^−7^	1.51 × 10^−5^	61	8	*ATP1A2, COL25A1, COL2A1, COL4A6, COL6A3, COL9A3, SLC8A1, SLC8A3*
Cell adhesion molecules	hsa04514	6.77 × 10^−7^	1.78x × 10^−5^	101	10	*CADM1, CD274, CDH2, CNTNAP2, LRRC4C, NCAM1, NLGN1, NLGN4X, NRXN1, NRXN3*
Neuroactive ligand‐receptor interaction	hsa04080	1.46 × 10^−6^	3.56 × 10^−5^	109	10	*CHRM4, DRD1, GABBR2, GRIA3, GRIA4, GRID2, GRIK1, GRM1, HTR2A, OXTR*
Calcium signalling pathway	hsa04020	4.95 × 10^−6^	.000113	148	11	*CAMK4, DRD1, GRM1, HTR2A, MYLK4, NTRK3, OXTR, PDE1A, SLC8A1, SLC8A3, TRDN*
Glutamatergic synapse	hsa04724	7.43 × 10^−6^	.000158	83	8	*ADCY5, DLGAP1, GRIA3, GRIA4, GRIK1, GRM1, KCNJ3, SLC38A1*
Morphine addiction	hsa05032	1.05 × 10^−5^	.000208	66	7	*ADCY5, DRD1, GABBR2, KCNJ3, PDE11A, PDE1A, PDE4D*
Amphetamine addiction	hsa05031	1.10 × 10^−5^	.000208	48	6	*ADCY5, CAMK4, DRD1, GRIA3, GRIA4, PPP1R1B*
cAMP signalling pathway	hsa04024	1.67 × 10^−5^	.000299	140	10	*ADCY5, ATP1A2, CAMK4, DRD1, GABBR2, GRIA3, GRIA4, OXTR, PDE4D, PPP1R1B*
Gap junction	hsa04540	2.72 × 10^−5^	.000464	75	7	*ADCY5, DRD1, GRM1, GUCY1A2, HTR2A, PRKG1, TUBB2B*

The next most significantly down‐regulated pathway is *cell adhesion molecules (CAMs)* (hsa04514). This includes genes such as cadherins (*CDH2*), neurexins (*CNTNAP2, NRXN1, NRXN2 and NRXN3*) and other cell adhesion molecules (*CADM1, L1CAM and NCAM1*) (Table 2). Adhesion between RPE cells and the underlying Bruch's membrane is essential for RPE barrier and retinal support functions, and defects in adhesion are suspected to play a role in choroidal neovascularization (CNV) initiation and progression.^41^ Previous research in our laboratory found that advanced glycation end products (AGEs), which are known to accumulate on the Bruch's membrane with increasing age,^16^ reduce the transepithelial resistance of the RPE.^42^ Thus, taken together, we propose that with increasing age, the accumulated AGEs on the Bruch's membrane lead to decreased expression of CAMs by the RPE, which results in reduced transepithelial resistance. We also note that these molecules may be involved in maintaining attachment of the RPE to the neurosensory retina, and that they are down‐regulated with age may explain the increased risk of rhegmatogenous retinal detachment in the elderly.^43^ It is also intriguing that cell adhesion molecules were found to be remarkably enriched in common protein networks shared between age‐related diseases and longevity‐associated processes.^44^ The fact that these adhesion molecules have been linked to longevity invites future research to explore whether restoring levels of these molecules may ameliorate the effects of ageing in the retina and elsewhere in the body.

Finally, we perform a KEGG pathway enrichment analysis of the full set of 822 SCGs (ie both positively and negatively correlated genes). The most significantly enriched pathway in this analysis is the *Calcium signalling pathway* (hsa04020), which is also one of the pathways previously observed when only considering the down‐regulated genes (Figure S1A). Calcium is needed for a number of regulatory processes in RPE cells such as photoreceptor outer segment phagocytosis, transcellular fluid and ion transport, cell differentiation and the control of gene expression.^45^ Our finding suggests that calcium signalling in the RPE may become dysregulated with increasing age.

### 3.3 Reactome pathway analysis confirms the visual cycle gene transcription up‐regulation with age and identifies further significantly associated genes

**FIGURE 2:**
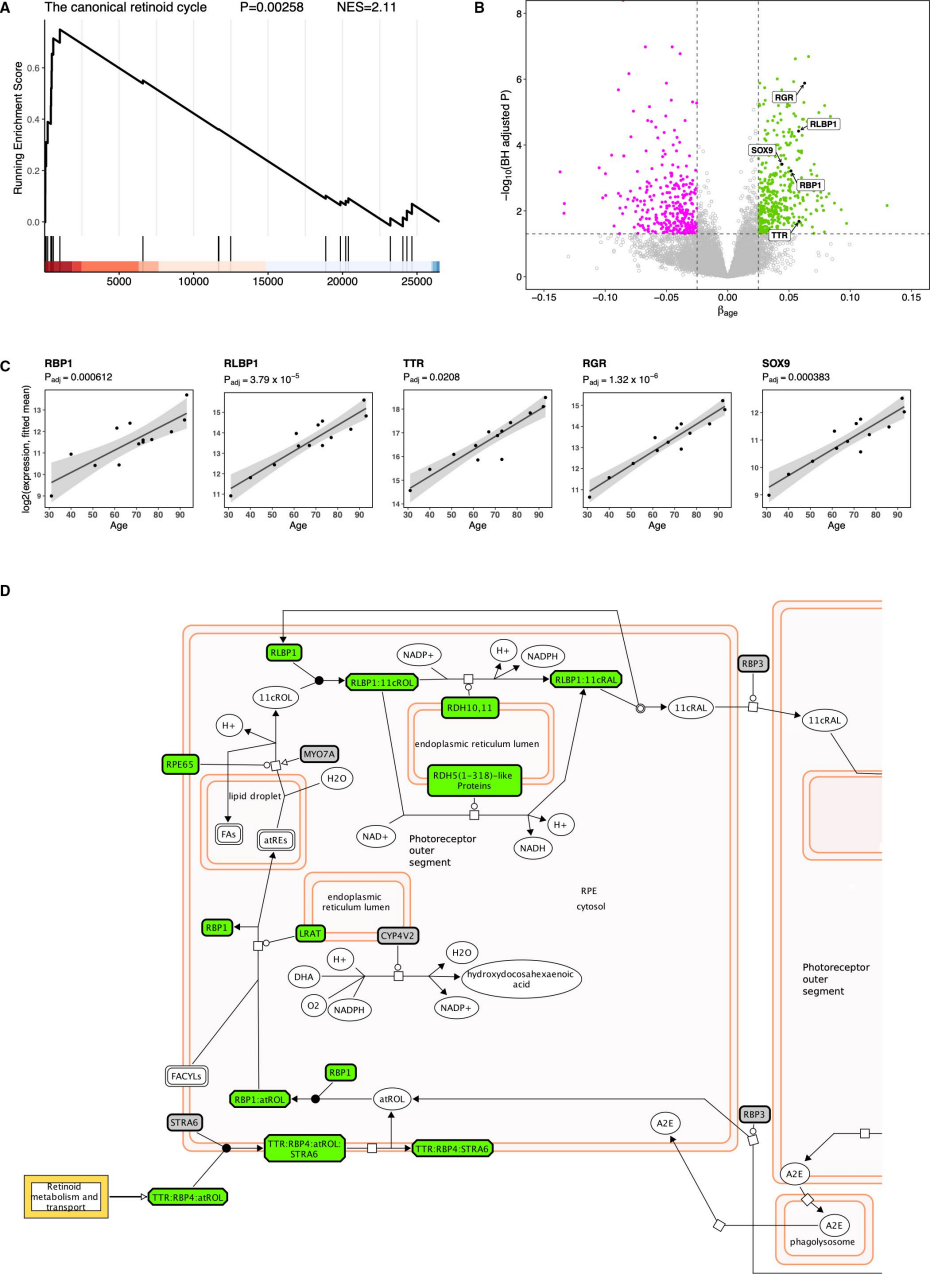
Reactome pathway analysis identifies a further set of visual cycle‐related genes that have increased expression with increasing age. A, GSEA enrichment plot for the canonical retinoid cycle pathway (R‐HSA‐2453902) from the Reactome database. B, Volcano plot showing expression levels of additional visual cycle genes identified from the Reactome pathway (RLBP1, RBP1 and TTR), as well as two other visual cycle genes previously documented (SOX9 and RGR). C, Scatter plots of the expression levels of five additional retinol metabolism genes, from human donors (n = 13) ranging in age from 31 to 93 years. Regression line (black) with 95% confidence interval (grey shading). D, Visual cycle diagram adapted from Reactome pathway browser.^74^ Significantly positively correlated genes, and complexes which include a SCG, are highlighted in green. Non‐significant genes are in grey and other compounds which are not proteins are shown in white

From herein, we focus the analysis on the *retinol metabolism* (hsa00830) KEGG pathway due to both its novel link with ageing and its highest level of significance. Having identified this pathway to be the most significantly up‐regulated KEGG pathway, we sought to test this finding using the same data in a different pathway database, namely the Reactome database. We identified the pathway most closely resembling the KEGG *retinol metabolism* to be the pathway named *The canonical retinoid cycle in rods (twilight vision)* [R‐HSA‐2453902] (despite the mention of rods in its name, the description of the pathway states that this also takes place in the RPE). Here, we applied the Gene Set Enrichment Analysis (GSEA) approach and confirmed that this pathway is indeed significantly enriched (*P* = .00258) with a positive Normalized Enrichment Score (NES), confirming this pathway is up‐regulated with age (Figure 2A). This analysis also revealed three further positive SCGs related to the retinoid cycle; these are retinol‐binding protein (*RBP1*), retinaldehyde‐binding protein 1 (*RLBP1*; also known as *CRALBP*) and transthyretin (*TTR*). For all three of these genes, the positive correlation between expression and age is highly significant, with a BH‐adjusted *P* < .01 (Figure 2B‐D). Two more genes related to the retinoid cycle known to be expressed in the RPE, namely RPE‐retinal G protein‐coupled receptor (*RGR*) and transcription factor *SOX9* (Masuda et al 2014) were also found significantly positively correlated with age (Figure 2B,C). In total, the combined analysis found 12 visual cycle‐associated genes transcriptionally up‐regulated with increasing age.

Upon observing the *SOX9* transcription factor is itself transcriptionally up‐regulated with age, we sought to investigate if its target genes were enriched within the 822 age‐related SCGs. Using a transcription factor (TF) enrichment analysis tool called ChIP‐X Enrichment Analysis 3 (ChEA3),^46^ we found the *SOX9* target gene set was ranked 93rd out of all 1632 TFs (ie in the top 6%) using the mean rank across all ChEA3 libraries. When using only the GTEx coexpression library the *SOX9* gene set was ranked 3rd (top 0.2%), and this was highly significant (FDR = 2 × 10^−11^). We also noticed that the target genes of the Retinoic Acid Receptor Related Orphan Receptor B (*RORB)* transcription factor were also highly enriched within our SCGs; *RORB* was ranked 9th when using the mean rank of all libraries and 82nd in the GTEx coexpression library, which was also highly significant (FDR = 4.65 × 10^−5^).

### 3.4 Validation of age‐related increase in visual cycle gene transcription using previously published microarray expression data

**FIGURE 3:**
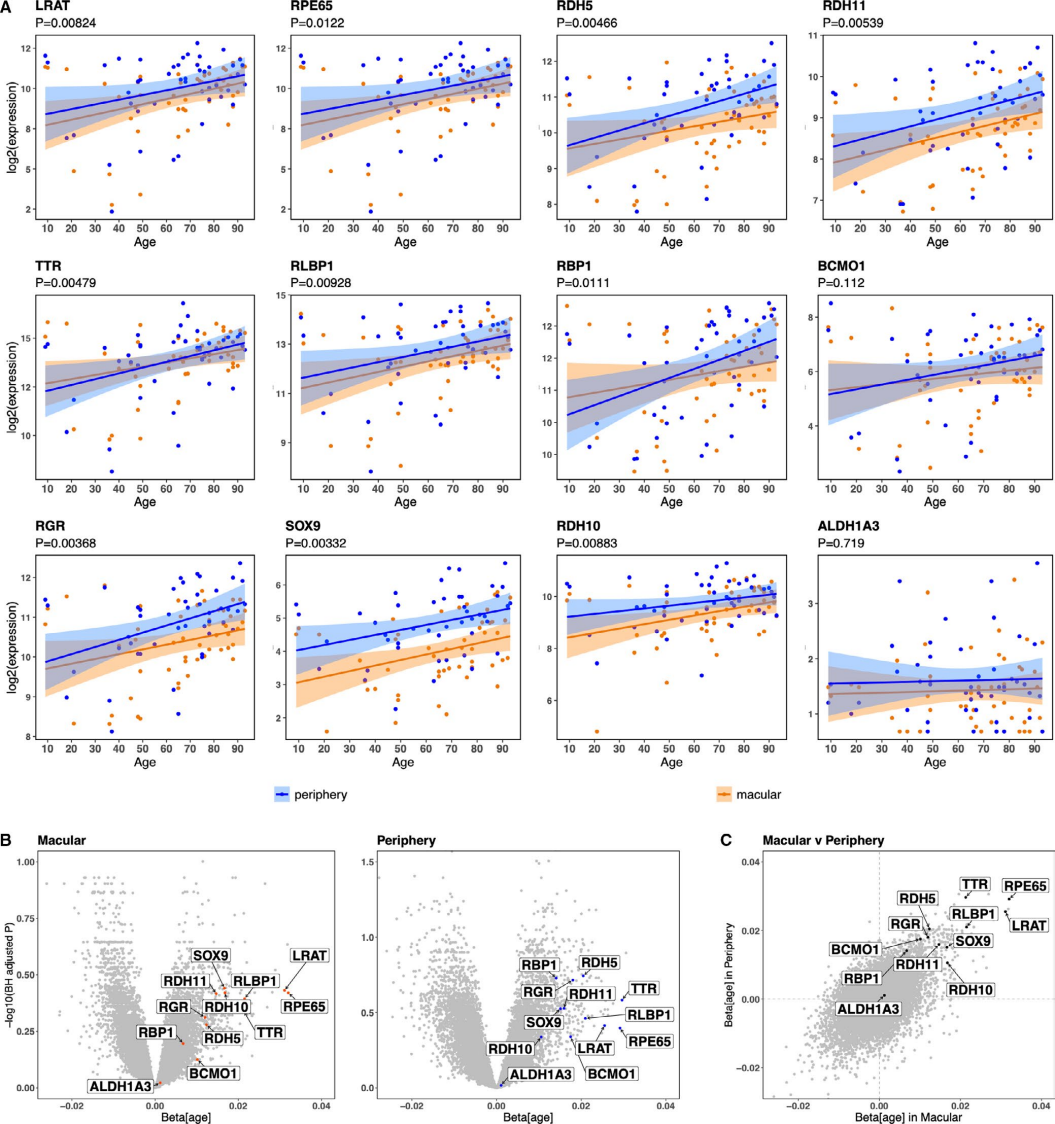
Microarray data from an independent cohort GEO29801^33^ confirm that visual cycle genes exhibit increased expression with increasing age in both macular and periphery of the RPE. A, Scatter plots of the 12 previously identified visual cycle genes from donors with no ocular disease and with an age ranging from 9 to 93 years. Showing data from both the macular (in blue; n = 50) and the periphery (in orange; n = 46). To calculate the overall *P*‐value (H_0_:β_age_ = 0) combining both macular and periphery data, we implemented a mixed model for each gene. B, Volcano plots for macular and periphery RPE gene expression with respect to age. *P* values are adjusted for all genes in the genome using the Benjamini‐Hochberg procedure. C, Showing the relationship of the gradient (β_age_) in the macular versus periphery for the whole RPE transcriptome. Visual cycle genes highlighted in black are all in the upper‐right quadrant, indicative of similar age‐related effect on their transcription in both regions. All data for this figure are selected from the Gene Expression Omnibus dataset GEO29801^33^

Next, we sought to validate our results using publicly available expression data from an independent set of donors. For this purpose, we performed a search of all publicly available RPE transcriptome data and selected the study with the largest range of age of donors with no ocular disease (used as controls in the respective study).^33^ This dataset contains microarray transcriptome data of the RPE from 50 normal human donors from the USA ranging in age from 10 to 93. All 50 donors have a sample from the macula, and 46 have also a sample from the periphery. All the 12 previously identified visual cycle‐related SCGs were found to be positively correlated with age (β > 0) in this independent cohort, and 10 of them were significant (*P* < .05) when combining both macular and periphery data (Figure 3A). All the core visual cycle‐related genes (*RPE65, LRAT* and the three *RDH*s) were validated in this independent cohort; the two genes *BCMO1* and *ALDH1A3* that did not validate are more peripheral in the visual cycle biochemistry. We also assessed macular and periphery data separately and observed all 12 visual cycle‐related genes are up‐regulated with age in the macula alone, and likewise in the periphery alone (Figure 3B). Additionally, when comparing the effect of age in the macula versus the periphery, we detected positive correlation between the respective transcriptional profiles of the visual cycle‐related genes (Figure 3C). These results, as well as validating our primary finding, which was discovered from pooling RPE cells from the entire posterior retina, show that increasing age leads to increased visual cycle gene expression in both the macular and the periphery of the retina.

### 3.5 *LRAT* transcription is positively regulated by A2E and atRAL treatment in hfRPE cells

**FIGURE 4:**
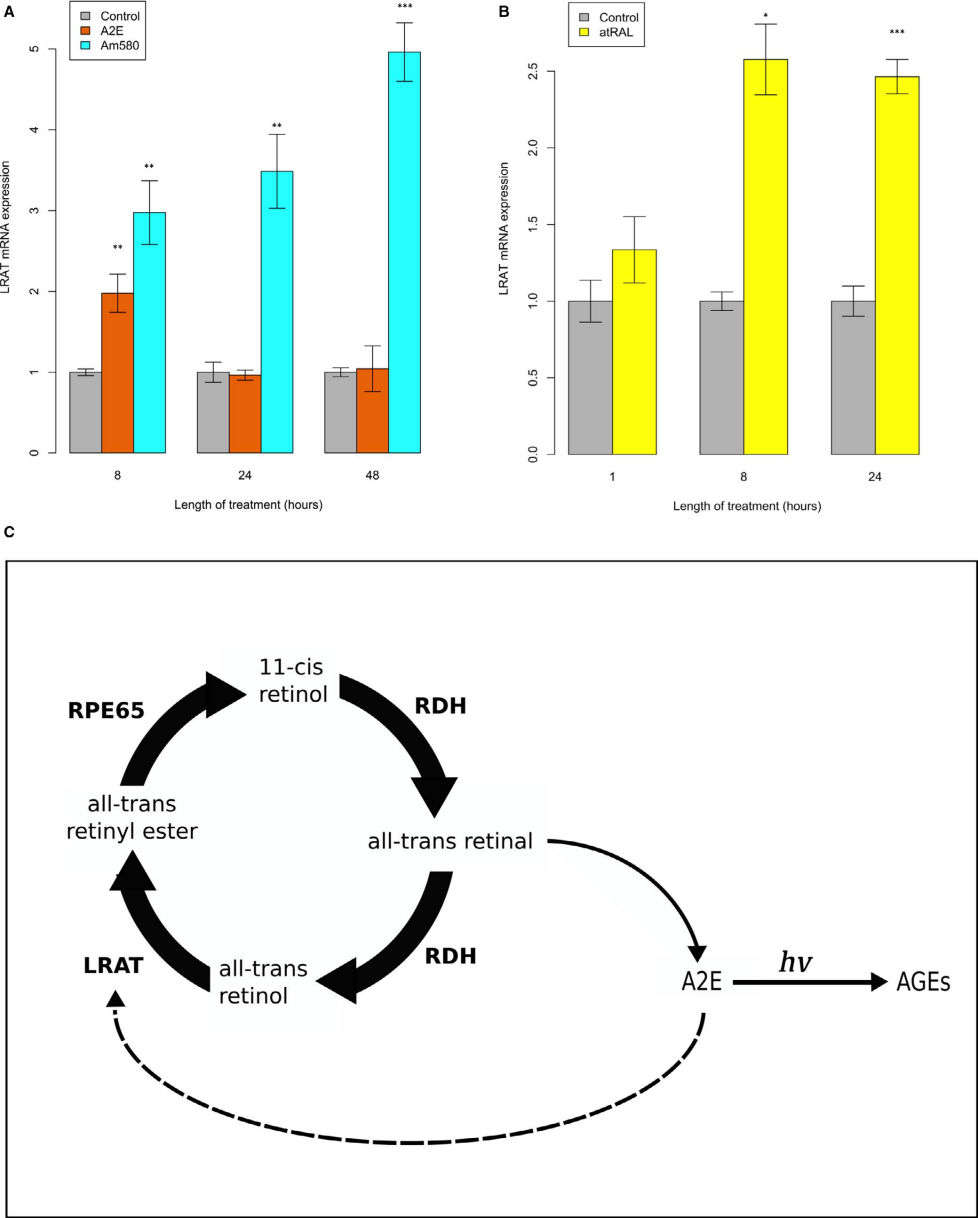
Visual cycle retinoid by‐products positively regulate LRAT transcription in hfRPE cells. A, Two separate hfRPE cell lines were tested for LRAT transcription after treatment with A2E and Am580 for 8, 24 and 48 hours. B, A single hfRPE donor was tested for LRAT transcription after treatment with atRAL for 1, 8 and 24 hours. For all experiments, four house‐keeping genes (ACTB, TUBB, RPL5 and GAPDH) were used as internal controls. All values are calculated relative to the control of the corresponding time‐point. The data shown are mean ± standard error (for each donor, at each time‐point, we performed a minimum of 3 replicates). Asterisks above bars represent significance levels: < 0.05 (*), < 0.01 (**), < 0.001 (***). C, Proposed mechanistic model illustrating the positive feedback effect of A2E upon the visual cycle. Solid broad arrows represent chemical reaction of the visual cycle, catalysed by the visual cycle enzymes. Narrow solid arrows represent non‐enzymatic reaction. Dotted arrow represents positive effect of A2E upon transcription of LRAT

The unexpected result of enhanced RPE expression of visual cycle‐related genes with ageing prompted the investigation of the effect of visual cycle by‐products, known to accumulate in the RPE with progressing age, on the transcription of these genes. We observed that *LRAT* mRNA expression showed a significant increase after 8 hours of A2E treatment (Figure 4A). An even greater increase in *LRAT* expression was observed after 8 hour treatment with Am580, a selective retinoic acid receptor alpha (RARα) agonist (and resistant to rapid catabolism by enzymes of the cytochrome P450 CYP26 family). For Am580, further increases in *LRAT* expression were observed after 24 and 48 hours exposure with a linear trend. These results indicate that *LRAT* transcription is positively regulated in a RARα‐mediated manner. The increased *LRAT* transcription following 8 hour A2E exposure implies that A2E acts through RARα to increase *LRAT* expression. After 24 and 48 hours exposure to A2E, we did not observe a significant increase in *LRAT* expression. As it has previously been shown that a functional RPE lysosome is able to clear A2E,^47^, ^48^ we propose that the observed transcriptional trend in the presence of A2E is explained by the RPE cells’ response to the A2E, which after a number of hours begins to be cleared by the functional hfRPE lysosome. In contrast the synthetic retinoid Am580 is unable to be cleared, even by a functional lysosome. We also determined that *LRAT* expression increased in hfRPE after exposure to all‐*trans*‐retinal (atRAL) (Figure 4B). We also tested the *RPE65* and *RDH5* expression after exposure to these retinoids in this experimental model but did not find evidence of up‐regulation (data not shown), indicating that these two visual cycle genes are under different regulatory control compared with *LRAT*.

## 4 DISCUSSION

The transcriptome analysis performed by this study reveals important ways in which the human RPE changes with age. Most importantly, we discovered that the transcription level of genes involved in the visual cycle are up‐regulated with advancing age. To our knowledge, this is the first time that transcriptional up‐regulation of this pathway has been reported in the ageing human RPE. However, a strikingly similar observation has been reported in the rat where a RNA‐seq transcriptome study of the rat retina found that the genes up‐regulated in senescence accelerated rats ‘*are unexpectedly associated with phototransduction*’.^49^ In our study, we observe age‐related increase in expression of the main visual cycle genes: *LRAT*, *RPE65*, *RDH5, RDH10*, *RDH11, BCO1 and ALDH1A3*. *LRAT* and *RPE65* act sequentially to catalyse the isomerization of all‐*trans*‐retinol to 11‐*cis*‐retinol. The RDH genes operate together to catalyse the oxidation of 11‐*cis*‐retinol to 11‐*cis*‐retinal,^50^ whereas *BCO1* catalyses the synthesis of all‐*trans* retinal. *ALDH1A3* is a retinoic acid synthesizing enzyme that may function in detoxifying retinal, either in a free or in a cellular retinol‐binding protein form. We also find that a number of other genes related to the visual cycle are up‐regulated with age including *RGR*, the retinoid carrier proteins (*RBP1*, *RBPL1*) and *SOX9* a key transcription factor for the visual cycle regulation.^51^ We also note that one of the age‐associated visual cycle genes, *RDH5*, was identified as one of the 15 putative causal genes for advanced AMD; a SNP (rs3138141) at the *RDH5* locus identified from an AMD GWAS influences expression of this gene in the RPE.^20^ Another one of the genes that we identified, *TTR*, also contains a SNP (rs1667255) discovered by a GWAS; in this case, the association is with circulating retinol/vitamin A levels.^52^ Thus, we provide further support for prioritization of these genes for future AMD therapeutic research.

Our finding of increased visual cycle transcription with increased age substantiates another avenue of innovative therapeutic research. There is a growing body of evidence supporting the use of pharmacological intervention to slow down the visual cycle in order to prevent lipofuscin accumulation.^53^ A properly functioning visual cycle gives rise to a number of natural by‐products. One category of such by‐products that have been shown to accumulate in aged wild‐type mice eyes are retinyl esters.^54^ Also, bisretinoids, including the well‐characterized A2E, are well documented to accumulate with increasing age in lipofuscin.^13^, ^55^ Indeed the accumulation of these lipofuscin bisretinoids is dependent on a normal visual cycle,^7^ and mice with an impaired visual cycle (Rpe65‐/‐) show less accumulation and are more resistant to light‐induced retinal degeneration.^56^ It was this work that first identified that the absence of retinaldehyde (both atRAL and 11‐*cis*) drastically reduced formation of lipofuscin fluorophores. Consequently, pharmaceutical research to slow down this accumulation has begun with the aim of preventing dry AMD/geographic atrophy.^57^ These pharmaceuticals known as visual cycle modulators (VCMs) include fenretinide, a synthetic retinoid, which reduces serum retinol and, therefore, inhibits the ocular uptake of all‐*trans*‐retinol, and emixustat hydrochloride, a small‐molecule inhibitor of the visual cycle isomerase *RPE65*. Another class of such pharmaceuticals is represented by the RBP4 antagonists that dissociate circulating RBP4‐TTR‐retinol complexes, reduce serum RBP4 levels and inhibit bisretinoid synthesis in models of enhanced retinal lipofuscinogenesis.^58^

We further present evidence that the visual cycle gene *LRAT* is transcriptionally up‐regulated in RPE in response to A2E, atRAL and Am580 (a RARα agonist) (Figure 4A,B). This finding is consistent with a number of observations from other studies that have studied the transcription of *LRAT*. In human prostate cells, retinoic acid receptors (RARs) activate transcription of *LRAT*.^59^ Also in the neonatal, lung retinoic acid (RA) synergizes with vitamin A to enhance *LRAT* expression ^60^ and Am580 dramatically increases *LRAT* expression.^61^ Dietary vitamin A has also been found to have a stimulatory effect on the expression of *LRAT* in the retina.^62^ Deprivation of vitamin A resulted in a significant reduction in *LRAT* expression in mice retina, whereas the *LRAT* expression was rescued in vitamin A deficient mice by intraperitoneal injection of RA.

Intriguingly, it has also been shown that RA is produced by the retina upon light exposure in mice.^63^ This corroborates well with recent in vitro evidence which found that all‐*trans*‐retinal is metabolized by RPE into both all‐*trans*‐retinol through reduction (via conventional visual cycle), and into *trans*‐RA through oxidation.^64^ Furthermore, the RARα‐selective agonist Am580, but not RXR agonists, has also been shown to stimulate the expression of the *SOX9* transcription factor in a cartilage‐derived cell line,^65^ various breast cancer cell lines^66^ and melanoma cells.^67^ Taken together this body of evidence suggests RA can accumulate in the RPE leading to activation of visual cycle expression through RAR and the SOX9 transcription factors. This is in line with our TF analysis that found the SCGs are enriched within SOX9 and RORB target genes. A final piece of important evidence consistent with our data is that A2E and atRAL have the capacity to activate RAR,^10^ with A2E inducing sustained activation of RAR target genes in ARPE19 cells.^68^

Taken together, these observations evoke a mechanism to explain, at least in part, our primary finding that visual cycle transcription increases with age. A2E and/or other visual cycle retinoid by‐products accumulate with age in the RPE, where they act as RAR‐ligands to stimulate transcription of *LRAT*. Collectively, the data suggest the existence of a novel mechanism involving a positive feedback loop between the transcription of visual cycle genes and the age‐related accumulation of visual cycle by‐products; age‐related increased retinoid by‐products accumulated from life‐time visual cycle activity stimulate increased transcription of key visual cycle gene(s) (Figure 4C). It remains to be elucidated whether this mechanism arises solely as a consequence of the ageing process or whether it may be part of a coping, adaptive process to maintain vision. A compellingly similar mechanism has been proposed previously to explain the cytoprotective effect of a pharmacological inhibitor of *CYP2C* involved in RA metabolism.^69^ This mechanism calls for further research into new interventions to slow down the accumulation of A2E/retinoid by‐products, for instance new VCMs that inhibit RAR‐activated gene transcription in the RPE.

The finding that there is a lack of spatial correlation between A2E and lipofuscin in the human retina, and that A2E is scarcely present in the macula^70^ casted some doubt over it being a direct contributor to macular degeneration pathogenesis. However, this does not preclude A2E from being a transitory intermediate in this pathogenic process; indeed studies have shown that bisretinoids undergo photo‐oxidation induced degradation when irradiated with short‐wavelength visible (blue) light.^71^ Photo‐degradation of A2E and all‐*trans*‐retinal dimer generates the dicarbonyls glyoxal and methylglyoxal, known to form advanced glycation end products (AGEs).^15^ This observation corresponds well with the fact that AGE accumulation on the Bruch's membrane increases with age.^16^, ^72^ Importantly, such accumulation of AGEs has been shown to affect RPE function^42^ and has been linked with AMD.^73^ Thus the accumulation of certain atRAL condensation products followed by their photo‐degradation, leading to AGE formation may represent one mechanism of atrophic macular degeneration.

In conclusion, even though the exact process by which accumulated visual cycle by‐products lead to retinal degeneration remains to be confirmed, our study, besides revealing the unexpected age‐related increase in visual cycle gene transcription, presents further evidence that accumulated visual cycle by‐products can actually activate visual cycle gene expression, thus exacerbating their own accumulation.

## CONFLICT OF INTEREST

The authors confirm that there are no conflicts of interest.

## AUTHOR CONTRIBUTIONS

**Joe M. Butler:** Conceptualization (equal); Data curation (lead); Formal analysis (equal); Investigation (equal); Methodology (lead); Validation (supporting); Visualization (lead); Writing‐original draft (lead); Writing‐review & editing (equal). **Wasu Supharattanasitthi:** Formal analysis (supporting); Investigation (supporting); Methodology (supporting); Validation (equal); Writing‐original draft (supporting); Writing‐review & editing (supporting). **Yit C. Yang:** Conceptualization (supporting); Funding acquisition (equal); Investigation (supporting); Resources (equal); Supervision (supporting); Writing‐review & editing (supporting). **Luminita Paraoan:** Conceptualization (lead); Data curation (supporting); Formal analysis (supporting); Funding acquisition (lead); Investigation (equal); Methodology (supporting); Project administration (lead); Resources (lead); Software (supporting); Supervision (lead); Validation (supporting); Visualization (supporting); Writing‐original draft (supporting); Writing‐review & editing (equal).

## Supporting information

Figure S1

Table S1

Table S2

Table S3

## Data Availability

The raw sequence data and processed counts data were deposited in the GEO repository (https://www.ncbi.nlm.nih.gov/geo/) under accession number GSE159435.
