# Supplementary material for: RNA‐seq analysis of ageing human retinal pigment epithelium: Unexpected up‐regulation of visual cycle gene transcription
Source: J Cell Mol Med. 2021 May 1;25(12):5572–85. doi: 10.1111/jcmm.16569 (PMC8184696; doi:10.1111/jcmm.16569)
Supplement: Supplementary file 1 — Figure S1 [file JCMM-25-5572-s004.pdf]

Fig S1

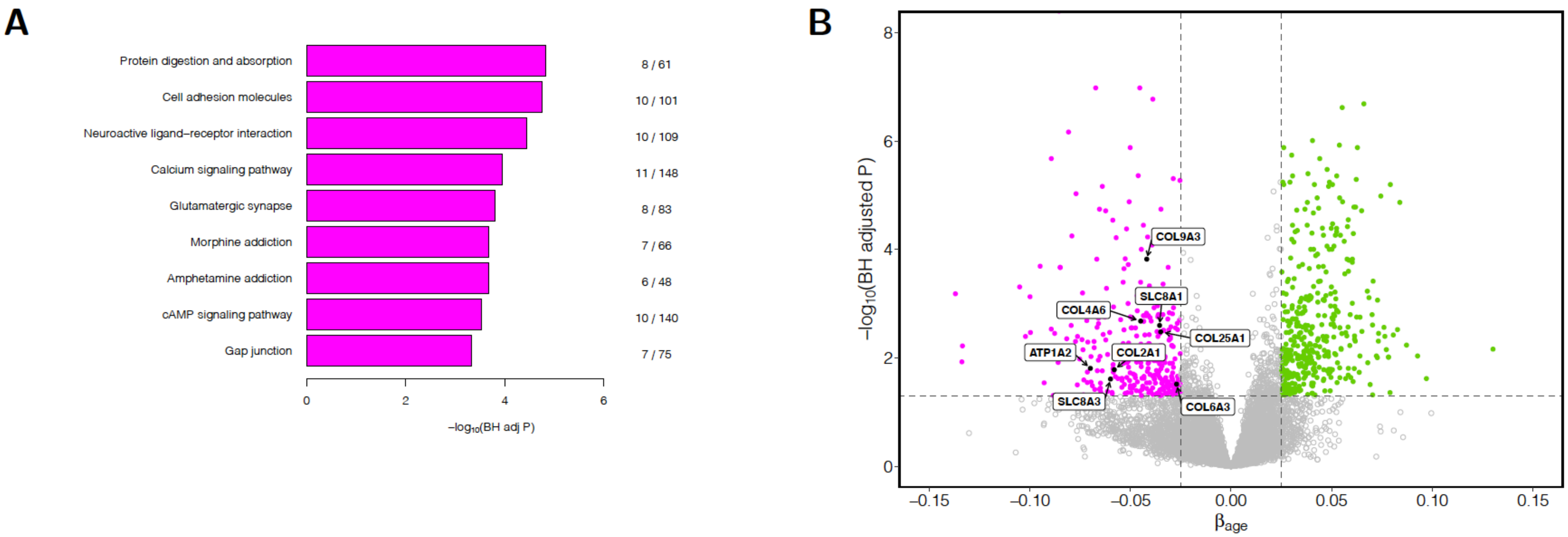

Fig. S1. RNA-seq of RPE reveals many pathways are transcriptionally down-regulated with increasing human age. (A) The top most significantly enriched pathways with down-regulated SCGs. All pathways shown have a BH adjusted P < 0.05. Fraction represents the number of down SCGs in the pathway over the total number of genes in the pathway. (*B*) Volcano plot showing up-regulated SCGs in green and down-regulated in magenta. Genes in the *protein digestion and absorption* KEGG pathway are labelled.
