# Supplementary material for: RNA‐seq analysis of ageing human retinal pigment epithelium: Unexpected up‐regulation of visual cycle gene transcription
Source: J Cell Mol Med. 2021 May 1;25(12):5572–85. doi: 10.1111/jcmm.16569 (PMC8184696; doi:10.1111/jcmm.16569)
Supplement: Supplementary file 2 — Table S1 [file JCMM-25-5572-s003.docx]

**Table S1. Sequences of primers used in qPCR validation**

| **Gene** | **Sequence** | |
| --- | --- | --- |
| *LRAT* | Forward:  Reverse: | 5’ TGCGAGCACTTCGTGACCTACT 3’  3’ GCCAATCCCAAGACTGCTGAAG 5’ |
| *RPE65* | Forward:  Reverse: | 5’ TTTGGCACCTGTGCTTTCCCAG 3’  3’ GTTGGTCTCTGTGCAAGCGTAG 5’ |
| *RDH5* | Forward:  Reverse: | 5’ CTGTGACCAACCTGGAGAGTCT 3’  3’ GATGCGCTGTTGCATTTTCAGGT 5’ |
| *GAPDH* | Forward:  Reverse: | 5’ TTGCCCTCAACGACCACTTT 3’  3’ TGGTCCAGGGGTCTTACTCC 5’ |
| *RPL5* | Forward:  Reverse: | 5’ ATGCTCGGAAACGCTTGGT 3’  3’ GCGCAGACTATCATATCCCCC 5’ |
| *ACTB* | Forward:  Reverse: | 5’ CACCATTGGCAATGAGCGGTTC 3’  3’ AGGTCTTTGCGGATGTCCACGT 5’ |
| *TUBB* | Forward:  Reverse: | 5’ CTGGACCGCATCTCTGTGTACT 3’  3’ GCCAAAAGGACCTGAGCGAACA 5’ |
