# Supplementary material for: RNA‐seq analysis of ageing human retinal pigment epithelium: Unexpected up‐regulation of visual cycle gene transcription
Source: J Cell Mol Med. 2021 May 1;25(12):5572–85. doi: 10.1111/jcmm.16569 (PMC8184696; doi:10.1111/jcmm.16569)
Supplement: Supplementary file 4 — Table S3 [file JCMM-25-5572-s002.docx]

**Table S2. List of all genes significantly correlated with age**

|  | **ENSEMBL** | **baseMean** | **Beta** | **BetaSE** | **stat** | **pvalue** | **padj** | **gene_biotype** | **SYMBOL** | **ENTREZID** |
| --- | --- | --- | --- | --- | --- | --- | --- | --- | --- | --- |
| 1 | ENSG00000007402 | 263.03 | -2.84E-02 | 3.81E-03 | -7.456 | 8.92E-14 | 1.00E-09 | protein_coding | CACNA2D2 | 9254 |
| 2 | ENSG00000165905 | 140.34 | 5.16E-02 | 6.93E-03 | 7.442 | 9.93E-14 | 1.00E-09 | protein_coding | LARGE2 | 120071 |
| 3 | ENSG00000181577 | 84.35 | 6.44E-02 | 8.89E-03 | 7.251 | 4.15E-13 | 2.79E-09 | lncRNA | C6orf223 | 221416 |
| 4 | ENSG00000170959 | 192.03 | -8.54E-02 | 1.19E-02 | -7.162 | 7.95E-13 | 4.01E-09 | protein_coding | DCDC1 | 341019 |
| 5 | ENSG00000021645 | 279.35 | -6.72E-02 | 1.01E-02 | -6.661 | 2.71E-11 | 1.04E-07 | protein_coding | NRXN3 | 9369 |
| 6 | ENSG00000154310 | 541.64 | -4.53E-02 | 6.82E-03 | -6.641 | 3.11E-11 | 1.04E-07 | protein_coding | TNIK | 23043 |
| 7 | ENSG00000126218 | 65.41 | -3.88E-02 | 5.93E-03 | -6.547 | 5.86E-11 | 1.69E-07 | protein_coding | F10 | 2159 |
| 8 | ENSG00000253417 | 1742.46 | 6.60E-02 | 1.02E-02 | 6.498 | 8.16E-11 | 2.06E-07 | lncRNA | LINC02159 | 285629 |
| 9 | ENSG00000173898 | 3239.57 | 5.52E-02 | 8.55E-03 | 6.456 | 1.08E-10 | 2.41E-07 | protein_coding | SPTBN2 | 6712 |
| 10 | ENSG00000152578 | 292.29 | -8.07E-02 | 1.28E-02 | -6.280 | 3.38E-10 | 6.81E-07 | protein_coding | GRIA4 | 2893 |
| 11 | ENSG00000103187 | 1572.87 | 4.04E-02 | 6.51E-03 | 6.208 | 5.35E-10 | 9.80E-07 | protein_coding | COTL1 | 23406 |
| 12 | ENSG00000167995 | 23076.85 | 5.38E-02 | 8.73E-03 | 6.164 | 7.08E-10 | 1.19E-06 | protein_coding | BEST1 | 7439 |
| 13 | ENSG00000130429 | 272.78 | 2.62E-02 | 4.27E-03 | 6.120 | 9.34E-10 | 1.32E-06 | protein_coding | ARPC1B | 10095 |
| 14 | ENSG00000175183 | 567.08 | -5.01E-02 | 8.18E-03 | -6.119 | 9.41E-10 | 1.32E-06 | protein_coding | CSRP2 | 1466 |
| 15 | ENSG00000148604 | 11926.22 | 6.28E-02 | 1.03E-02 | 6.112 | 9.82E-10 | 1.32E-06 | protein_coding | RGR | 5995 |
| 16 | ENSG00000258311 | 63.32 | 6.30E-02 | 1.04E-02 | 6.042 | 1.52E-09 | 1.82E-06 | protein_coding |  |  |
| 17 | ENSG00000164889 | 2167.72 | 3.01E-02 | 4.99E-03 | 6.041 | 1.54E-09 | 1.82E-06 | protein_coding | SLC4A2 | 6522 |
| 18 | ENSG00000101203 | 183.28 | 4.40E-02 | 7.34E-03 | 6.002 | 1.95E-09 | 2.11E-06 | protein_coding | COL20A1 | 57642 |
| 19 | ENSG00000174827 | 23.47 | -8.92E-02 | 1.49E-02 | -5.999 | 1.99E-09 | 2.11E-06 | protein_coding | PDZK1 | 5174 |
| 20 | ENSG00000230448 | 962.54 | 4.77E-02 | 8.07E-03 | 5.915 | 3.31E-09 | 3.34E-06 | lncRNA | LINC00276 | 100499171 |
| 21 | ENSG00000200818 | 88.27 | 3.82E-02 | 6.50E-03 | 5.878 | 4.16E-09 | 4.00E-06 | snRNA | RNU6-1204P | 106481560 |
| 22 | ENSG00000243089 | 76.97 | 4.19E-02 | 7.16E-03 | 5.851 | 4.87E-09 | 4.35E-06 | lncRNA |  |  |
| 23 | ENSG00000249669 | 66.99 | -7.69E-02 | 1.32E-02 | -5.837 | 5.30E-09 | 4.35E-06 | lncRNA |  |  |
| 24 | ENSG00000181234 | 249.12 | -4.61E-02 | 7.90E-03 | -5.837 | 5.33E-09 | 4.35E-06 | protein_coding | TMEM132C | 92293 |
| 25 | ENSG00000255440 | 11.06 | 8.12E-02 | 1.39E-02 | 5.835 | 5.39E-09 | 4.35E-06 | lncRNA |  |  |
| 26 | ENSG00000169031 | 2341.37 | 3.06E-02 | 5.25E-03 | 5.825 | 5.72E-09 | 4.40E-06 | protein_coding | COL4A3 | 1285 |
| 27 | ENSG00000132376 | 4395.33 | 5.25E-02 | 9.02E-03 | 5.820 | 5.89E-09 | 4.40E-06 | protein_coding | INPP5K | 51763 |
| 28 | ENSG00000079819 | 4128.40 | -2.87E-02 | 4.95E-03 | -5.795 | 6.85E-09 | 4.93E-06 | protein_coding | EPB41L2 | 2037 |
| 29 | ENSG00000160183 | 28.96 | 6.22E-02 | 1.07E-02 | 5.783 | 7.34E-09 | 5.10E-06 | protein_coding | TMPRSS3 | 64699 |
| 30 | ENSG00000166292 | 130.84 | -2.54E-02 | 4.40E-03 | -5.770 | 7.93E-09 | 5.33E-06 | protein_coding | TMEM100 | 55273 |
| 31 | ENSG00000140950 | 388.86 | 2.57E-02 | 4.47E-03 | 5.752 | 8.84E-09 | 5.73E-06 | protein_coding | MEAK7 | 57707 |
| 32 | ENSG00000100344 | 1603.36 | 4.89E-02 | 8.51E-03 | 5.739 | 9.51E-09 | 5.73E-06 | protein_coding | PNPLA3 | 80339 |
| 33 | ENSG00000169710 | 745.51 | 2.93E-02 | 5.10E-03 | 5.738 | 9.57E-09 | 5.73E-06 | protein_coding | FASN | 2194 |
| 34 | ENSG00000183111 | 1077.22 | 2.62E-02 | 4.59E-03 | 5.706 | 1.16E-08 | 6.34E-06 | protein_coding | ARHGEF37 | 389337 |
| 35 | ENSG00000084453 | 1890.19 | 7.91E-02 | 1.39E-02 | 5.702 | 1.18E-08 | 6.34E-06 | protein_coding | SLCO1A2 | 6579 |
| 36 | ENSG00000114859 | 325.21 | 4.14E-02 | 7.26E-03 | 5.700 | 1.20E-08 | 6.34E-06 | protein_coding | CLCN2 | 1181 |
| 37 | ENSG00000260423 | 65.41 | 5.04E-02 | 8.84E-03 | 5.700 | 1.20E-08 | 6.34E-06 | lncRNA | LINC02367 | 101930452 |
| 38 | ENSG00000189283 | 1540.89 | 4.84E-02 | 8.52E-03 | 5.683 | 1.33E-08 | 6.86E-06 | protein_coding | FHIT | 2272 |
| 39 | ENSG00000152208 | 85.36 | -6.39E-02 | 1.13E-02 | -5.678 | 1.36E-08 | 6.86E-06 | protein_coding | GRID2 | 2895 |
| 40 | ENSG00000174963 | 19.76 | -7.70E-02 | 1.37E-02 | -5.616 | 1.95E-08 | 9.36E-06 | protein_coding | ZIC4 | 84107 |
| 41 | ENSG00000163053 | 4624.65 | 7.44E-02 | 1.33E-02 | 5.595 | 2.21E-08 | 1.03E-05 | protein_coding | SLC16A14 | 151473 |
| 42 | ENSG00000261452 | 1545.80 | 5.64E-02 | 1.01E-02 | 5.572 | 2.51E-08 | 1.12E-05 | NA |  |  |
| 43 | ENSG00000177133 | 1506.29 | 4.28E-02 | 7.67E-03 | 5.572 | 2.52E-08 | 1.12E-05 | lncRNA | PRDM16-DT | 440556 |
| 44 | ENSG00000159212 | 6018.59 | 5.39E-02 | 9.69E-03 | 5.568 | 2.57E-08 | 1.12E-05 | protein_coding | CLIC6 | 54102 |
| 45 | ENSG00000136943 | 975.15 | 5.54E-02 | 1.00E-02 | 5.532 | 3.17E-08 | 1.32E-05 | protein_coding | CTSV | 1515 |
| 46 | ENSG00000171951 | 178.52 | -5.06E-02 | 9.14E-03 | -5.530 | 3.20E-08 | 1.32E-05 | protein_coding | SCG2 | 7857 |
| 47 | ENSG00000182218 | 266.37 | 3.79E-02 | 6.87E-03 | 5.520 | 3.39E-08 | 1.35E-05 | protein_coding | HHIPL1 | 84439 |
| 48 | ENSG00000163898 | 108.17 | 8.38E-02 | 1.52E-02 | 5.518 | 3.43E-08 | 1.35E-05 | protein_coding | LIPH | 200879 |
| 49 | ENSG00000196660 | 273.43 | 6.18E-02 | 1.13E-02 | 5.478 | 4.29E-08 | 1.65E-05 | protein_coding | SLC30A10 | 55532 |
| 50 | ENSG00000127472 | 920.69 | 6.10E-02 | 1.11E-02 | 5.476 | 4.34E-08 | 1.65E-05 | protein_coding | PLA2G5 | 5322 |
| 51 | ENSG00000162105 | 1160.15 | 4.35E-02 | 7.96E-03 | 5.465 | 4.64E-08 | 1.73E-05 | protein_coding | SHANK2 | 22941 |
| 52 | ENSG00000102445 | 40.51 | 3.67E-02 | 6.73E-03 | 5.452 | 4.97E-08 | 1.81E-05 | protein_coding | RUBCNL | 80183 |
| 53 | ENSG00000165959 | 193.24 | -3.48E-02 | 6.39E-03 | -5.451 | 5.02E-08 | 1.81E-05 | protein_coding | CLMN | 79789 |
| 54 | ENSG00000152977 | 191.72 | -6.53E-02 | 1.20E-02 | -5.448 | 5.11E-08 | 1.81E-05 | protein_coding | ZIC1 | 7545 |
| 55 | ENSG00000136059 | 164.39 | 3.26E-02 | 6.00E-03 | 5.437 | 5.41E-08 | 1.88E-05 | protein_coding | VILL | 50853 |
| 56 | ENSG00000100156 | 462.04 | 6.48E-02 | 1.19E-02 | 5.429 | 5.65E-08 | 1.93E-05 | protein_coding | SLC16A8 | 23539 |
| 57 | ENSG00000112139 | 82.36 | -6.22E-02 | 1.15E-02 | -5.426 | 5.76E-08 | 1.94E-05 | protein_coding | MDGA1 | 266727 |
| 58 | ENSG00000067798 | 2428.13 | 4.09E-02 | 7.56E-03 | 5.407 | 6.42E-08 | 2.12E-05 | protein_coding | NAV3 | 89795 |
| 59 | ENSG00000253778 | 141.39 | 4.72E-02 | 8.81E-03 | 5.357 | 8.48E-08 | 2.76E-05 | lncRNA |  |  |
| 60 | ENSG00000135437 | 447.63 | 5.82E-02 | 1.09E-02 | 5.348 | 8.87E-08 | 2.84E-05 | protein_coding | RDH5 | 5959 |
| 61 | ENSG00000196104 | 170.24 | -5.87E-02 | 1.10E-02 | -5.343 | 9.15E-08 | 2.88E-05 | protein_coding | SPOCK3 | 50859 |
| 62 | ENSG00000259126 | 54.74 | 5.93E-02 | 1.12E-02 | 5.317 | 1.06E-07 | 3.28E-05 | lncRNA |  |  |
| 63 | ENSG00000196189 | 918.94 | 5.04E-02 | 9.49E-03 | 5.309 | 1.10E-07 | 3.36E-05 | protein_coding | SEMA4A | 64218 |
| 64 | ENSG00000068724 | 575.97 | 3.05E-02 | 5.77E-03 | 5.294 | 1.20E-07 | 3.57E-05 | protein_coding | TTC7A | 57217 |
| 65 | ENSG00000100918 | 229.30 | 3.78E-02 | 7.15E-03 | 5.290 | 1.22E-07 | 3.57E-05 | protein_coding | REC8 | 9985 |
| 66 | ENSG00000125618 | 21.43 | -4.35E-02 | 8.23E-03 | -5.290 | 1.22E-07 | 3.57E-05 | protein_coding | PAX8 | 7849 |
| 67 | ENSG00000140522 | 14620.98 | 5.78E-02 | 1.10E-02 | 5.274 | 1.33E-07 | 3.79E-05 | protein_coding | RLBP1 | 6017 |
| 68 | ENSG00000185818 | 66.83 | 4.54E-02 | 8.63E-03 | 5.259 | 1.45E-07 | 4.05E-05 | protein_coding | NAT8L | 339983 |
| 69 | ENSG00000152936 | 134.34 | 4.98E-02 | 9.47E-03 | 5.253 | 1.49E-07 | 4.07E-05 | protein_coding | LMNTD1 | 160492 |
| 70 | ENSG00000230453 | 56.46 | 5.24E-02 | 9.98E-03 | 5.253 | 1.50E-07 | 4.07E-05 | protein_coding | ANKRD18B | 441459 |
| 71 | ENSG00000079156 | 321.82 | -5.19E-02 | 9.89E-03 | -5.246 | 1.55E-07 | 4.17E-05 | protein_coding | OSBPL6 | 114880 |
| 72 | ENSG00000213988 | 113.94 | 3.29E-02 | 6.29E-03 | 5.231 | 1.68E-07 | 4.47E-05 | protein_coding | ZNF90 | 7643 |
| 73 | ENSG00000176571 | 193.96 | 5.25E-02 | 1.01E-02 | 5.216 | 1.83E-07 | 4.71E-05 | protein_coding | CNBD1 | 168975 |
| 74 | ENSG00000262943 | 133.02 | 3.19E-02 | 6.11E-03 | 5.214 | 1.85E-07 | 4.71E-05 | transcribed_unprocessed_pseudogene | ALOX12P2 | 245 |
| 75 | ENSG00000155265 | 76.20 | 4.02E-02 | 7.72E-03 | 5.201 | 1.98E-07 | 4.99E-05 | protein_coding | GOLGA7B | 401647 |
| 76 | ENSG00000175344 | 98.08 | 6.07E-02 | 1.17E-02 | 5.195 | 2.04E-07 | 5.08E-05 | protein_coding | CHRNA7 | 1139 |
| 77 | ENSG00000100399 | 203.28 | 4.59E-02 | 8.86E-03 | 5.179 | 2.24E-07 | 5.46E-05 | protein_coding | CHADL | 150356 |
| 78 | ENSG00000164007 | 446.12 | 5.43E-02 | 1.05E-02 | 5.177 | 2.26E-07 | 5.46E-05 | protein_coding | CLDN19 | 149461 |
| 79 | ENSG00000065675 | 870.74 | 5.18E-02 | 1.00E-02 | 5.175 | 2.28E-07 | 5.46E-05 | protein_coding | PRKCQ | 5588 |
| 80 | ENSG00000141750 | 189.38 | -7.90E-02 | 1.53E-02 | -5.167 | 2.38E-07 | 5.64E-05 | protein_coding | STAC2 | 342667 |
| 81 | ENSG00000182168 | 384.33 | -4.14E-02 | 8.03E-03 | -5.157 | 2.51E-07 | 5.88E-05 | protein_coding | UNC5C | 8633 |
| 82 | ENSG00000189223 | 197.00 | -5.70E-02 | 1.11E-02 | -5.149 | 2.62E-07 | 6.07E-05 | lncRNA | PAX8-AS1 | 654433 |
| 83 | ENSG00000183423 | 415.68 | 3.02E-02 | 5.88E-03 | 5.132 | 2.87E-07 | 6.49E-05 | protein_coding | LRIT3 | 345193 |
| 84 | ENSG00000167332 | 489.20 | 5.62E-02 | 1.10E-02 | 5.114 | 3.16E-07 | 7.08E-05 | protein_coding | OR51E2 | 81285 |
| 85 | ENSG00000166965 | 278.37 | 3.12E-02 | 6.11E-03 | 5.105 | 3.30E-07 | 7.31E-05 | protein_coding | RCCD1 | 91433 |
| 86 | ENSG00000255028 | 36.84 | 4.97E-02 | 9.75E-03 | 5.094 | 3.50E-07 | 7.68E-05 | lncRNA |  |  |
| 87 | ENSG00000169760 | 399.15 | -3.92E-02 | 7.73E-03 | -5.077 | 3.83E-07 | 8.30E-05 | protein_coding | NLGN1 | 22871 |
| 88 | ENSG00000246379 | 30.88 | 4.77E-02 | 9.41E-03 | 5.065 | 4.08E-07 | 8.68E-05 | lncRNA | LOC283856 | 283856 |
| 89 | ENSG00000084710 | 2769.55 | 5.11E-02 | 1.01E-02 | 5.065 | 4.09E-07 | 8.68E-05 | protein_coding | EFR3B | 22979 |
| 90 | ENSG00000119121 | 151.53 | 5.01E-02 | 9.96E-03 | 5.033 | 4.82E-07 | 9.91E-05 | protein_coding | TRPM6 | 140803 |
| 91 | ENSG00000204128 | 90.45 | 4.88E-02 | 9.69E-03 | 5.030 | 4.90E-07 | 9.95E-05 | protein_coding | C2orf72 | 257407 |
| 92 | ENSG00000248550 | 714.72 | 3.95E-02 | 7.87E-03 | 5.026 | 5.02E-07 | 9.95E-05 | lncRNA | OTX2-AS1 | 100309464 |
| 93 | ENSG00000126500 | 27.88 | -4.45E-02 | 8.86E-03 | -5.025 | 5.03E-07 | 9.95E-05 | protein_coding | FLRT1 | 23769 |
| 94 | ENSG00000120262 | 308.85 | 4.10E-02 | 8.16E-03 | 5.025 | 5.04E-07 | 9.95E-05 | protein_coding | CCDC170 | 80129 |
| 95 | ENSG00000100003 | 1033.04 | 4.30E-02 | 8.62E-03 | 4.982 | 6.28E-07 | 0.00012178 | protein_coding | SEC14L2 | 23541 |
| 96 | ENSG00000133116 | 188.88 | 4.20E-02 | 8.46E-03 | 4.967 | 6.79E-07 | 0.00013029 | protein_coding | KL | 9365 |
| 97 | ENSG00000251637 | 99.04 | 4.26E-02 | 8.59E-03 | 4.965 | 6.88E-07 | 0.00013079 | lncRNA |  |  |
| 98 | ENSG00000125378 | 1677.11 | 2.96E-02 | 5.99E-03 | 4.945 | 7.63E-07 | 0.00014378 | protein_coding | BMP4 | 652 |
| 99 | ENSG00000235545 | 96.41 | 4.91E-02 | 9.94E-03 | 4.942 | 7.71E-07 | 0.00014395 | lncRNA |  |  |
| 100 | ENSG00000115616 | 34.37 | -5.25E-02 | 1.06E-02 | -4.935 | 8.03E-07 | 0.00014843 | protein_coding | SLC9A2 | 6549 |
| 101 | ENSG00000152822 | 99.06 | -6.67E-02 | 1.35E-02 | -4.929 | 8.28E-07 | 0.00015091 | protein_coding | GRM1 | 2911 |
| 102 | ENSG00000183273 | 128.44 | 6.02E-02 | 1.22E-02 | 4.928 | 8.32E-07 | 0.00015091 | protein_coding | CCDC60 | 160777 |
| 103 | ENSG00000092758 | 443.93 | -4.18E-02 | 8.50E-03 | -4.925 | 8.46E-07 | 0.00015091 | protein_coding | COL9A3 | 1299 |
| 104 | ENSG00000211787 | 39.89 | 5.76E-02 | 1.17E-02 | 4.924 | 8.46E-07 | 0.00015091 | TR_V_gene | TRAV8-3 | 28683 |
| 105 | ENSG00000140015 | 668.96 | 5.86E-02 | 1.19E-02 | 4.912 | 9.01E-07 | 0.00015796 | protein_coding | KCNH5 | 27133 |
| 106 | ENSG00000235912 | 41.18 | 3.25E-02 | 6.62E-03 | 4.909 | 9.14E-07 | 0.00015887 | processed_pseudogene |  |  |
| 107 | ENSG00000089356 | 247.72 | 6.02E-02 | 1.23E-02 | 4.891 | 1.00E-06 | 0.00017261 | protein_coding | FXYD3 | 5349 |
| 108 | ENSG00000258395 | 7.02 | -1.08E-01 | 2.22E-02 | -4.874 | 1.10E-06 | 0.00018712 | NA |  |  |
| 109 | ENSG00000197142 | 1932.61 | 3.55E-02 | 7.29E-03 | 4.870 | 1.12E-06 | 0.00018932 | protein_coding | ACSL5 | 51703 |
| 110 | ENSG00000158008 | 21.02 | -5.10E-02 | 1.05E-02 | -4.867 | 1.13E-06 | 0.00018988 | protein_coding | EXTL1 | 2134 |
| 111 | ENSG00000172987 | 8.56 | -9.48E-02 | 1.95E-02 | -4.852 | 1.22E-06 | 0.00020357 | protein_coding | HPSE2 | 60495 |
| 112 | ENSG00000124920 | 1779.13 | 4.58E-02 | 9.44E-03 | 4.848 | 1.25E-06 | 0.00020568 | protein_coding | MYRF | 745 |
| 113 | ENSG00000151388 | 122.91 | 3.37E-02 | 6.95E-03 | 4.846 | 1.26E-06 | 0.00020611 | protein_coding | ADAMTS12 | 81792 |
| 114 | ENSG00000157087 | 1126.02 | 4.35E-02 | 8.98E-03 | 4.842 | 1.29E-06 | 0.00020889 | protein_coding | ATP2B2 | 491 |
| 115 | ENSG00000077264 | 203.36 | -3.12E-02 | 6.46E-03 | -4.836 | 1.33E-06 | 0.00021389 | protein_coding | PAK3 | 5063 |
| 116 | ENSG00000259905 | 8.35 | -8.48E-02 | 1.75E-02 | -4.834 | 1.34E-06 | 0.00021442 | lncRNA | PWRN1 | 791114 |
| 117 | ENSG00000174611 | 64.53 | 3.89E-02 | 8.06E-03 | 4.822 | 1.42E-06 | 0.00022504 | protein_coding | KY | 339855 |
| 118 | ENSG00000140470 | 63.67 | -5.31E-02 | 1.10E-02 | -4.817 | 1.45E-06 | 0.00022606 | protein_coding | ADAMTS17 | 170691 |
| 119 | ENSG00000120057 | 3483.49 | 5.83E-02 | 1.22E-02 | 4.793 | 1.64E-06 | 0.00025253 | protein_coding | SFRP5 | 6425 |
| 120 | ENSG00000177283 | 384.34 | 2.52E-02 | 5.28E-03 | 4.786 | 1.70E-06 | 0.00026009 | protein_coding | FZD8 | 8325 |
| 121 | ENSG00000119927 | 4067.71 | 4.72E-02 | 9.87E-03 | 4.784 | 1.72E-06 | 0.0002601 | protein_coding | GPAM | 57678 |
| 122 | ENSG00000171208 | 1338.54 | 5.65E-02 | 1.19E-02 | 4.766 | 1.88E-06 | 0.0002819 | protein_coding | NETO2 | 81831 |
| 123 | ENSG00000189129 | 302.38 | 3.14E-02 | 6.64E-03 | 4.721 | 2.35E-06 | 0.00034577 | protein_coding | PLAC9 | 219348 |
| 124 | ENSG00000092439 | 8395.15 | 2.85E-02 | 6.04E-03 | 4.714 | 2.43E-06 | 0.00035505 | protein_coding | TRPM7 | 54822 |
| 125 | ENSG00000125398 | 2273.75 | 4.43E-02 | 9.44E-03 | 4.696 | 2.66E-06 | 0.00038332 | protein_coding | SOX9 | 6662 |
| 126 | ENSG00000241022 | 12.14 | 6.41E-02 | 1.36E-02 | 4.695 | 2.66E-06 | 0.00038332 | processed_pseudogene |  |  |
| 127 | ENSG00000178772 | 43.11 | 7.05E-02 | 1.50E-02 | 4.693 | 2.69E-06 | 0.00038465 | protein_coding | CPN2 | 1370 |
| 128 | ENSG00000127249 | 448.31 | 4.34E-02 | 9.27E-03 | 4.689 | 2.75E-06 | 0.00039033 | protein_coding | ATP13A4 | 84239 |
| 129 | ENSG00000144791 | 837.98 | 2.80E-02 | 5.97E-03 | 4.687 | 2.77E-06 | 0.00039095 | protein_coding | LIMD1 | 8994 |
| 130 | ENSG00000214548 | 266.04 | -4.49E-02 | 9.59E-03 | -4.679 | 2.88E-06 | 0.0004024 | lncRNA | MEG3 | 55384 |
| 131 | ENSG00000157890 | 159.49 | -5.36E-02 | 1.14E-02 | -4.678 | 2.90E-06 | 0.0004024 | protein_coding | MEGF11 | 84465 |
| 132 | ENSG00000180447 | 477.85 | -3.37E-02 | 7.24E-03 | -4.660 | 3.16E-06 | 0.00043578 | protein_coding | GAS1 | 2619 |
| 133 | ENSG00000179954 | 33.16 | -4.05E-02 | 8.73E-03 | -4.643 | 3.43E-06 | 0.00046969 | protein_coding | SSC5D | 284297 |
| 134 | ENSG00000268119 | 109.50 | 4.10E-02 | 8.84E-03 | 4.637 | 3.54E-06 | 0.00048206 | lncRNA | LOC105372321 | 105372321 |
| 135 | ENSG00000142549 | 9.80 | -1.05E-01 | 2.27E-02 | -4.631 | 3.63E-06 | 0.00049126 | protein_coding | IGLON5 | 402665 |
| 136 | ENSG00000101076 | 269.76 | 4.98E-02 | 1.08E-02 | 4.630 | 3.66E-06 | 0.00049126 | protein_coding | HNF4A | 3172 |
| 137 | ENSG00000173638 | 256.68 | 3.06E-02 | 6.61E-03 | 4.625 | 3.75E-06 | 0.00050048 | protein_coding | SLC19A1 | 6573 |
| 138 | ENSG00000172318 | 44.77 | -6.18E-02 | 1.34E-02 | -4.615 | 3.94E-06 | 0.00052184 | protein_coding | B3GALT1 | 8708 |
| 139 | ENSG00000254631 | 12.16 | 7.54E-02 | 1.64E-02 | 4.607 | 4.08E-06 | 0.0005377 | lncRNA |  |  |
| 140 | ENSG00000189212 | 20.78 | -6.44E-02 | 1.40E-02 | -4.596 | 4.30E-06 | 0.00055815 | transcribed_unprocessed_pseudogene |  |  |
| 141 | ENSG00000151692 | 455.76 | -2.62E-02 | 5.72E-03 | -4.584 | 4.57E-06 | 0.00058606 | protein_coding | RNF144A | 9781 |
| 142 | ENSG00000116985 | 105.32 | 6.77E-02 | 1.48E-02 | 4.582 | 4.62E-06 | 0.00058883 | protein_coding | BMP8B | 656 |
| 143 | ENSG00000114115 | 3524.18 | 5.16E-02 | 1.13E-02 | 4.572 | 4.83E-06 | 0.00061216 | protein_coding | RBP1 | 5947 |
| 144 | ENSG00000075035 | 198.95 | -7.37E-02 | 1.62E-02 | -4.563 | 5.04E-06 | 0.00063537 | protein_coding | WSCD2 | 9671 |
| 145 | ENSG00000254890 | 12.08 | 6.45E-02 | 1.41E-02 | 4.561 | 5.09E-06 | 0.00063593 | processed_pseudogene |  |  |
| 146 | ENSG00000138798 | 1212.55 | 2.59E-02 | 5.68E-03 | 4.560 | 5.11E-06 | 0.00063593 | protein_coding | EGF | 1950 |
| 147 | ENSG00000117984 | 5468.61 | 4.30E-02 | 9.44E-03 | 4.558 | 5.17E-06 | 0.00063863 | protein_coding | CTSD | 1509 |
| 148 | ENSG00000254102 | 7.24 | -1.37E-01 | 3.01E-02 | -4.550 | 5.36E-06 | 0.00065691 | lncRNA | LOC401463 | 401463 |
| 149 | ENSG00000149452 | 2281.69 | 4.95E-02 | 1.09E-02 | 4.548 | 5.41E-06 | 0.00065706 | protein_coding | SLC22A8 | 9376 |
| 150 | ENSG00000100350 | 275.16 | 2.74E-02 | 6.03E-03 | 4.542 | 5.56E-06 | 0.00066735 | protein_coding | FOXRED2 | 80020 |
| 151 | ENSG00000232044 | 635.26 | 5.20E-02 | 1.15E-02 | 4.542 | 5.58E-06 | 0.00066735 | lncRNA |  |  |
| 152 | ENSG00000233705 | 313.16 | 5.88E-02 | 1.29E-02 | 4.541 | 5.60E-06 | 0.00066735 | lncRNA |  |  |
| 153 | ENSG00000141526 | 1362.28 | 4.71E-02 | 1.04E-02 | 4.525 | 6.05E-06 | 0.00071096 | protein_coding | SLC16A3 | 9123 |
| 154 | ENSG00000150672 | 1218.65 | -4.66E-02 | 1.03E-02 | -4.524 | 6.07E-06 | 0.00071096 | protein_coding | DLG2 | 1740 |
| 155 | ENSG00000138771 | 1629.82 | 3.92E-02 | 8.67E-03 | 4.522 | 6.13E-06 | 0.00071471 | protein_coding | SHROOM3 | 57619 |
| 156 | ENSG00000009694 | 370.87 | -4.57E-02 | 1.01E-02 | -4.518 | 6.25E-06 | 0.0007195 | protein_coding | TENM1 | 10178 |
| 157 | ENSG00000010379 | 4669.01 | 3.73E-02 | 8.28E-03 | 4.508 | 6.54E-06 | 0.00074528 | protein_coding | SLC6A13 | 6540 |
| 158 | ENSG00000116690 | 213.44 | -9.98E-02 | 2.21E-02 | -4.508 | 6.55E-06 | 0.00074528 | protein_coding | PRG4 | 10216 |
| 159 | ENSG00000225857 | 93.32 | 5.71E-02 | 1.27E-02 | 4.505 | 6.63E-06 | 0.0007507 | lncRNA |  |  |
| 160 | ENSG00000113805 | 1049.58 | 3.98E-02 | 8.85E-03 | 4.502 | 6.73E-06 | 0.00075769 | protein_coding | CNTN3 | 5067 |
| 161 | ENSG00000180332 | 336.05 | 6.85E-02 | 1.52E-02 | 4.496 | 6.93E-06 | 0.0007756 | protein_coding | KCTD4 | 386618 |
| 162 | ENSG00000198133 | 141.39 | 3.49E-02 | 7.78E-03 | 4.489 | 7.16E-06 | 0.00079423 | protein_coding | TMEM229B | 161145 |
| 163 | ENSG00000254798 | 18.26 | 5.43E-02 | 1.21E-02 | 4.489 | 7.17E-06 | 0.00079423 | processed_pseudogene |  |  |
| 164 | ENSG00000174238 | 9576.45 | 5.20E-02 | 1.16E-02 | 4.482 | 7.40E-06 | 0.00081473 | protein_coding | PITPNA | 5306 |
| 165 | ENSG00000091428 | 3078.26 | 3.95E-02 | 8.81E-03 | 4.481 | 7.44E-06 | 0.00081502 | protein_coding | RAPGEF4 | 11069 |
| 166 | ENSG00000178445 | 992.61 | 3.70E-02 | 8.26E-03 | 4.479 | 7.49E-06 | 0.00081637 | protein_coding | GLDC | 2731 |
| 167 | ENSG00000165300 | 42.92 | -4.57E-02 | 1.02E-02 | -4.474 | 7.68E-06 | 0.00083175 | protein_coding | SLITRK5 | 26050 |
| 168 | ENSG00000151320 | 2388.41 | 4.61E-02 | 1.03E-02 | 4.472 | 7.74E-06 | 0.00083435 | protein_coding | AKAP6 | 9472 |
| 169 | ENSG00000140526 | 8177.34 | 4.57E-02 | 1.02E-02 | 4.467 | 7.93E-06 | 0.00084961 | protein_coding | ABHD2 | 11057 |
| 170 | ENSG00000167757 | 32.94 | 7.27E-02 | 1.63E-02 | 4.463 | 8.09E-06 | 0.00086314 | protein_coding | KLK11 | 11012 |
| 171 | ENSG00000188783 | 2278.56 | -3.82E-02 | 8.57E-03 | -4.461 | 8.18E-06 | 0.00086731 | protein_coding | PRELP | 5549 |
| 172 | ENSG00000187783 | 653.71 | 3.85E-02 | 8.63E-03 | 4.459 | 8.23E-06 | 0.00086805 | protein_coding | TMEM72 | 643236 |
| 173 | ENSG00000163545 | 182.12 | 4.63E-02 | 1.04E-02 | 4.457 | 8.32E-06 | 0.00087381 | protein_coding | NUAK2 | 81788 |
| 174 | ENSG00000129993 | 20.94 | -4.60E-02 | 1.03E-02 | -4.454 | 8.44E-06 | 0.0008813 | protein_coding | CBFA2T3 | 863 |
| 175 | ENSG00000162782 | 56.79 | 3.45E-02 | 7.76E-03 | 4.451 | 8.56E-06 | 0.00088886 | protein_coding | TDRD5 | 163589 |
| 176 | ENSG00000187889 | 1142.61 | 3.42E-02 | 7.71E-03 | 4.440 | 8.99E-06 | 0.00092871 | protein_coding | FYB2 | 199920 |
| 177 | ENSG00000198756 | 397.94 | -3.32E-02 | 7.47E-03 | -4.439 | 9.05E-06 | 0.00093033 | protein_coding | COLGALT2 | 23127 |
| 178 | ENSG00000170745 | 473.85 | 4.31E-02 | 9.71E-03 | 4.436 | 9.16E-06 | 0.00093662 | protein_coding | KCNS3 | 3790 |
| 179 | ENSG00000225940 | 91.71 | 4.90E-02 | 1.11E-02 | 4.424 | 9.67E-06 | 0.00098396 | lncRNA |  |  |
| 180 | ENSG00000251859 | 23.83 | 6.02E-02 | 1.36E-02 | 4.423 | 9.72E-06 | 0.00098396 | snRNA | RNU6-1288P | 106480128 |
| 181 | ENSG00000060709 | 94.36 | -5.11E-02 | 1.16E-02 | -4.420 | 9.86E-06 | 0.0009931 | protein_coding | RIMBP2 | 23504 |
| 182 | ENSG00000073792 | 248.45 | -3.65E-02 | 8.29E-03 | -4.397 | 1.10E-05 | 0.00109189 | protein_coding | IGF2BP2 | 10644 |
| 183 | ENSG00000142611 | 942.55 | 3.04E-02 | 6.92E-03 | 4.397 | 1.10E-05 | 0.00109189 | protein_coding | PRDM16 | 63976 |
| 184 | ENSG00000164056 | 488.39 | 2.63E-02 | 5.99E-03 | 4.393 | 1.12E-05 | 0.0011021 | protein_coding | SPRY1 | 10252 |
| 185 | ENSG00000188937 | 68.25 | 4.94E-02 | 1.13E-02 | 4.392 | 1.12E-05 | 0.0011039 | protein_coding | NYX | 60506 |
| 186 | ENSG00000225746 | 83.13 | -3.21E-02 | 7.31E-03 | -4.389 | 1.14E-05 | 0.00111565 | lncRNA |  |  |
| 187 | ENSG00000147003 | 1690.89 | 5.15E-02 | 1.17E-02 | 4.387 | 1.15E-05 | 0.0011175 | protein_coding | CLTRN | 57393 |
| 188 | ENSG00000156097 | 298.43 | 3.64E-02 | 8.30E-03 | 4.381 | 1.18E-05 | 0.00114344 | protein_coding | GPR61 | 83873 |
| 189 | ENSG00000156925 | 57.82 | -5.83E-02 | 1.33E-02 | -4.379 | 1.19E-05 | 0.00114851 | protein_coding | ZIC3 | 7547 |
| 190 | ENSG00000112297 | 4337.08 | 3.37E-02 | 7.71E-03 | 4.374 | 1.22E-05 | 0.00116944 | protein_coding | CRYBG1 | 202 |
| 191 | ENSG00000131941 | 110.27 | -2.86E-02 | 6.54E-03 | -4.369 | 1.25E-05 | 0.00118825 | protein_coding | RHPN2 | 85415 |
| 192 | ENSG00000107742 | 239.75 | -3.82E-02 | 8.75E-03 | -4.368 | 1.26E-05 | 0.00118825 | protein_coding | SPOCK2 | 9806 |
| 193 | ENSG00000196155 | 57.66 | 3.03E-02 | 6.95E-03 | 4.359 | 1.31E-05 | 0.00122635 | protein_coding | PLEKHG4 | 25894 |
| 194 | ENSG00000243440 | 223.93 | 6.54E-02 | 1.50E-02 | 4.353 | 1.34E-05 | 0.00125423 | NA |  |  |
| 195 | ENSG00000116039 | 46.66 | -6.58E-02 | 1.51E-02 | -4.343 | 1.41E-05 | 0.00130824 | protein_coding | ATP6V1B1 | 525 |
| 196 | ENSG00000179136 | 47.75 | 5.24E-02 | 1.21E-02 | 4.339 | 1.43E-05 | 0.00132172 | lncRNA | LINC00670 | 284034 |
| 197 | ENSG00000068078 | 250.78 | 5.25E-02 | 1.21E-02 | 4.335 | 1.46E-05 | 0.00134236 | protein_coding | FGFR3 | 2261 |
| 198 | ENSG00000006118 | 609.90 | 3.67E-02 | 8.48E-03 | 4.334 | 1.47E-05 | 0.00134236 | protein_coding | TMEM132A | 54972 |
| 199 | ENSG00000102468 | 287.81 | -4.65E-02 | 1.07E-02 | -4.331 | 1.48E-05 | 0.0013536 | protein_coding | HTR2A | 3356 |
| 200 | ENSG00000113924 | 442.46 | 2.81E-02 | 6.50E-03 | 4.322 | 1.55E-05 | 0.0014061 | protein_coding | HGD | 3081 |
| 201 | ENSG00000135063 | 242.86 | 2.69E-02 | 6.23E-03 | 4.317 | 1.58E-05 | 0.00142141 | protein_coding | FAM189A2 | 9413 |
| 202 | ENSG00000100592 | 6020.14 | 3.27E-02 | 7.57E-03 | 4.316 | 1.59E-05 | 0.00142141 | protein_coding | DAAM1 | 23002 |
| 203 | ENSG00000160229 | 155.58 | 3.59E-02 | 8.31E-03 | 4.315 | 1.59E-05 | 0.00142141 | protein_coding | ZNF66 | 7617 |
| 204 | ENSG00000062038 | 2491.42 | 5.32E-02 | 1.23E-02 | 4.315 | 1.59E-05 | 0.00142141 | protein_coding | CDH3 | 1001 |
| 205 | ENSG00000173805 | 6.92 | -6.41E-02 | 1.49E-02 | -4.310 | 1.63E-05 | 0.00144712 | protein_coding | HAP1 | 9001 |
| 206 | ENSG00000249242 | 242.98 | -3.07E-02 | 7.12E-03 | -4.306 | 1.66E-05 | 0.0014711 | protein_coding | TMEM150C | 441027 |
| 207 | ENSG00000134824 | 1674.58 | 3.57E-02 | 8.30E-03 | 4.304 | 1.68E-05 | 0.00147889 | protein_coding | FADS2 | 9415 |
| 208 | ENSG00000149150 | 134.13 | -4.20E-02 | 9.77E-03 | -4.300 | 1.71E-05 | 0.00149959 | protein_coding | SLC43A1 | 8501 |
| 209 | ENSG00000135912 | 1005.44 | 3.49E-02 | 8.13E-03 | 4.296 | 1.74E-05 | 0.00151625 | protein_coding | TTLL4 | 9654 |
| 210 | ENSG00000248590 | 190.11 | 3.62E-02 | 8.44E-03 | 4.293 | 1.76E-05 | 0.00152804 | transcribed_processed_pseudogene |  |  |
| 211 | ENSG00000166501 | 1630.50 | 3.33E-02 | 7.75E-03 | 4.291 | 1.78E-05 | 0.00153686 | protein_coding | PRKCB | 5579 |
| 212 | ENSG00000116667 | 1562.16 | -2.91E-02 | 6.78E-03 | -4.290 | 1.78E-05 | 0.00153686 | protein_coding | C1orf21 | 81563 |
| 213 | ENSG00000114646 | 1144.58 | 5.09E-02 | 1.19E-02 | 4.284 | 1.83E-05 | 0.00157184 | protein_coding | CSPG5 | 10675 |
| 214 | ENSG00000232803 | 58.30 | -3.61E-02 | 8.43E-03 | -4.281 | 1.86E-05 | 0.00158926 | lncRNA | SLCO4A1-AS1 | 100127888 |
| 215 | ENSG00000185532 | 382.20 | -3.38E-02 | 7.90E-03 | -4.279 | 1.87E-05 | 0.00159324 | protein_coding | PRKG1 | 5592 |
| 216 | ENSG00000249306 | 33.09 | 5.92E-02 | 1.38E-02 | 4.278 | 1.88E-05 | 0.00159382 | lncRNA | LINC01411 | 101928176 |
| 217 | ENSG00000126259 | 65.68 | 7.00E-02 | 1.64E-02 | 4.277 | 1.89E-05 | 0.00159382 | protein_coding | KIRREL2 | 84063 |
| 218 | ENSG00000170439 | 88.53 | 4.33E-02 | 1.02E-02 | 4.269 | 1.96E-05 | 0.00164979 | protein_coding | METTL7B | 196410 |
| 219 | ENSG00000152495 | 87.28 | -4.35E-02 | 1.02E-02 | -4.265 | 2.00E-05 | 0.00166429 | protein_coding | CAMK4 | 814 |
| 220 | ENSG00000125869 | 35.18 | -4.10E-02 | 9.62E-03 | -4.265 | 2.00E-05 | 0.00166429 | protein_coding | LAMP5 | 24141 |
| 221 | ENSG00000162073 | 456.73 | 4.65E-02 | 1.09E-02 | 4.264 | 2.01E-05 | 0.00166898 | protein_coding | PAQR4 | 124222 |
| 222 | ENSG00000111371 | 1480.76 | -4.23E-02 | 9.91E-03 | -4.262 | 2.03E-05 | 0.001674 | protein_coding | SLC38A1 | 81539 |
| 223 | ENSG00000153790 | 411.51 | 5.14E-02 | 1.21E-02 | 4.260 | 2.05E-05 | 0.00168022 | protein_coding | C7orf31 | 136895 |
| 224 | ENSG00000256391 | 40.70 | -7.50E-02 | 1.76E-02 | -4.259 | 2.05E-05 | 0.00168022 | NA |  |  |
| 225 | ENSG00000259264 | 216.09 | 3.68E-02 | 8.66E-03 | 4.251 | 2.13E-05 | 0.00173179 | NA |  |  |
| 226 | ENSG00000186439 | 242.72 | -3.64E-02 | 8.56E-03 | -4.249 | 2.14E-05 | 0.0017349 | protein_coding | TRDN | 10345 |
| 227 | ENSG00000110675 | 92.10 | -5.00E-02 | 1.18E-02 | -4.246 | 2.17E-05 | 0.00175112 | protein_coding | ELMOD1 | 55531 |
| 228 | ENSG00000117598 | 44.48 | -6.27E-02 | 1.48E-02 | -4.243 | 2.21E-05 | 0.00176764 | protein_coding | PLPPR5 | 163404 |
| 229 | ENSG00000150656 | 1337.78 | 6.24E-02 | 1.47E-02 | 4.243 | 2.21E-05 | 0.00176764 | protein_coding | CNDP1 | 84735 |
| 230 | ENSG00000168671 | 114.61 | 6.41E-02 | 1.51E-02 | 4.240 | 2.23E-05 | 0.00177818 | protein_coding | UGT3A2 | 167127 |
| 231 | ENSG00000182985 | 2643.79 | -4.02E-02 | 9.49E-03 | -4.235 | 2.28E-05 | 0.00180986 | protein_coding | CADM1 | 23705 |
| 232 | ENSG00000198785 | 24.25 | -4.92E-02 | 1.16E-02 | -4.233 | 2.31E-05 | 0.00181109 | protein_coding | GRIN3A | 116443 |
| 233 | ENSG00000116990 | 73.30 | 5.02E-02 | 1.19E-02 | 4.230 | 2.34E-05 | 0.00182734 | protein_coding | MYCL | 4610 |
| 234 | ENSG00000256956 | 22.57 | -6.36E-02 | 1.50E-02 | -4.229 | 2.35E-05 | 0.00182951 | NA |  |  |
| 235 | ENSG00000224593 | 8.46 | -6.59E-02 | 1.56E-02 | -4.225 | 2.39E-05 | 0.00185598 | transcribed_processed_pseudogene | RPL21P72 | 100271181 |
| 236 | ENSG00000116791 | 1525.01 | 3.05E-02 | 7.23E-03 | 4.218 | 2.47E-05 | 0.00190499 | protein_coding | CRYZ | 1429 |
| 237 | ENSG00000091986 | 2795.27 | 3.24E-02 | 7.67E-03 | 4.217 | 2.48E-05 | 0.00190825 | protein_coding | CCDC80 | 151887 |
| 238 | ENSG00000163331 | 677.41 | -3.14E-02 | 7.45E-03 | -4.213 | 2.52E-05 | 0.00192114 | protein_coding | DAPL1 | 92196 |
| 239 | ENSG00000255746 | 325.69 | 3.12E-02 | 7.40E-03 | 4.212 | 2.53E-05 | 0.0019259 | lncRNA | LOC102723544 | 102723544 |
| 240 | ENSG00000057704 | 1040.33 | 2.59E-02 | 6.15E-03 | 4.207 | 2.59E-05 | 0.00196494 | protein_coding | TMCC3 | 57458 |
| 241 | ENSG00000188687 | 4316.70 | 5.06E-02 | 1.20E-02 | 4.204 | 2.63E-05 | 0.00196906 | protein_coding | SLC4A5 | 57835 |
| 242 | ENSG00000159399 | 927.48 | -5.48E-02 | 1.30E-02 | -4.201 | 2.65E-05 | 0.00197421 | protein_coding | HK2 | 3099 |
| 243 | ENSG00000198719 | 144.02 | -2.56E-02 | 6.10E-03 | -4.195 | 2.73E-05 | 0.00201861 | protein_coding | DLL1 | 28514 |
| 244 | ENSG00000169436 | 12.35 | -7.16E-02 | 1.71E-02 | -4.190 | 2.79E-05 | 0.00205308 | protein_coding | COL22A1 | 169044 |
| 245 | ENSG00000268892 | 131.44 | 6.73E-02 | 1.61E-02 | 4.185 | 2.85E-05 | 0.00208788 | unprocessed_pseudogene |  |  |
| 246 | ENSG00000099954 | 181.66 | -3.96E-02 | 9.48E-03 | -4.183 | 2.87E-05 | 0.00209713 | protein_coding | CECR2 | 27443 |
| 247 | ENSG00000135476 | 55.60 | 2.80E-02 | 6.69E-03 | 4.182 | 2.89E-05 | 0.00209713 | protein_coding | ESPL1 | 9700 |
| 248 | ENSG00000197565 | 125.23 | -4.49E-02 | 1.07E-02 | -4.182 | 2.89E-05 | 0.00209713 | protein_coding | COL4A6 | 1288 |
| 249 | ENSG00000119655 | 1997.35 | 2.86E-02 | 6.83E-03 | 4.180 | 2.91E-05 | 0.00210566 | protein_coding | NPC2 | 10577 |
| 250 | ENSG00000165078 | 86.53 | -4.33E-02 | 1.04E-02 | -4.177 | 2.95E-05 | 0.00212264 | protein_coding | CPA6 | 57094 |
| 251 | ENSG00000171130 | 557.08 | 3.32E-02 | 7.96E-03 | 4.166 | 3.10E-05 | 0.00222069 | protein_coding | ATP6V0E2 | 155066 |
| 252 | ENSG00000181218 | 267.54 | 2.95E-02 | 7.09E-03 | 4.161 | 3.18E-05 | 0.00226938 | protein_coding | H2AW | 92815 |
| 253 | ENSG00000198088 | 24.84 | 3.27E-02 | 7.86E-03 | 4.156 | 3.24E-05 | 0.00229823 | protein_coding | NUP62CL | 54830 |
| 254 | ENSG00000144229 | 624.22 | -2.67E-02 | 6.42E-03 | -4.156 | 3.24E-05 | 0.00229823 | protein_coding | THSD7B | 80731 |
| 255 | ENSG00000228540 | 35.76 | 3.90E-02 | 9.39E-03 | 4.155 | 3.26E-05 | 0.00230521 | lncRNA |  |  |
| 256 | ENSG00000259235 | 33.43 | 3.50E-02 | 8.42E-03 | 4.153 | 3.28E-05 | 0.00231234 | lncRNA |  |  |
| 257 | ENSG00000162630 | 82.27 | -6.58E-02 | 1.59E-02 | -4.150 | 3.32E-05 | 0.00233438 | protein_coding | B3GALT2 | 8707 |
| 258 | ENSG00000183145 | 15.69 | 5.08E-02 | 1.23E-02 | 4.144 | 3.41E-05 | 0.00238141 | protein_coding | RIPPLY3 | 53820 |
| 259 | ENSG00000185008 | 949.83 | 4.97E-02 | 1.20E-02 | 4.140 | 3.47E-05 | 0.00241007 | protein_coding | ROBO2 | 6092 |
| 260 | ENSG00000135414 | 1747.21 | 4.51E-02 | 1.09E-02 | 4.136 | 3.53E-05 | 0.00244329 | protein_coding | GDF11 | 10220 |
| 261 | ENSG00000225127 | 16.17 | 7.03E-02 | 1.70E-02 | 4.132 | 3.60E-05 | 0.00247592 | lncRNA | LINC00237 | 105372556 |
| 262 | ENSG00000254115 | 41.52 | 4.55E-02 | 1.10E-02 | 4.131 | 3.61E-05 | 0.00247592 | lncRNA |  |  |
| 263 | ENSG00000111863 | 4649.24 | 3.70E-02 | 8.95E-03 | 4.128 | 3.66E-05 | 0.00250111 | protein_coding | ADTRP | 84830 |
| 264 | ENSG00000116183 | 800.92 | -7.94E-02 | 1.92E-02 | -4.124 | 3.72E-05 | 0.00252341 | protein_coding | PAPPA2 | 60676 |
| 265 | ENSG00000183023 | 1144.37 | -3.55E-02 | 8.60E-03 | -4.124 | 3.72E-05 | 0.00252341 | protein_coding | SLC8A1 | 6546 |
| 266 | ENSG00000228063 | 18.32 | 3.81E-02 | 9.25E-03 | 4.123 | 3.74E-05 | 0.00252898 | lncRNA | LYPLAL1-DT | 643723 |
| 267 | ENSG00000164292 | 8291.85 | 2.87E-02 | 6.97E-03 | 4.119 | 3.81E-05 | 0.00256561 | protein_coding | RHOBTB3 | 22836 |
| 268 | ENSG00000132386 | 62805.48 | 3.41E-02 | 8.27E-03 | 4.117 | 3.84E-05 | 0.00257383 | protein_coding | SERPINF1 | 5176 |
| 269 | ENSG00000213976 | 42.75 | 3.69E-02 | 8.95E-03 | 4.117 | 3.84E-05 | 0.00257383 | unprocessed_pseudogene |  |  |
| 270 | ENSG00000145808 | 175.82 | 3.26E-02 | 7.93E-03 | 4.113 | 3.90E-05 | 0.00259341 | protein_coding | ADAMTS19 | 171019 |
| 271 | ENSG00000118322 | 2209.10 | 5.13E-02 | 1.25E-02 | 4.107 | 4.01E-05 | 0.00265458 | protein_coding | ATP10B | 23120 |
| 272 | ENSG00000172164 | 1364.39 | 4.04E-02 | 9.84E-03 | 4.106 | 4.02E-05 | 0.00265458 | protein_coding | SNTB1 | 6641 |
| 273 | ENSG00000162595 | 87.94 | -2.99E-02 | 7.28E-03 | -4.104 | 4.06E-05 | 0.00267691 | protein_coding | DIRAS3 | 9077 |
| 274 | ENSG00000139155 | 1424.06 | 5.81E-02 | 1.42E-02 | 4.101 | 4.12E-05 | 0.00270256 | protein_coding | SLCO1C1 | 53919 |
| 275 | ENSG00000178662 | 2335.35 | 3.56E-02 | 8.69E-03 | 4.100 | 4.14E-05 | 0.00270791 | protein_coding | CSRNP3 | 80034 |
| 276 | ENSG00000136541 | 7236.36 | 7.59E-02 | 1.85E-02 | 4.096 | 4.21E-05 | 0.00273676 | protein_coding | ERMN | 57471 |
| 277 | ENSG00000043355 | 46.21 | -6.65E-02 | 1.62E-02 | -4.096 | 4.21E-05 | 0.00273676 | protein_coding | ZIC2 | 7546 |
| 278 | ENSG00000130876 | 63.69 | 5.40E-02 | 1.32E-02 | 4.091 | 4.29E-05 | 0.00277222 | protein_coding | SLC7A10 | 56301 |
| 279 | ENSG00000165548 | 171.85 | 4.02E-02 | 9.84E-03 | 4.090 | 4.31E-05 | 0.00277437 | protein_coding | TMEM63C | 57156 |
| 280 | ENSG00000182747 | 105.06 | -4.92E-02 | 1.20E-02 | -4.088 | 4.36E-05 | 0.00279822 | protein_coding | SLC35D3 | 340146 |
| 281 | ENSG00000117152 | 172.80 | -4.96E-02 | 1.21E-02 | -4.085 | 4.40E-05 | 0.00279822 | protein_coding | RGS4 | 5999 |
| 282 | ENSG00000223414 | 206.15 | -6.00E-02 | 1.47E-02 | -4.085 | 4.41E-05 | 0.00279822 | NA |  |  |
| 283 | ENSG00000143479 | 146.25 | -2.76E-02 | 6.75E-03 | -4.085 | 4.41E-05 | 0.00279822 | protein_coding | DYRK3 | 8444 |
| 284 | ENSG00000180720 | 44.67 | -4.74E-02 | 1.16E-02 | -4.085 | 4.42E-05 | 0.00279822 | protein_coding | CHRM4 | 1132 |
| 285 | ENSG00000258998 | 152.11 | 5.36E-02 | 1.31E-02 | 4.080 | 4.51E-05 | 0.00284046 | lncRNA |  |  |
| 286 | ENSG00000180914 | 120.96 | -8.93E-02 | 2.19E-02 | -4.069 | 4.71E-05 | 0.00294957 | protein_coding | OXTR | 5021 |
| 287 | ENSG00000175155 | 721.12 | -2.65E-02 | 6.53E-03 | -4.064 | 4.82E-05 | 0.00301006 | protein_coding | YPEL2 | 388403 |
| 288 | ENSG00000165606 | 6.55 | 8.26E-02 | 2.03E-02 | 4.063 | 4.84E-05 | 0.00301018 | protein_coding | DRGX | 644168 |
| 289 | ENSG00000119888 | 45.95 | -5.31E-02 | 1.31E-02 | -4.060 | 4.91E-05 | 0.00304617 | protein_coding | EPCAM | 4072 |
| 290 | ENSG00000105699 | 471.92 | 3.73E-02 | 9.20E-03 | 4.058 | 4.95E-05 | 0.00305792 | protein_coding | LSR | 51599 |
| 291 | ENSG00000073605 | 45.09 | -3.09E-02 | 7.62E-03 | -4.057 | 4.97E-05 | 0.00306416 | protein_coding | GSDMB | 55876 |
| 292 | ENSG00000170044 | 40.43 | -3.35E-02 | 8.27E-03 | -4.054 | 5.04E-05 | 0.00309687 | protein_coding | ZPLD1 | 131368 |
| 293 | ENSG00000165092 | 1683.38 | -3.35E-02 | 8.26E-03 | -4.049 | 5.14E-05 | 0.00314895 | protein_coding | ALDH1A1 | 216 |
| 294 | ENSG00000255371 | 19.05 | 5.47E-02 | 1.35E-02 | 4.045 | 5.24E-05 | 0.00320057 | lncRNA |  |  |
| 295 | ENSG00000163694 | 2123.89 | 2.99E-02 | 7.39E-03 | 4.042 | 5.30E-05 | 0.00322662 | protein_coding | RBM47 | 54502 |
| 296 | ENSG00000199400 | 39.11 | 6.50E-02 | 1.61E-02 | 4.040 | 5.35E-05 | 0.00324735 | misc_RNA | RNY4P19 | 100169834 |
| 297 | ENSG00000183831 | 38.44 | -3.68E-02 | 9.11E-03 | -4.036 | 5.43E-05 | 0.00327541 | protein_coding | ANKRD45 | 339416 |
| 298 | ENSG00000188517 | 753.52 | -3.49E-02 | 8.66E-03 | -4.033 | 5.50E-05 | 0.00331079 | protein_coding | COL25A1 | 84570 |
| 299 | ENSG00000125462 | 217.47 | 3.92E-02 | 9.72E-03 | 4.031 | 5.54E-05 | 0.00332466 | lncRNA | MIR9-1HG | 10485 |
| 300 | ENSG00000137261 | 29.47 | -6.03E-02 | 1.50E-02 | -4.026 | 5.68E-05 | 0.00339809 | protein_coding | KIAA0319 | 9856 |
| 301 | ENSG00000004864 | 1088.76 | 2.74E-02 | 6.81E-03 | 4.024 | 5.72E-05 | 0.00340849 | protein_coding | SLC25A13 | 10165 |
| 302 | ENSG00000127586 | 28.09 | 2.87E-02 | 7.14E-03 | 4.023 | 5.73E-05 | 0.00340849 | protein_coding | CHTF18 | 63922 |
| 303 | ENSG00000163029 | 3860.31 | 3.46E-02 | 8.60E-03 | 4.022 | 5.76E-05 | 0.00340849 | protein_coding | SMC6 | 79677 |
| 304 | ENSG00000152910 | 18.14 | -9.96E-02 | 2.48E-02 | -4.022 | 5.77E-05 | 0.00340849 | protein_coding | CNTNAP4 | 85445 |
| 305 | ENSG00000130427 | 17.03 | 5.70E-02 | 1.42E-02 | 4.021 | 5.80E-05 | 0.00341797 | protein_coding | EPO | 2056 |
| 306 | ENSG00000220377 | 239.74 | 9.96E-02 | 2.48E-02 | 4.016 | 5.91E-05 | 0.0034717 | unprocessed_pseudogene |  |  |
| 307 | ENSG00000269936 | 11.36 | -7.94E-02 | 1.98E-02 | -4.013 | 5.99E-05 | 0.00349656 | NA |  |  |
| 308 | ENSG00000197587 | 15.24 | 7.33E-02 | 1.83E-02 | 4.009 | 6.10E-05 | 0.00353655 | protein_coding | DMBX1 | 127343 |
| 309 | ENSG00000161509 | 5.13 | -8.76E-02 | 2.18E-02 | -4.009 | 6.11E-05 | 0.00353655 | protein_coding | GRIN2C | 2905 |
| 310 | ENSG00000151834 | 27.78 | -3.54E-02 | 8.83E-03 | -4.008 | 6.13E-05 | 0.00353655 | protein_coding | GABRA2 | 2555 |
| 311 | ENSG00000106066 | 2483.04 | 3.40E-02 | 8.48E-03 | 4.007 | 6.14E-05 | 0.00353655 | protein_coding | CPVL | 54504 |
| 312 | ENSG00000186481 | 1050.75 | 5.02E-02 | 1.25E-02 | 4.005 | 6.21E-05 | 0.00356012 | transcribed_unprocessed_pseudogene |  |  |
| 313 | ENSG00000103710 | 296.05 | 5.50E-02 | 1.37E-02 | 4.004 | 6.22E-05 | 0.00356012 | protein_coding | RASL12 | 51285 |
| 314 | ENSG00000170011 | 7979.78 | 3.98E-02 | 9.96E-03 | 4.000 | 6.34E-05 | 0.00361839 | protein_coding | MYRIP | 25924 |
| 315 | ENSG00000262061 | 17.00 | -6.60E-02 | 1.65E-02 | -3.995 | 6.46E-05 | 0.00367704 | lncRNA |  |  |
| 316 | ENSG00000174469 | 226.25 | -6.35E-02 | 1.59E-02 | -3.992 | 6.54E-05 | 0.00370338 | protein_coding | CNTNAP2 | 26047 |
| 317 | ENSG00000163406 | 1012.34 | -4.06E-02 | 1.02E-02 | -3.990 | 6.61E-05 | 0.00373152 | protein_coding | SLC15A2 | 6565 |
| 318 | ENSG00000116745 | 63520.57 | 8.06E-02 | 2.02E-02 | 3.985 | 6.74E-05 | 0.00378014 | protein_coding | RPE65 | 6121 |
| 319 | ENSG00000114200 | 33.69 | -4.28E-02 | 1.07E-02 | -3.985 | 6.75E-05 | 0.00378014 | protein_coding | BCHE | 590 |
| 320 | ENSG00000164626 | 11.43 | 5.75E-02 | 1.45E-02 | 3.975 | 7.05E-05 | 0.00393705 | protein_coding | KCNK5 | 8645 |
| 321 | ENSG00000183196 | 21.04 | -4.69E-02 | 1.18E-02 | -3.973 | 7.09E-05 | 0.00393891 | protein_coding | CHST6 | 4166 |
| 322 | ENSG00000159166 | 19.04 | 6.13E-02 | 1.54E-02 | 3.973 | 7.09E-05 | 0.00393891 | protein_coding | LAD1 | 3898 |
| 323 | ENSG00000216775 | 1257.53 | 7.64E-02 | 1.93E-02 | 3.967 | 7.27E-05 | 0.00401332 | transcribed_unprocessed_pseudogene | LOC730101 | 730101 |
| 324 | ENSG00000198753 | 73.22 | 4.56E-02 | 1.15E-02 | 3.965 | 7.35E-05 | 0.00403141 | protein_coding | PLXNB3 | 5365 |
| 325 | ENSG00000155897 | 17.57 | -7.62E-02 | 1.92E-02 | -3.964 | 7.37E-05 | 0.00403141 | protein_coding | ADCY8 | 114 |
| 326 | ENSG00000132164 | 16.61 | -1.02E-01 | 2.57E-02 | -3.963 | 7.39E-05 | 0.00403141 | protein_coding | SLC6A11 | 6538 |
| 327 | ENSG00000167371 | 27.28 | -3.63E-02 | 9.16E-03 | -3.963 | 7.40E-05 | 0.00403141 | protein_coding | PRRT2 | 112476 |
| 328 | ENSG00000200753 | 23.63 | 4.43E-02 | 1.12E-02 | 3.962 | 7.42E-05 | 0.00403141 | snoRNA |  |  |
| 329 | ENSG00000168672 | 3835.14 | 3.15E-02 | 7.95E-03 | 3.959 | 7.53E-05 | 0.00406587 | protein_coding | LRATD2 | 157638 |
| 330 | ENSG00000178295 | 1191.44 | 4.76E-02 | 1.20E-02 | 3.958 | 7.55E-05 | 0.00406587 | protein_coding | GEN1 | 348654 |
| 331 | ENSG00000144810 | 6418.04 | 3.21E-02 | 8.11E-03 | 3.957 | 7.59E-05 | 0.00407777 | protein_coding | COL8A1 | 1295 |
| 332 | ENSG00000112902 | 780.11 | -3.01E-02 | 7.60E-03 | -3.956 | 7.61E-05 | 0.00407777 | protein_coding | SEMA5A | 9037 |
| 333 | ENSG00000112333 | 360.34 | 4.70E-02 | 1.19E-02 | 3.956 | 7.63E-05 | 0.00407843 | protein_coding | NR2E1 | 7101 |
| 334 | ENSG00000237943 | 128.12 | 5.10E-02 | 1.29E-02 | 3.951 | 7.78E-05 | 0.00413471 | lncRNA | PRKCQ-AS1 | 439949 |
| 335 | ENSG00000156414 | 1304.44 | 3.36E-02 | 8.51E-03 | 3.951 | 7.80E-05 | 0.00413471 | protein_coding | TDRD9 | 122402 |
| 336 | ENSG00000235027 | 44.29 | 3.85E-02 | 9.75E-03 | 3.950 | 7.83E-05 | 0.00414061 | lncRNA |  |  |
| 337 | ENSG00000198944 | 190.17 | 3.63E-02 | 9.20E-03 | 3.946 | 7.94E-05 | 0.00418072 | protein_coding | SOWAHA | 134548 |
| 338 | ENSG00000182916 | 104.19 | -3.01E-02 | 7.65E-03 | -3.932 | 8.41E-05 | 0.00440353 | protein_coding | TCEAL7 | 56849 |
| 339 | ENSG00000231992 | 128.05 | 3.64E-02 | 9.26E-03 | 3.931 | 8.46E-05 | 0.00441761 | lncRNA |  |  |
| 340 | ENSG00000101439 | 7864.84 | 4.33E-02 | 1.10E-02 | 3.930 | 8.48E-05 | 0.00441809 | protein_coding | CST3 | 1471 |
| 341 | ENSG00000136267 | 28.83 | -8.16E-02 | 2.08E-02 | -3.929 | 8.54E-05 | 0.00443811 | protein_coding | DGKB | 1607 |
| 342 | ENSG00000182938 | 18.40 | 6.13E-02 | 1.56E-02 | 3.923 | 8.75E-05 | 0.00453288 | protein_coding | OTOP3 | 347741 |
| 343 | ENSG00000184254 | 8027.31 | 3.51E-02 | 8.95E-03 | 3.918 | 8.92E-05 | 0.0046089 | protein_coding | ALDH1A3 | 220 |
| 344 | ENSG00000187258 | 14.41 | -7.40E-02 | 1.89E-02 | -3.918 | 8.94E-05 | 0.0046089 | protein_coding | NPSR1 | 387129 |
| 345 | ENSG00000258487 | 15.97 | 5.06E-02 | 1.29E-02 | 3.914 | 9.08E-05 | 0.00466881 | lncRNA |  |  |
| 346 | ENSG00000184302 | 31.92 | -6.22E-02 | 1.59E-02 | -3.913 | 9.12E-05 | 0.00466994 | protein_coding | SIX6 | 4990 |
| 347 | ENSG00000133069 | 541.03 | 2.97E-02 | 7.59E-03 | 3.913 | 9.13E-05 | 0.00466994 | protein_coding | TMCC2 | 9911 |
| 348 | ENSG00000145284 | 1034.68 | -3.08E-02 | 7.87E-03 | -3.910 | 9.24E-05 | 0.0047006 | protein_coding | SCD5 | 79966 |
| 349 | ENSG00000146904 | 47.77 | 4.61E-02 | 1.18E-02 | 3.910 | 9.24E-05 | 0.0047006 | protein_coding | EPHA1 | 2041 |
| 350 | ENSG00000104205 | 1165.83 | 4.29E-02 | 1.10E-02 | 3.906 | 9.40E-05 | 0.00477201 | protein_coding | SGK3 | 23678 |
| 351 | ENSG00000165478 | 65.17 | 7.31E-02 | 1.88E-02 | 3.898 | 9.71E-05 | 0.00491828 | protein_coding | HEPACAM | 220296 |
| 352 | ENSG00000179178 | 17.70 | 6.17E-02 | 1.58E-02 | 3.897 | 9.75E-05 | 0.00492349 | protein_coding | TMEM125 | 128218 |
| 353 | ENSG00000169933 | 256.79 | 4.74E-02 | 1.22E-02 | 3.896 | 9.80E-05 | 0.00493718 | protein_coding | FRMPD4 | 9758 |
| 354 | ENSG00000141449 | 27.14 | -7.72E-02 | 1.98E-02 | -3.893 | 9.90E-05 | 0.00497436 | protein_coding | GREB1L | 80000 |
| 355 | ENSG00000050767 | 22.47 | -6.80E-02 | 1.75E-02 | -3.892 | 9.95E-05 | 0.00498955 | protein_coding | COL23A1 | 91522 |
| 356 | ENSG00000172687 | 187.43 | 3.15E-02 | 8.10E-03 | 3.888 | 0.00010087 | 0.00504466 | protein_coding | ZNF738 | 148203 |
| 357 | ENSG00000081138 | 22.02 | -7.11E-02 | 1.83E-02 | -3.884 | 0.00010286 | 0.00511855 | protein_coding | CDH7 | 1005 |
| 358 | ENSG00000161649 | 15.16 | -5.08E-02 | 1.31E-02 | -3.872 | 0.00010801 | 0.00533546 | protein_coding | CD300LG | 146894 |
| 359 | ENSG00000131094 | 19.08 | -5.36E-02 | 1.39E-02 | -3.865 | 0.00011104 | 0.00545693 | protein_coding | C1QL1 | 10882 |
| 360 | ENSG00000150471 | 165.96 | -4.09E-02 | 1.06E-02 | -3.865 | 0.00011125 | 0.00545693 | protein_coding | ADGRL3 | 23284 |
| 361 | ENSG00000180834 | 45.95 | 3.91E-02 | 1.01E-02 | 3.865 | 0.00011128 | 0.00545693 | protein_coding | MAP6D1 | 79929 |
| 362 | ENSG00000137285 | 77.02 | -4.06E-02 | 1.05E-02 | -3.863 | 0.00011209 | 0.00548316 | protein_coding | TUBB2B | 347733 |
| 363 | ENSG00000253864 | 23.32 | -6.50E-02 | 1.68E-02 | -3.861 | 0.00011292 | 0.00551035 | NA |  |  |
| 364 | ENSG00000178075 | 727.84 | 3.62E-02 | 9.39E-03 | 3.859 | 0.00011399 | 0.00554907 | protein_coding | GRAMD1C | 54762 |
| 365 | ENSG00000119686 | 1365.76 | 2.73E-02 | 7.08E-03 | 3.857 | 0.00011499 | 0.00557933 | protein_coding | FLVCR2 | 55640 |
| 366 | ENSG00000157423 | 55.25 | -4.48E-02 | 1.16E-02 | -3.856 | 0.00011516 | 0.00557933 | protein_coding | HYDIN | 54768 |
| 367 | ENSG00000118298 | 1356.42 | 3.95E-02 | 1.03E-02 | 3.849 | 0.00011839 | 0.00572197 | protein_coding | CA14 | 23632 |
| 368 | ENSG00000253309 | 515.03 | 8.71E-02 | 2.27E-02 | 3.845 | 0.00012072 | 0.00582065 | protein_coding | SERPINE3 | 647174 |
| 369 | ENSG00000164694 | 22.20 | 4.17E-02 | 1.09E-02 | 3.840 | 0.00012302 | 0.00589615 | protein_coding | FNDC1 | 84624 |
| 370 | ENSG00000139998 | 41.01 | -4.23E-02 | 1.10E-02 | -3.840 | 0.00012317 | 0.00589615 | protein_coding | RAB15 | 376267 |
| 371 | ENSG00000259439 | 749.22 | 3.09E-02 | 8.06E-03 | 3.838 | 0.0001242 | 0.00591138 | lncRNA | LINC01833 | 107985879 |
| 372 | ENSG00000154175 | 2181.65 | -2.93E-02 | 7.65E-03 | -3.838 | 0.00012424 | 0.00591138 | protein_coding | ABI3BP | 25890 |
| 373 | ENSG00000178234 | 3622.86 | 4.21E-02 | 1.10E-02 | 3.837 | 0.00012436 | 0.00591138 | protein_coding | GALNT11 | 63917 |
| 374 | ENSG00000156687 | 118.60 | -3.45E-02 | 9.00E-03 | -3.837 | 0.00012478 | 0.00591702 | protein_coding | UNC5D | 137970 |
| 375 | ENSG00000257139 | 9.60 | 1.11E-01 | 2.89E-02 | 3.836 | 0.00012507 | 0.00591702 | lncRNA |  |  |
| 376 | ENSG00000223392 | 28.23 | 5.90E-02 | 1.54E-02 | 3.835 | 0.00012569 | 0.00592216 | lncRNA | CLDN10-AS1 | 100874194 |
| 377 | ENSG00000185567 | 472.46 | -2.94E-02 | 7.67E-03 | -3.835 | 0.00012577 | 0.00592216 | protein_coding | AHNAK2 | 113146 |
| 378 | ENSG00000115252 | 362.92 | -3.50E-02 | 9.13E-03 | -3.834 | 0.00012628 | 0.00592948 | protein_coding | PDE1A | 5136 |
| 379 | ENSG00000100314 | 9.48 | -1.33E-01 | 3.48E-02 | -3.829 | 0.00012861 | 0.00601192 | protein_coding | CABP7 | 164633 |
| 380 | ENSG00000058404 | 121.91 | 3.31E-02 | 8.64E-03 | 3.828 | 0.00012909 | 0.00601192 | protein_coding | CAMK2B | 816 |
| 381 | ENSG00000128285 | 26.16 | -3.51E-02 | 9.17E-03 | -3.828 | 0.00012916 | 0.00601192 | protein_coding | MCHR1 | 2847 |
| 382 | ENSG00000237461 | 21.16 | 3.77E-02 | 9.87E-03 | 3.819 | 0.00013394 | 0.00620725 | lncRNA | LOC101928438 | 101928438 |
| 383 | ENSG00000143341 | 487.92 | -2.87E-02 | 7.52E-03 | -3.819 | 0.00013398 | 0.00620725 | protein_coding | HMCN1 | 83872 |
| 384 | ENSG00000231205 | 78.62 | 2.85E-02 | 7.47E-03 | 3.818 | 0.00013428 | 0.00620725 | transcribed_unprocessed_pseudogene |  |  |
| 385 | ENSG00000105641 | 462.98 | 4.41E-02 | 1.15E-02 | 3.817 | 0.00013517 | 0.00623368 | protein_coding | SLC5A5 | 6528 |
| 386 | ENSG00000154874 | 288.06 | -3.87E-02 | 1.02E-02 | -3.810 | 0.00013896 | 0.00639403 | transcribed_unprocessed_pseudogene | CCDC144B | 284047 |
| 387 | ENSG00000184672 | 18.56 | -6.79E-02 | 1.79E-02 | -3.806 | 0.00014114 | 0.00647957 | protein_coding | RALYL | 138046 |
| 388 | ENSG00000197249 | 541.22 | 2.59E-02 | 6.82E-03 | 3.796 | 0.00014695 | 0.00672066 | protein_coding | SERPINA1 | 5265 |
| 389 | ENSG00000268335 | 13.03 | 5.46E-02 | 1.44E-02 | 3.796 | 0.00014706 | 0.00672066 | processed_pseudogene | BNIP3P23 | 100421697 |
| 390 | ENSG00000005421 | 317.66 | 1.30E-01 | 3.43E-02 | 3.788 | 0.00015174 | 0.00690323 | protein_coding | PON1 | 5444 |
| 391 | ENSG00000140986 | 70.29 | 5.47E-02 | 1.45E-02 | 3.784 | 0.00015424 | 0.00696928 | protein_coding | RPL3L | 6123 |
| 392 | ENSG00000074527 | 1247.41 | 2.55E-02 | 6.74E-03 | 3.784 | 0.00015457 | 0.00696928 | protein_coding | NTN4 | 59277 |
| 393 | ENSG00000136928 | 150.97 | -4.78E-02 | 1.26E-02 | -3.783 | 0.00015514 | 0.00697937 | protein_coding | GABBR2 | 9568 |
| 394 | ENSG00000001036 | 1071.65 | 2.71E-02 | 7.17E-03 | 3.781 | 0.00015601 | 0.00700257 | protein_coding | FUCA2 | 2519 |
| 395 | ENSG00000064270 | 78.90 | 4.01E-02 | 1.06E-02 | 3.780 | 0.00015672 | 0.00701909 | protein_coding | ATP2C2 | 9914 |
| 396 | ENSG00000137252 | 8.60 | 7.71E-02 | 2.04E-02 | 3.779 | 0.00015773 | 0.00704867 | protein_coding | HCRTR2 | 3062 |
| 397 | ENSG00000165023 | 291.84 | -4.77E-02 | 1.26E-02 | -3.778 | 0.00015821 | 0.00705447 | protein_coding | DIRAS2 | 54769 |
| 398 | ENSG00000113790 | 1563.45 | 3.73E-02 | 9.88E-03 | 3.775 | 0.00015977 | 0.00708078 | protein_coding | EHHADH | 1962 |
| 399 | ENSG00000173611 | 1020.95 | 2.79E-02 | 7.38E-03 | 3.775 | 0.0001601 | 0.00708078 | protein_coding | SCAI | 286205 |
| 400 | ENSG00000248485 | 16.59 | -7.35E-02 | 1.95E-02 | -3.775 | 0.00016015 | 0.00708078 | protein_coding | PCP4L1 | 654790 |
| 401 | ENSG00000137463 | 66.55 | -4.98E-02 | 1.32E-02 | -3.774 | 0.00016088 | 0.00708919 | protein_coding | MGARP | 84709 |
| 402 | ENSG00000135406 | 23.30 | -4.42E-02 | 1.17E-02 | -3.773 | 0.0001611 | 0.00708919 | protein_coding | PRPH | 5630 |
| 403 | ENSG00000147231 | 149.41 | 2.98E-02 | 7.92E-03 | 3.767 | 0.00016514 | 0.00721976 | protein_coding | RADX | 55086 |
| 404 | ENSG00000132563 | 108.72 | -3.94E-02 | 1.05E-02 | -3.765 | 0.00016648 | 0.0072363 | protein_coding | REEP2 | 51308 |
| 405 | ENSG00000147883 | 79.31 | 2.94E-02 | 7.81E-03 | 3.765 | 0.0001666 | 0.0072363 | protein_coding | CDKN2B | 1030 |
| 406 | ENSG00000147606 | 1639.75 | 4.63E-02 | 1.23E-02 | 3.764 | 0.00016723 | 0.00724829 | protein_coding | SLC26A7 | 115111 |
| 407 | ENSG00000188732 | 1139.15 | 3.96E-02 | 1.05E-02 | 3.761 | 0.00016899 | 0.0073087 | protein_coding | FAM221A | 340277 |
| 408 | ENSG00000170873 | 2154.95 | 2.52E-02 | 6.71E-03 | 3.760 | 0.00017023 | 0.00734638 | protein_coding | MTSS1 | 9788 |
| 409 | ENSG00000173535 | 39.92 | 3.88E-02 | 1.03E-02 | 3.756 | 0.00017264 | 0.00743466 | protein_coding | TNFRSF10C | 8794 |
| 410 | ENSG00000176720 | 99.13 | 3.70E-02 | 9.85E-03 | 3.755 | 0.00017356 | 0.00745835 | protein_coding | BOK | 666 |
| 411 | ENSG00000133392 | 2287.13 | -3.75E-02 | 1.00E-02 | -3.754 | 0.00017425 | 0.00747193 | protein_coding | MYH11 | 4629 |
| 412 | ENSG00000138018 | 2299.83 | 2.81E-02 | 7.49E-03 | 3.752 | 0.00017567 | 0.00751681 | protein_coding | SELENOI | 85465 |
| 413 | ENSG00000249307 | 40.86 | -5.46E-02 | 1.46E-02 | -3.750 | 0.00017679 | 0.00754871 | lncRNA | LINC01088 | 100505875 |
| 414 | ENSG00000176058 | 290.04 | 3.62E-02 | 9.65E-03 | 3.748 | 0.00017857 | 0.00759253 | protein_coding | TPRN | 286262 |
| 415 | ENSG00000185915 | 28.28 | 4.83E-02 | 1.29E-02 | 3.740 | 0.00018419 | 0.00780114 | protein_coding | KLHL34 | 257240 |
| 416 | ENSG00000163817 | 5858.02 | 4.09E-02 | 1.09E-02 | 3.740 | 0.00018432 | 0.00780114 | protein_coding | SLC6A20 | 54716 |
| 417 | ENSG00000262359 | 62.92 | -4.22E-02 | 1.13E-02 | -3.739 | 0.00018477 | 0.00780114 | NA |  |  |
| 418 | ENSG00000152254 | 29.36 | -8.33E-02 | 2.23E-02 | -3.738 | 0.00018525 | 0.00780114 | protein_coding | G6PC2 | 57818 |
| 419 | ENSG00000160097 | 503.30 | 3.86E-02 | 1.03E-02 | 3.737 | 0.00018588 | 0.00780114 | protein_coding | FNDC5 | 252995 |
| 420 | ENSG00000060982 | 8687.31 | 4.80E-02 | 1.28E-02 | 3.737 | 0.00018597 | 0.00780114 | protein_coding | BCAT1 | 586 |
| 421 | ENSG00000079482 | 1623.86 | 2.72E-02 | 7.30E-03 | 3.725 | 0.00019517 | 0.00816066 | protein_coding | OPHN1 | 4983 |
| 422 | ENSG00000021300 | 5085.32 | 4.28E-02 | 1.15E-02 | 3.723 | 0.00019666 | 0.0082059 | protein_coding | PLEKHB1 | 58473 |
| 423 | ENSG00000172137 | 20.72 | -8.80E-02 | 2.37E-02 | -3.720 | 0.00019942 | 0.00825584 | protein_coding | CALB2 | 794 |
| 424 | ENSG00000185652 | 77.67 | -4.97E-02 | 1.34E-02 | -3.720 | 0.00019949 | 0.00825584 | protein_coding | NTF3 | 4908 |
| 425 | ENSG00000072195 | 22.06 | -4.14E-02 | 1.11E-02 | -3.717 | 0.00020138 | 0.00831676 | protein_coding | SPEG | 10290 |
| 426 | ENSG00000144837 | 481.26 | 2.85E-02 | 7.66E-03 | 3.716 | 0.00020201 | 0.00832559 | protein_coding | PLA1A | 51365 |
| 427 | ENSG00000134532 | 773.02 | 2.92E-02 | 7.87E-03 | 3.714 | 0.00020364 | 0.00834051 | protein_coding | SOX5 | 6660 |
| 428 | ENSG00000228058 | 40.78 | 5.15E-02 | 1.39E-02 | 3.714 | 0.00020392 | 0.00834051 | lncRNA | LINC01736 | 101927532 |
| 429 | ENSG00000105717 | 51.00 | 3.19E-02 | 8.60E-03 | 3.714 | 0.00020402 | 0.00834051 | protein_coding | PBX4 | 80714 |
| 430 | ENSG00000001617 | 144.60 | -2.52E-02 | 6.80E-03 | -3.713 | 0.00020491 | 0.00835964 | protein_coding | SEMA3F | 6405 |
| 431 | ENSG00000254226 | 11.22 | -6.13E-02 | 1.66E-02 | -3.702 | 0.00021396 | 0.00865895 | lncRNA | LINC01933 | 101927115 |
| 432 | ENSG00000179841 | 77.95 | 4.73E-02 | 1.28E-02 | 3.700 | 0.00021553 | 0.00870502 | protein_coding | AKAP5 | 9495 |
| 433 | ENSG00000132429 | 163.58 | 4.83E-02 | 1.31E-02 | 3.699 | 0.0002162 | 0.00871474 | protein_coding | POPDC3 | 64208 |
| 434 | ENSG00000259070 | 18.51 | 5.20E-02 | 1.41E-02 | 3.698 | 0.00021772 | 0.00875854 | lncRNA | LINC00639 | 283547 |
| 435 | ENSG00000226124 | 47.96 | 3.61E-02 | 9.76E-03 | 3.696 | 0.00021893 | 0.00878667 | protein_coding | FTCDNL1 | 348751 |
| 436 | ENSG00000113396 | 53.04 | -4.31E-02 | 1.17E-02 | -3.696 | 0.0002193 | 0.00878667 | protein_coding | SLC27A6 | 28965 |
| 437 | ENSG00000167553 | 775.00 | 2.74E-02 | 7.44E-03 | 3.690 | 0.00022444 | 0.00897483 | protein_coding | TUBA1C | 84790 |
| 438 | ENSG00000134438 | 102.32 | -3.94E-02 | 1.07E-02 | -3.689 | 0.00022497 | 0.00897849 | protein_coding | RAX | 30062 |
| 439 | ENSG00000102452 | 1607.45 | 3.92E-02 | 1.06E-02 | 3.687 | 0.00022704 | 0.00904287 | protein_coding | NALCN | 259232 |
| 440 | ENSG00000105707 | 16.05 | 3.34E-02 | 9.06E-03 | 3.685 | 0.00022853 | 0.00908423 | protein_coding | HPN | 3249 |
| 441 | ENSG00000101144 | 3326.90 | 3.58E-02 | 9.71E-03 | 3.684 | 0.00022976 | 0.00911535 | protein_coding | BMP7 | 655 |
| 442 | ENSG00000172349 | 289.82 | 2.51E-02 | 6.81E-03 | 3.683 | 0.00023071 | 0.00913508 | protein_coding | IL16 | 3603 |
| 443 | ENSG00000248174 | 17.89 | 7.22E-02 | 1.96E-02 | 3.679 | 0.00023458 | 0.00923295 | lncRNA | LINC02268 | 101928509 |
| 444 | ENSG00000258548 | 206.74 | 9.26E-02 | 2.52E-02 | 3.678 | 0.00023463 | 0.00923295 | lncRNA | LINC00645 | 100505967 |
| 445 | ENSG00000250685 | 17.71 | 6.40E-02 | 1.74E-02 | 3.678 | 0.00023502 | 0.00923295 | lncRNA | LOC654780 | 654780 |
| 446 | ENSG00000168389 | 147.22 | 4.65E-02 | 1.26E-02 | 3.677 | 0.00023564 | 0.00923949 | protein_coding | MFSD2A | 84879 |
| 447 | ENSG00000131979 | 417.31 | 2.98E-02 | 8.10E-03 | 3.677 | 0.00023626 | 0.00924202 | protein_coding | GCH1 | 2643 |
| 448 | ENSG00000171033 | 140.31 | -3.68E-02 | 1.00E-02 | -3.676 | 0.00023662 | 0.00924202 | protein_coding | PKIA | 5569 |
| 449 | ENSG00000080166 | 25570.84 | -3.39E-02 | 9.23E-03 | -3.672 | 0.00024072 | 0.00938371 | protein_coding | DCT | 1638 |
| 450 | ENSG00000125675 | 49.73 | -6.51E-02 | 1.77E-02 | -3.670 | 0.00024209 | 0.00941482 | protein_coding | GRIA3 | 2892 |
| 451 | ENSG00000144290 | 918.92 | 4.84E-02 | 1.32E-02 | 3.669 | 0.00024321 | 0.00942613 | protein_coding | SLC4A10 | 57282 |
| 452 | ENSG00000160862 | 353.53 | 2.81E-02 | 7.67E-03 | 3.668 | 0.00024423 | 0.0094477 | protein_coding | AZGP1 | 563 |
| 453 | ENSG00000167880 | 14.56 | -6.95E-02 | 1.90E-02 | -3.667 | 0.00024538 | 0.00946458 | protein_coding | EVPL | 2125 |
| 454 | ENSG00000070731 | 2896.93 | 3.34E-02 | 9.11E-03 | 3.667 | 0.00024561 | 0.00946458 | protein_coding | ST6GALNAC2 | 10610 |
| 455 | ENSG00000214193 | 64.32 | -3.27E-02 | 8.93E-03 | -3.666 | 0.00024609 | 0.00946493 | protein_coding | SH3D21 | 79729 |
| 456 | ENSG00000115896 | 2202.53 | 4.36E-02 | 1.19E-02 | 3.665 | 0.00024747 | 0.00949986 | protein_coding | PLCL1 | 5334 |
| 457 | ENSG00000172366 | 38.25 | 2.56E-02 | 7.00E-03 | 3.663 | 0.00024899 | 0.00954025 | protein_coding | MCRIP2 | 84331 |
| 458 | ENSG00000259812 | 17.71 | 6.38E-02 | 1.74E-02 | 3.661 | 0.00025115 | 0.00960476 | NA |  |  |
| 459 | ENSG00000261395 | 7.34 | 5.94E-02 | 1.62E-02 | 3.660 | 0.00025203 | 0.00961994 | transcribed_processed_pseudogene |  |  |
| 460 | ENSG00000170425 | 251.29 | 3.96E-02 | 1.08E-02 | 3.658 | 0.00025395 | 0.00967513 | protein_coding | ADORA2B | 136 |
| 461 | ENSG00000126861 | 667.83 | 7.82E-02 | 2.14E-02 | 3.657 | 0.00025512 | 0.00970128 | protein_coding | OMG | 4974 |
| 462 | ENSG00000199846 | 66.88 | 2.80E-02 | 7.66E-03 | 3.655 | 0.00025743 | 0.00975233 | snRNA | RNU1-72P | 106481955 |
| 463 | ENSG00000189139 | 31.25 | 4.34E-02 | 1.19E-02 | 3.650 | 0.00026176 | 0.00982388 | protein_coding | FSCB | 84075 |
| 464 | ENSG00000104892 | 38.62 | 3.89E-02 | 1.07E-02 | 3.650 | 0.00026272 | 0.00983073 | protein_coding | KLC3 | 147700 |
| 465 | ENSG00000178826 | 61.33 | 3.75E-02 | 1.03E-02 | 3.647 | 0.00026566 | 0.00991499 | protein_coding | TMEM139 | 135932 |
| 466 | ENSG00000109738 | 136.71 | 3.52E-02 | 9.66E-03 | 3.646 | 0.00026649 | 0.00992521 | protein_coding | GLRB | 2743 |
| 467 | ENSG00000146070 | 188.09 | 7.27E-02 | 1.99E-02 | 3.645 | 0.00026741 | 0.00992521 | protein_coding | PLA2G7 | 7941 |
| 468 | ENSG00000117122 | 41.19 | -3.29E-02 | 9.04E-03 | -3.640 | 0.00027223 | 0.01005503 | protein_coding | MFAP2 | 4237 |
| 469 | ENSG00000129270 | 71.75 | 2.94E-02 | 8.08E-03 | 3.638 | 0.00027441 | 0.01011057 | NA |  |  |
| 470 | ENSG00000152092 | 242.74 | -3.77E-02 | 1.04E-02 | -3.638 | 0.00027505 | 0.01011548 | protein_coding | ASTN1 | 460 |
| 471 | ENSG00000129596 | 2641.80 | 3.30E-02 | 9.08E-03 | 3.637 | 0.00027587 | 0.01012737 | protein_coding | CDO1 | 1036 |
| 472 | ENSG00000198855 | 651.41 | 3.39E-02 | 9.33E-03 | 3.633 | 0.0002803 | 0.01027131 | protein_coding | FICD | 11153 |
| 473 | ENSG00000113448 | 3166.50 | -2.78E-02 | 7.67E-03 | -3.629 | 0.00028499 | 0.01042418 | protein_coding | PDE4D | 5144 |
| 474 | ENSG00000156206 | 70.32 | 3.31E-02 | 9.13E-03 | 3.626 | 0.00028749 | 0.0104965 | protein_coding | CFAP161 | 161502 |
| 475 | ENSG00000036828 | 6.78 | -7.51E-02 | 2.07E-02 | -3.626 | 0.00028825 | 0.01050524 | protein_coding | CASR | 846 |
| 476 | ENSG00000124406 | 3516.06 | 2.97E-02 | 8.19E-03 | 3.625 | 0.00028887 | 0.01050884 | protein_coding | ATP8A1 | 10396 |
| 477 | ENSG00000135697 | 2025.47 | 3.72E-02 | 1.03E-02 | 3.624 | 0.00028952 | 0.01051353 | protein_coding | BCO1 | 53630 |
| 478 | ENSG00000106018 | 16.41 | -5.07E-02 | 1.40E-02 | -3.622 | 0.00029229 | 0.01059512 | protein_coding | VIPR2 | 7434 |
| 479 | ENSG00000162512 | 465.54 | 2.66E-02 | 7.34E-03 | 3.621 | 0.00029291 | 0.0105968 | protein_coding | SDC3 | 9672 |
| 480 | ENSG00000166535 | 21.44 | 3.65E-02 | 1.01E-02 | 3.621 | 0.00029339 | 0.0105968 | protein_coding | A2ML1 | 144568 |
| 481 | ENSG00000236782 | 26.12 | -3.69E-02 | 1.02E-02 | -3.620 | 0.0002947 | 0.01062498 | protein_coding |  |  |
| 482 | ENSG00000149294 | 823.81 | -3.87E-02 | 1.07E-02 | -3.617 | 0.00029759 | 0.01070428 | protein_coding | NCAM1 | 4684 |
| 483 | ENSG00000239218 | 31.80 | -3.64E-02 | 1.01E-02 | -3.614 | 0.00030177 | 0.0108026 | transcribed_processed_pseudogene |  |  |
| 484 | ENSG00000170091 | 34.39 | -6.59E-02 | 1.83E-02 | -3.608 | 0.00030859 | 0.01102713 | protein_coding | NSG2 | 51617 |
| 485 | ENSG00000122034 | 897.60 | 2.69E-02 | 7.46E-03 | 3.605 | 0.0003117 | 0.01111846 | protein_coding | GTF3A | 2971 |
| 486 | ENSG00000182612 | 611.89 | 4.01E-02 | 1.11E-02 | 3.605 | 0.00031271 | 0.01113496 | protein_coding | TSPAN10 | 83882 |
| 487 | ENSG00000183287 | 431.21 | -3.70E-02 | 1.03E-02 | -3.603 | 0.00031413 | 0.01116562 | protein_coding | CCBE1 | 147372 |
| 488 | ENSG00000129538 | 5551.45 | 3.77E-02 | 1.05E-02 | 3.600 | 0.00031839 | 0.01123186 | protein_coding | RNASE1 | 6035 |
| 489 | ENSG00000130234 | 61.82 | 3.05E-02 | 8.48E-03 | 3.600 | 0.00031878 | 0.01123186 | protein_coding | ACE2 | 59272 |
| 490 | ENSG00000182771 | 21.61 | -3.82E-02 | 1.06E-02 | -3.598 | 0.00032015 | 0.01126051 | protein_coding | GRID1 | 2894 |
| 491 | ENSG00000064309 | 517.02 | -3.46E-02 | 9.63E-03 | -3.597 | 0.00032241 | 0.01132025 | protein_coding | CDON | 50937 |
| 492 | ENSG00000148948 | 94.83 | -3.67E-02 | 1.02E-02 | -3.595 | 0.00032411 | 0.01136019 | protein_coding | LRRC4C | 57689 |
| 493 | ENSG00000137460 | 404.04 | -3.39E-02 | 9.43E-03 | -3.593 | 0.0003265 | 0.01142424 | protein_coding | FHDC1 | 85462 |
| 494 | ENSG00000186326 | 630.39 | 3.77E-02 | 1.05E-02 | 3.589 | 0.00033155 | 0.01155396 | protein_coding | RGS9BP | 388531 |
| 495 | ENSG00000137441 | 72.97 | -5.11E-02 | 1.42E-02 | -3.589 | 0.00033193 | 0.01155396 | protein_coding | FGFBP2 | 83888 |
| 496 | ENSG00000157992 | 26.01 | 2.78E-02 | 7.76E-03 | 3.588 | 0.0003334 | 0.01157346 | protein_coding | KRTCAP3 | 200634 |
| 497 | ENSG00000157542 | 4.88 | -1.34E-01 | 3.73E-02 | -3.580 | 0.0003433 | 0.0118474 | protein_coding | KCNJ6 | 3763 |
| 498 | ENSG00000107331 | 806.03 | 2.73E-02 | 7.63E-03 | 3.578 | 0.0003458 | 0.01189284 | protein_coding | ABCA2 | 20 |
| 499 | ENSG00000155816 | 80.18 | -4.68E-02 | 1.31E-02 | -3.576 | 0.00034941 | 0.01197619 | protein_coding | FMN2 | 56776 |
| 500 | ENSG00000200312 | 2422.06 | -5.24E-02 | 1.47E-02 | -3.574 | 0.00035118 | 0.01200087 | misc_RNA |  |  |
| 501 | ENSG00000134363 | 315.65 | -4.09E-02 | 1.14E-02 | -3.572 | 0.00035422 | 0.01207928 | protein_coding | FST | 10468 |
| 502 | ENSG00000212195 | 117.37 | 3.23E-02 | 9.06E-03 | 3.571 | 0.00035616 | 0.0121251 | snoRNA |  |  |
| 503 | ENSG00000141668 | 7.26 | -8.58E-02 | 2.40E-02 | -3.569 | 0.00035855 | 0.01217324 | protein_coding | CBLN2 | 147381 |
| 504 | ENSG00000203867 | 158.32 | -3.28E-02 | 9.20E-03 | -3.566 | 0.0003621 | 0.01223395 | protein_coding | RBM20 | 282996 |
| 505 | ENSG00000230615 | 45.98 | 6.67E-02 | 1.87E-02 | 3.566 | 0.00036302 | 0.01223395 | lncRNA |  |  |
| 506 | ENSG00000148482 | 9552.91 | 3.95E-02 | 1.11E-02 | 3.565 | 0.00036361 | 0.01223395 | protein_coding | SLC39A12 | 221074 |
| 507 | ENSG00000249098 | 6.60 | -7.45E-02 | 2.09E-02 | -3.564 | 0.00036557 | 0.01227951 | processed_pseudogene |  |  |
| 508 | ENSG00000146938 | 95.85 | -5.16E-02 | 1.45E-02 | -3.563 | 0.00036693 | 0.01229955 | protein_coding | NLGN4X | 57502 |
| 509 | ENSG00000201570 | 41.29 | 3.80E-02 | 1.07E-02 | 3.562 | 0.00036754 | 0.01229955 | snRNA | RNU4-56P | 106480547 |
| 510 | ENSG00000258725 | 51.80 | 2.52E-02 | 7.08E-03 | 3.562 | 0.00036861 | 0.01229955 | lncRNA | PRC1-AS1 | 100507118 |
| 511 | ENSG00000180245 | 3428.52 | 6.79E-02 | 1.91E-02 | 3.561 | 0.00036959 | 0.01230622 | protein_coding | RRH | 10692 |
| 512 | ENSG00000183570 | 100.69 | -4.48E-02 | 1.26E-02 | -3.561 | 0.00037003 | 0.01230622 | protein_coding | PCBP3 | 54039 |
| 513 | ENSG00000164220 | 927.16 | 5.29E-02 | 1.48E-02 | 3.559 | 0.00037157 | 0.01233696 | protein_coding | F2RL2 | 2151 |
| 514 | ENSG00000112893 | 4545.76 | 2.52E-02 | 7.07E-03 | 3.558 | 0.00037414 | 0.01238165 | protein_coding | MAN2A1 | 4124 |
| 515 | ENSG00000096092 | 799.80 | 3.94E-02 | 1.11E-02 | 3.556 | 0.0003763 | 0.01241233 | protein_coding | TMEM14A | 28978 |
| 516 | ENSG00000159164 | 230.26 | -2.91E-02 | 8.18E-03 | -3.552 | 0.00038172 | 0.01257047 | protein_coding | SV2A | 9900 |
| 517 | ENSG00000155749 | 54.58 | -3.05E-02 | 8.58E-03 | -3.551 | 0.00038351 | 0.01260884 | protein_coding | FLACC1 | 130540 |
| 518 | ENSG00000154975 | 89.85 | -4.12E-02 | 1.16E-02 | -3.550 | 0.00038495 | 0.0126356 | protein_coding | CA10 | 56934 |
| 519 | ENSG00000068976 | 318.89 | 3.50E-02 | 9.85E-03 | 3.550 | 0.00038578 | 0.0126361 | protein_coding | PYGM | 5837 |
| 520 | ENSG00000179008 | 28.45 | -5.34E-02 | 1.50E-02 | -3.549 | 0.00038706 | 0.012643 | protein_coding | C14orf39 | 317761 |
| 521 | ENSG00000139318 | 2940.41 | 4.15E-02 | 1.17E-02 | 3.543 | 0.00039498 | 0.01282208 | protein_coding | DUSP6 | 1848 |
| 522 | ENSG00000159267 | 1120.18 | 2.70E-02 | 7.63E-03 | 3.543 | 0.00039508 | 0.01282208 | protein_coding | HLCS | 3141 |
| 523 | ENSG00000172346 | 21.63 | -3.55E-02 | 1.00E-02 | -3.540 | 0.00039941 | 0.01291076 | protein_coding | CSDC2 | 27254 |
| 524 | ENSG00000114631 | 473.11 | 3.36E-02 | 9.50E-03 | 3.536 | 0.00040661 | 0.01311172 | protein_coding | PODXL2 | 50512 |
| 525 | ENSG00000251348 | 172.89 | 2.68E-02 | 7.58E-03 | 3.535 | 0.0004085 | 0.01315155 | processed_pseudogene |  |  |
| 526 | ENSG00000196296 | 11.42 | -5.86E-02 | 1.66E-02 | -3.531 | 0.00041413 | 0.01331159 | protein_coding | ATP2A1 | 487 |
| 527 | ENSG00000102230 | 246.42 | 3.73E-02 | 1.06E-02 | 3.526 | 0.00042261 | 0.01354085 | protein_coding | PCYT1B | 9468 |
| 528 | ENSG00000162989 | 120.08 | -3.43E-02 | 9.76E-03 | -3.511 | 0.00044569 | 0.01414553 | protein_coding | KCNJ3 | 3760 |
| 529 | ENSG00000215861 | 14.38 | -5.42E-02 | 1.54E-02 | -3.510 | 0.00044825 | 0.01420436 | transcribed_unprocessed_pseudogene |  |  |
| 530 | ENSG00000172893 | 586.31 | 3.39E-02 | 9.68E-03 | 3.505 | 0.00045669 | 0.01442648 | protein_coding | DHCR7 | 1717 |
| 531 | ENSG00000164932 | 38.66 | 3.60E-02 | 1.03E-02 | 3.503 | 0.00046081 | 0.01453399 | protein_coding | CTHRC1 | 115908 |
| 532 | ENSG00000172399 | 10.69 | -4.46E-02 | 1.27E-02 | -3.502 | 0.00046243 | 0.01453949 | protein_coding | MYOZ2 | 51778 |
| 533 | ENSG00000147255 | 23.51 | -5.09E-02 | 1.46E-02 | -3.495 | 0.00047436 | 0.01487657 | protein_coding | IGSF1 | 3547 |
| 534 | ENSG00000178980 | 703.75 | 2.53E-02 | 7.23E-03 | 3.495 | 0.00047463 | 0.01487657 | protein_coding | SELENOW | 6415 |
| 535 | ENSG00000188334 | 21.53 | 4.38E-02 | 1.25E-02 | 3.493 | 0.00047731 | 0.01493732 | protein_coding | BSPH1 | 100131137 |
| 536 | ENSG00000145949 | 141.83 | -2.86E-02 | 8.19E-03 | -3.492 | 0.00047903 | 0.01496808 | protein_coding | MYLK4 | 340156 |
| 537 | ENSG00000263553 | 6.47 | -9.06E-02 | 2.59E-02 | -3.491 | 0.00048178 | 0.01503063 | NA |  |  |
| 538 | ENSG00000070886 | 7.46 | 5.40E-02 | 1.55E-02 | 3.485 | 0.00049173 | 0.0152703 | protein_coding | EPHA8 | 2046 |
| 539 | ENSG00000006042 | 5785.18 | 4.12E-02 | 1.18E-02 | 3.484 | 0.00049384 | 0.01531221 | protein_coding | TMEM98 | 26022 |
| 540 | ENSG00000116761 | 610.35 | 3.70E-02 | 1.06E-02 | 3.482 | 0.00049718 | 0.01539198 | protein_coding | CTH | 1491 |
| 541 | ENSG00000179292 | 58.95 | 5.82E-02 | 1.68E-02 | 3.477 | 0.00050706 | 0.01560191 | protein_coding | TMEM151A | 256472 |
| 542 | ENSG00000018625 | 2100.33 | -6.98E-02 | 2.01E-02 | -3.476 | 0.0005083 | 0.01561618 | protein_coding | ATP1A2 | 477 |
| 543 | ENSG00000203801 | 11.90 | -4.12E-02 | 1.18E-02 | -3.475 | 0.00051108 | 0.01567772 | lncRNA | LINC00222 | 387111 |
| 544 | ENSG00000091137 | 523.00 | 4.09E-02 | 1.18E-02 | 3.473 | 0.00051457 | 0.01573698 | protein_coding | SLC26A4 | 5172 |
| 545 | ENSG00000258399 | 34.96 | -3.91E-02 | 1.13E-02 | -3.473 | 0.00051457 | 0.01573698 | lncRNA |  |  |
| 546 | ENSG00000091490 | 2957.52 | 4.11E-02 | 1.18E-02 | 3.473 | 0.00051548 | 0.01574074 | protein_coding | SEL1L3 | 23231 |
| 547 | ENSG00000137142 | 7.97 | 6.12E-02 | 1.76E-02 | 3.472 | 0.00051651 | 0.01574848 | protein_coding | IGFBPL1 | 347252 |
| 548 | ENSG00000163646 | 37.23 | -5.86E-02 | 1.69E-02 | -3.471 | 0.00051762 | 0.01575851 | protein_coding | CLRN1 | 7401 |
| 549 | ENSG00000100767 | 357.18 | -3.48E-02 | 1.00E-02 | -3.469 | 0.00052293 | 0.01589627 | protein_coding | PAPLN | 89932 |
| 550 | ENSG00000259871 | 22.30 | -7.86E-02 | 2.27E-02 | -3.466 | 0.00052772 | 0.01601768 | lncRNA |  |  |
| 551 | ENSG00000134873 | 253.06 | 4.85E-02 | 1.40E-02 | 3.461 | 0.00053872 | 0.01625526 | protein_coding | CLDN10 | 9071 |
| 552 | ENSG00000132000 | 34.43 | 2.59E-02 | 7.48E-03 | 3.461 | 0.0005391 | 0.01625526 | protein_coding | PODNL1 | 79883 |
| 553 | ENSG00000180871 | 50.46 | 4.95E-02 | 1.43E-02 | 3.460 | 0.00054039 | 0.01625526 | protein_coding | CXCR2 | 3579 |
| 554 | ENSG00000127588 | 8.57 | 5.03E-02 | 1.46E-02 | 3.454 | 0.00055221 | 0.01653666 | protein_coding | GNG13 | 51764 |
| 555 | ENSG00000139219 | 32.78 | -5.80E-02 | 1.68E-02 | -3.453 | 0.00055339 | 0.01654171 | protein_coding | COL2A1 | 1280 |
| 556 | ENSG00000105290 | 623.52 | 4.61E-02 | 1.33E-02 | 3.453 | 0.00055415 | 0.01654171 | protein_coding | APLP1 | 333 |
| 557 | ENSG00000106537 | 998.47 | 4.10E-02 | 1.19E-02 | 3.453 | 0.00055484 | 0.01654171 | protein_coding | TSPAN13 | 27075 |
| 558 | ENSG00000116641 | 4840.81 | 2.51E-02 | 7.28E-03 | 3.446 | 0.00056885 | 0.01685986 | protein_coding | DOCK7 | 85440 |
| 559 | ENSG00000114378 | 138.36 | 2.78E-02 | 8.08E-03 | 3.442 | 0.00057689 | 0.0170478 | protein_coding | HYAL1 | 3373 |
| 560 | ENSG00000152402 | 209.41 | -3.18E-02 | 9.23E-03 | -3.442 | 0.00057842 | 0.01706802 | protein_coding | GUCY1A2 | 2977 |
| 561 | ENSG00000131459 | 363.11 | -3.71E-02 | 1.08E-02 | -3.440 | 0.00058071 | 0.01711045 | protein_coding | GFPT2 | 9945 |
| 562 | ENSG00000040199 | 2497.54 | 2.56E-02 | 7.45E-03 | 3.439 | 0.00058339 | 0.01716436 | protein_coding | PHLPP2 | 23035 |
| 563 | ENSG00000110148 | 5.73 | -6.64E-02 | 1.93E-02 | -3.437 | 0.00058801 | 0.01727518 | protein_coding | CCKBR | 887 |
| 564 | ENSG00000181449 | 48.92 | -6.39E-02 | 1.86E-02 | -3.434 | 0.00059424 | 0.01738207 | protein_coding | SOX2 | 6657 |
| 565 | ENSG00000206195 | 39.98 | 2.68E-02 | 7.81E-03 | 3.432 | 0.00059927 | 0.01747855 | lncRNA | DUXAP8 | 503637 |
| 566 | ENSG00000247131 | 64.87 | 4.80E-02 | 1.40E-02 | 3.431 | 0.00060194 | 0.01753099 | lncRNA | LOC101928002 | 101928002 |
| 567 | ENSG00000263874 | 24.22 | -3.58E-02 | 1.05E-02 | -3.426 | 0.00061295 | 0.01777468 | protein_coding | LINC00672 | 100505576 |
| 568 | ENSG00000101670 | 1233.02 | 4.18E-02 | 1.22E-02 | 3.425 | 0.00061502 | 0.01779742 | protein_coding | LIPG | 9388 |
| 569 | ENSG00000198768 | 45.23 | -4.15E-02 | 1.21E-02 | -3.425 | 0.0006155 | 0.01779742 | protein_coding | APCDD1L | 164284 |
| 570 | ENSG00000203880 | 4845.51 | 2.58E-02 | 7.54E-03 | 3.422 | 0.00062111 | 0.01793383 | protein_coding | PCMTD2 | 55251 |
| 571 | ENSG00000232018 | 15.14 | -5.57E-02 | 1.63E-02 | -3.421 | 0.00062422 | 0.01796789 | NA |  |  |
| 572 | ENSG00000163235 | 1122.32 | 4.93E-02 | 1.44E-02 | 3.417 | 0.0006328 | 0.01807834 | protein_coding | TGFA | 7039 |
| 573 | ENSG00000152078 | 16932.81 | 4.18E-02 | 1.22E-02 | 3.417 | 0.00063329 | 0.01807834 | protein_coding | TLCD4 | 148534 |
| 574 | ENSG00000002745 | 13.99 | -7.13E-02 | 2.09E-02 | -3.414 | 0.00064103 | 0.01827345 | protein_coding | WNT16 | 51384 |
| 575 | ENSG00000101447 | 13.07 | -5.84E-02 | 1.71E-02 | -3.411 | 0.00064788 | 0.01839061 | protein_coding | FAM83D | 81610 |
| 576 | ENSG00000269839 | 24.55 | 5.40E-02 | 1.58E-02 | 3.410 | 0.00064966 | 0.01841514 | unprocessed_pseudogene |  |  |
| 577 | ENSG00000123612 | 68.06 | 3.59E-02 | 1.05E-02 | 3.408 | 0.00065437 | 0.01848322 | protein_coding | ACVR1C | 130399 |
| 578 | ENSG00000105855 | 24631.38 | 6.36E-02 | 1.87E-02 | 3.407 | 0.00065653 | 0.01850597 | protein_coding | ITGB8 | 3696 |
| 579 | ENSG00000146376 | 1608.58 | 2.91E-02 | 8.56E-03 | 3.401 | 0.00067138 | 0.01887158 | protein_coding | ARHGAP18 | 93663 |
| 580 | ENSG00000151572 | 12.00 | -5.01E-02 | 1.47E-02 | -3.401 | 0.00067261 | 0.01887993 | protein_coding | ANO4 | 121601 |
| 581 | ENSG00000153246 | 1289.32 | -2.90E-02 | 8.53E-03 | -3.399 | 0.00067557 | 0.01893654 | protein_coding | PLA2R1 | 22925 |
| 582 | ENSG00000072042 | 6610.71 | 3.85E-02 | 1.13E-02 | 3.398 | 0.00067895 | 0.01900483 | protein_coding | RDH11 | 51109 |
| 583 | ENSG00000112276 | 292.37 | 3.72E-02 | 1.10E-02 | 3.393 | 0.00069209 | 0.01928347 | protein_coding | BVES | 11149 |
| 584 | ENSG00000128578 | 13539.87 | 2.76E-02 | 8.14E-03 | 3.391 | 0.00069593 | 0.0193234 | protein_coding | STRIP2 | 57464 |
| 585 | ENSG00000261801 | 225.20 | 3.11E-02 | 9.19E-03 | 3.391 | 0.00069608 | 0.0193234 | lncRNA | LOXL1-AS1 | 100287616 |
| 586 | ENSG00000248383 | 44.19 | 2.55E-02 | 7.54E-03 | 3.389 | 0.00070197 | 0.01946006 | protein_coding | PCDHAC1 | 56135 |
| 587 | ENSG00000070159 | 297.77 | 2.59E-02 | 7.64E-03 | 3.387 | 0.00070648 | 0.01955815 | protein_coding | PTPN3 | 5774 |
| 588 | ENSG00000236699 | 25.13 | 4.86E-02 | 1.44E-02 | 3.385 | 0.00071181 | 0.01967888 | protein_coding | ARHGEF38 | 54848 |
| 589 | ENSG00000137877 | 422.92 | -4.85E-02 | 1.43E-02 | -3.381 | 0.00072339 | 0.01991693 | protein_coding | SPTBN5 | 51332 |
| 590 | ENSG00000136546 | 262.89 | -3.64E-02 | 1.08E-02 | -3.379 | 0.0007279 | 0.02001384 | protein_coding | SCN7A | 6332 |
| 591 | ENSG00000077044 | 275.92 | -4.02E-02 | 1.19E-02 | -3.374 | 0.00074105 | 0.02026478 | protein_coding | DGKD | 8527 |
| 592 | ENSG00000135916 | 2759.03 | 3.23E-02 | 9.58E-03 | 3.373 | 0.0007445 | 0.02033143 | protein_coding | ITM2C | 81618 |
| 593 | ENSG00000064547 | 34.28 | 3.31E-02 | 9.81E-03 | 3.371 | 0.00074907 | 0.02037341 | protein_coding | LPAR2 | 9170 |
| 594 | ENSG00000264222 | 17.13 | 5.08E-02 | 1.51E-02 | 3.367 | 0.00075933 | 0.02059687 | NA |  |  |
| 595 | ENSG00000228612 | 20.15 | -5.10E-02 | 1.51E-02 | -3.364 | 0.00076687 | 0.02071771 | processed_pseudogene |  |  |
| 596 | ENSG00000118271 | 126315.07 | 5.82E-02 | 1.73E-02 | 3.363 | 0.00077131 | 0.02080995 | protein_coding | TTR | 7276 |
| 597 | ENSG00000176635 | 35.74 | 4.09E-02 | 1.22E-02 | 3.362 | 0.00077291 | 0.02082518 | protein_coding | HORMAD2 | 150280 |
| 598 | ENSG00000225194 | 11.24 | -3.79E-02 | 1.13E-02 | -3.361 | 0.00077659 | 0.02084595 | lncRNA | LINC00092 | 100188953 |
| 599 | ENSG00000056736 | 172.06 | 2.52E-02 | 7.49E-03 | 3.361 | 0.00077678 | 0.02084595 | protein_coding | IL17RB | 55540 |
| 600 | ENSG00000160111 | 1888.98 | -4.77E-02 | 1.42E-02 | -3.360 | 0.00077926 | 0.02088439 | protein_coding | CPAMD8 | 27151 |
| 601 | ENSG00000240787 | 15.07 | 4.25E-02 | 1.26E-02 | 3.357 | 0.00078655 | 0.02101268 | processed_pseudogene |  |  |
| 602 | ENSG00000170088 | 2692.03 | 3.17E-02 | 9.43E-03 | 3.357 | 0.00078717 | 0.02101268 | protein_coding | TMEM192 | 201931 |
| 603 | ENSG00000230470 | 29.49 | -3.33E-02 | 9.92E-03 | -3.357 | 0.00078932 | 0.02101448 | lncRNA |  |  |
| 604 | ENSG00000134982 | 6013.87 | 2.97E-02 | 8.85E-03 | 3.355 | 0.0007923 | 0.02106605 | protein_coding | APC | 324 |
| 605 | ENSG00000184162 | 24.81 | 2.74E-02 | 8.16E-03 | 3.355 | 0.00079476 | 0.02109222 | protein_coding | NR2C2AP | 126382 |
| 606 | ENSG00000115290 | 24.26 | -3.60E-02 | 1.07E-02 | -3.352 | 0.00080299 | 0.02121019 | protein_coding | GRB14 | 2888 |
| 607 | ENSG00000259495 | 24.49 | 2.87E-02 | 8.56E-03 | 3.349 | 0.00081011 | 0.02136697 | lncRNA |  |  |
| 608 | ENSG00000170558 | 945.75 | -3.89E-02 | 1.16E-02 | -3.346 | 0.00082017 | 0.02156463 | protein_coding | CDH2 | 1000 |
| 609 | ENSG00000114805 | 846.13 | 2.91E-02 | 8.71E-03 | 3.344 | 0.00082595 | 0.02164667 | protein_coding | PLCH1 | 23007 |
| 610 | ENSG00000154274 | 38.83 | 3.69E-02 | 1.11E-02 | 3.341 | 0.00083513 | 0.0217739 | protein_coding | C4orf19 | 55286 |
| 611 | ENSG00000108984 | 443.80 | 2.96E-02 | 8.86E-03 | 3.339 | 0.00084146 | 0.02191062 | protein_coding | MAP2K6 | 5608 |
| 612 | ENSG00000152256 | 333.92 | -2.65E-02 | 7.95E-03 | -3.338 | 0.00084266 | 0.02191349 | protein_coding | PDK1 | 5163 |
| 613 | ENSG00000232689 | 64.90 | 2.98E-02 | 8.92E-03 | 3.338 | 0.00084491 | 0.02194378 | NA |  |  |
| 614 | ENSG00000197415 | 2203.09 | 2.95E-02 | 8.84E-03 | 3.337 | 0.00084631 | 0.02195189 | protein_coding | VEPH1 | 79674 |
| 615 | ENSG00000035664 | 73.44 | -2.96E-02 | 8.86E-03 | -3.337 | 0.00084744 | 0.02195284 | protein_coding | DAPK2 | 23604 |
| 616 | ENSG00000163293 | 32.39 | -4.32E-02 | 1.30E-02 | -3.336 | 0.00085044 | 0.02197561 | protein_coding | NIPAL1 | 152519 |
| 617 | ENSG00000133958 | 67.83 | -5.74E-02 | 1.72E-02 | -3.332 | 0.00086331 | 0.0221824 | protein_coding | UNC79 | 57578 |
| 618 | ENSG00000176788 | 1668.91 | 3.05E-02 | 9.16E-03 | 3.331 | 0.00086401 | 0.0221824 | protein_coding | BASP1 | 10409 |
| 619 | ENSG00000134533 | 211.72 | -2.64E-02 | 7.93E-03 | -3.326 | 0.00088256 | 0.02260115 | protein_coding | RERG | 85004 |
| 620 | ENSG00000253479 | 12.24 | 4.96E-02 | 1.49E-02 | 3.320 | 0.00089942 | 0.02291655 | lncRNA | LINC01603 | 100505739 |
| 621 | ENSG00000130226 | 30.68 | -5.17E-02 | 1.56E-02 | -3.317 | 0.00090862 | 0.02303091 | protein_coding | DPP6 | 1804 |
| 622 | ENSG00000164175 | 1166.42 | 3.33E-02 | 1.00E-02 | 3.317 | 0.00090963 | 0.02303091 | protein_coding | SLC45A2 | 51151 |
| 623 | ENSG00000183715 | 3290.63 | 5.95E-02 | 1.79E-02 | 3.316 | 0.00091243 | 0.02306596 | protein_coding | OPCML | 4978 |
| 624 | ENSG00000115474 | 6338.95 | 4.27E-02 | 1.29E-02 | 3.316 | 0.0009133 | 0.02306596 | protein_coding | KCNJ13 | 3769 |
| 625 | ENSG00000146411 | 12249.94 | 3.32E-02 | 1.00E-02 | 3.315 | 0.00091585 | 0.02310134 | protein_coding | SLC2A12 | 154091 |
| 626 | ENSG00000173175 | 47.67 | -3.24E-02 | 9.78E-03 | -3.314 | 0.00092108 | 0.0231981 | protein_coding | ADCY5 | 111 |
| 627 | ENSG00000149972 | 13.69 | -4.75E-02 | 1.43E-02 | -3.313 | 0.0009224 | 0.0231981 | protein_coding | CNTN5 | 53942 |
| 628 | ENSG00000135469 | 238.23 | 2.86E-02 | 8.62E-03 | 3.313 | 0.00092404 | 0.0231981 | protein_coding | COQ10A | 93058 |
| 629 | ENSG00000186081 | 19.68 | 5.40E-02 | 1.63E-02 | 3.313 | 0.00092429 | 0.0231981 | protein_coding | KRT5 | 3852 |
| 630 | ENSG00000014257 | 84.60 | -3.53E-02 | 1.07E-02 | -3.309 | 0.00093606 | 0.02343764 | protein_coding | ACP3 | 55 |
| 631 | ENSG00000182118 | 324.32 | 2.82E-02 | 8.54E-03 | 3.305 | 0.00094978 | 0.0237492 | protein_coding | FAM89A | 375061 |
| 632 | ENSG00000172575 | 193.70 | -4.42E-02 | 1.34E-02 | -3.304 | 0.00095234 | 0.02377424 | protein_coding | RASGRP1 | 10125 |
| 633 | ENSG00000151962 | 98.11 | 3.35E-02 | 1.01E-02 | 3.304 | 0.00095347 | 0.02377424 | protein_coding | RBM46 | 166863 |
| 634 | ENSG00000137868 | 1276.32 | 2.69E-02 | 8.13E-03 | 3.303 | 0.00095564 | 0.02377424 | protein_coding | STRA6 | 64220 |
| 635 | ENSG00000104332 | 7239.77 | 2.61E-02 | 7.90E-03 | 3.303 | 0.00095668 | 0.02377424 | protein_coding | SFRP1 | 6422 |
| 636 | ENSG00000141314 | 41.16 | -4.32E-02 | 1.31E-02 | -3.302 | 0.00095938 | 0.02378571 | protein_coding | RHBDL3 | 162494 |
| 637 | ENSG00000230910 | 10.00 | -5.15E-02 | 1.56E-02 | -3.302 | 0.0009595 | 0.02378571 | lncRNA |  |  |
| 638 | ENSG00000122735 | 9.40 | -5.42E-02 | 1.64E-02 | -3.302 | 0.00096162 | 0.02380904 | protein_coding | DNAI1 | 27019 |
| 639 | ENSG00000174527 | 16.26 | 3.63E-02 | 1.10E-02 | 3.301 | 0.00096369 | 0.02383097 | protein_coding | MYO1H | 283446 |
| 640 | ENSG00000248397 | 16.80 | 5.16E-02 | 1.56E-02 | 3.300 | 0.000967 | 0.02388352 | lncRNA |  |  |
| 641 | ENSG00000067057 | 823.79 | -2.92E-02 | 8.86E-03 | -3.298 | 0.00097546 | 0.02400512 | protein_coding | PFKP | 5214 |
| 642 | ENSG00000135447 | 11.88 | -5.61E-02 | 1.70E-02 | -3.296 | 0.00097972 | 0.0240797 | protein_coding | PPP1R1A | 5502 |
| 643 | ENSG00000226852 | 5.63 | -5.64E-02 | 1.71E-02 | -3.295 | 0.00098272 | 0.02412387 | NA |  |  |
| 644 | ENSG00000119913 | 210.29 | 9.70E-02 | 2.94E-02 | 3.295 | 0.00098555 | 0.02412834 | protein_coding | TECTB | 6975 |
| 645 | ENSG00000140955 | 60.10 | 5.69E-02 | 1.73E-02 | 3.295 | 0.00098582 | 0.02412834 | protein_coding | ADAD2 | 161931 |
| 646 | ENSG00000204334 | 46.83 | 3.37E-02 | 1.02E-02 | 3.289 | 0.00100374 | 0.02452038 | protein_coding | ERICH2 | 285141 |
| 647 | ENSG00000100678 | 31.50 | -5.98E-02 | 1.82E-02 | -3.288 | 0.00100742 | 0.02458061 | protein_coding | SLC8A3 | 6547 |
| 648 | ENSG00000080986 | 70.81 | 4.61E-02 | 1.40E-02 | 3.288 | 0.00101031 | 0.02462127 | protein_coding | NDC80 | 10403 |
| 649 | ENSG00000177098 | 51.96 | -3.80E-02 | 1.16E-02 | -3.287 | 0.00101203 | 0.02463349 | protein_coding | SCN4B | 6330 |
| 650 | ENSG00000149451 | 23.13 | -3.80E-02 | 1.16E-02 | -3.286 | 0.00101579 | 0.02469501 | protein_coding | ADAM33 | 80332 |
| 651 | ENSG00000044524 | 167.38 | -3.26E-02 | 9.92E-03 | -3.285 | 0.00101937 | 0.0247522 | protein_coding | EPHA3 | 2042 |
| 652 | ENSG00000139970 | 167.30 | -2.61E-02 | 7.97E-03 | -3.280 | 0.00103833 | 0.02515195 | protein_coding | RTN1 | 6252 |
| 653 | ENSG00000177459 | 418.52 | -2.90E-02 | 8.86E-03 | -3.277 | 0.0010504 | 0.02536703 | protein_coding | ERICH5 | 203111 |
| 654 | ENSG00000150556 | 20.41 | -7.31E-02 | 2.23E-02 | -3.273 | 0.00106237 | 0.02557122 | protein_coding | LYPD6B | 130576 |
| 655 | ENSG00000078725 | 65.03 | -3.43E-02 | 1.05E-02 | -3.273 | 0.00106325 | 0.02557122 | protein_coding | BRINP1 | 1620 |
| 656 | ENSG00000118898 | 292.39 | -2.61E-02 | 8.00E-03 | -3.269 | 0.00107788 | 0.02586148 | protein_coding | PPL | 5493 |
| 657 | ENSG00000135472 | 56.37 | -5.41E-02 | 1.66E-02 | -3.266 | 0.00109132 | 0.02612172 | protein_coding | FAIM2 | 23017 |
| 658 | ENSG00000184599 | 87.21 | 4.58E-02 | 1.40E-02 | 3.264 | 0.00110011 | 0.02625005 | protein_coding | TAFA3 | 284467 |
| 659 | ENSG00000232036 | 15.05 | -5.03E-02 | 1.54E-02 | -3.263 | 0.00110189 | 0.02625005 | processed_pseudogene | LOC100129573 | 100129573 |
| 660 | ENSG00000231865 | 38.32 | -2.67E-02 | 8.20E-03 | -3.262 | 0.00110513 | 0.02626513 | NA |  |  |
| 661 | ENSG00000234978 | 37.39 | 3.53E-02 | 1.08E-02 | 3.255 | 0.00113317 | 0.02689975 | NA |  |  |
| 662 | ENSG00000103196 | 2710.22 | -3.36E-02 | 1.03E-02 | -3.254 | 0.00113964 | 0.02702148 | protein_coding | CRISPLD2 | 83716 |
| 663 | ENSG00000051128 | 122.05 | 2.66E-02 | 8.17E-03 | 3.250 | 0.00115439 | 0.02730713 | protein_coding | HOMER3 | 9454 |
| 664 | ENSG00000121207 | 21670.18 | 6.06E-02 | 1.87E-02 | 3.249 | 0.00115914 | 0.02735526 | protein_coding | LRAT | 9227 |
| 665 | ENSG00000197696 | 9.34 | -4.65E-02 | 1.43E-02 | -3.244 | 0.0011776 | 0.02772581 | protein_coding | NMB | 4828 |
| 666 | ENSG00000158747 | 63.55 | -3.11E-02 | 9.60E-03 | -3.244 | 0.00118059 | 0.02776385 | protein_coding | NBL1 | 4681 |
| 667 | ENSG00000223893 | 19.31 | -6.95E-02 | 2.14E-02 | -3.242 | 0.00118787 | 0.02787001 | processed_pseudogene | GNL2P1 | 100270790 |
| 668 | ENSG00000171492 | 869.89 | 2.59E-02 | 8.00E-03 | 3.241 | 0.00119154 | 0.02792355 | protein_coding | LRRC8D | 55144 |
| 669 | ENSG00000214336 | 17.15 | -7.14E-02 | 2.21E-02 | -3.234 | 0.0012211 | 0.02851683 | protein_coding | FOXI3 | 344167 |
| 670 | ENSG00000099769 | 21.99 | 5.26E-02 | 1.63E-02 | 3.233 | 0.00122539 | 0.02858385 | protein_coding | IGFALS | 3483 |
| 671 | ENSG00000197705 | 93.88 | -4.04E-02 | 1.25E-02 | -3.231 | 0.00123421 | 0.02875642 | protein_coding | KLHL14 | 57565 |
| 672 | ENSG00000152253 | 6.55 | -9.28E-02 | 2.87E-02 | -3.228 | 0.00124544 | 0.02890557 | protein_coding | SPC25 | 57405 |
| 673 | ENSG00000221995 | 15.53 | -4.19E-02 | 1.30E-02 | -3.228 | 0.00124635 | 0.02890557 | protein_coding | TIAF1 | 9220 |
| 674 | ENSG00000080644 | 6473.22 | 4.01E-02 | 1.24E-02 | 3.225 | 0.00126135 | 0.0291661 | protein_coding | CHRNA3 | 1136 |
| 675 | ENSG00000228956 | 66.49 | 3.88E-02 | 1.20E-02 | 3.225 | 0.00126193 | 0.0291661 | lncRNA |  |  |
| 676 | ENSG00000169594 | 55.75 | -3.75E-02 | 1.16E-02 | -3.223 | 0.00126796 | 0.02919205 | protein_coding | BNC1 | 646 |
| 677 | ENSG00000171885 | 291.47 | -6.48E-02 | 2.01E-02 | -3.223 | 0.00126988 | 0.02919205 | protein_coding | AQP4 | 361 |
| 678 | ENSG00000232977 | 10.55 | 4.09E-02 | 1.27E-02 | 3.220 | 0.0012808 | 0.0293396 | lncRNA | LINC00327 | 100506697 |
| 679 | ENSG00000146072 | 65.88 | -2.78E-02 | 8.64E-03 | -3.220 | 0.00128134 | 0.0293396 | protein_coding | TNFRSF21 | 27242 |
| 680 | ENSG00000234840 | 48.21 | 3.04E-02 | 9.44E-03 | 3.220 | 0.00128244 | 0.0293396 | lncRNA | LINC01239 | 441389 |
| 681 | ENSG00000189134 | 38.15 | -3.44E-02 | 1.07E-02 | -3.220 | 0.00128253 | 0.0293396 | protein_coding | NKAPL | 222698 |
| 682 | ENSG00000187266 | 27.76 | -2.61E-02 | 8.14E-03 | -3.214 | 0.00131077 | 0.0298838 | protein_coding | EPOR | 2057 |
| 683 | ENSG00000197582 | 149.43 | 3.64E-02 | 1.13E-02 | 3.210 | 0.00132926 | 0.0302028 | processed_pseudogene |  |  |
| 684 | ENSG00000147234 | 43.17 | 3.18E-02 | 9.90E-03 | 3.208 | 0.00133463 | 0.03025671 | protein_coding | FRMPD3 | 84443 |
| 685 | ENSG00000102996 | 246.58 | 2.61E-02 | 8.15E-03 | 3.208 | 0.00133881 | 0.03031732 | protein_coding | MMP15 | 4324 |
| 686 | ENSG00000114251 | 189.67 | -2.52E-02 | 7.86E-03 | -3.206 | 0.00134369 | 0.0303937 | protein_coding | WNT5A | 7474 |
| 687 | ENSG00000259583 | 162.70 | 3.61E-02 | 1.13E-02 | 3.204 | 0.00135621 | 0.03057036 | lncRNA | LOC101927751 | 101927751 |
| 688 | ENSG00000135917 | 33.91 | -3.19E-02 | 9.95E-03 | -3.203 | 0.00135908 | 0.03057036 | protein_coding | SLC19A3 | 80704 |
| 689 | ENSG00000163359 | 180.22 | -2.70E-02 | 8.44E-03 | -3.203 | 0.00135909 | 0.03057036 | protein_coding | COL6A3 | 1293 |
| 690 | ENSG00000167703 | 171.80 | 2.84E-02 | 8.87E-03 | 3.200 | 0.00137263 | 0.03077296 | protein_coding | SLC43A2 | 124935 |
| 691 | ENSG00000165731 | 13.68 | -5.30E-02 | 1.66E-02 | -3.198 | 0.00138565 | 0.03102934 | protein_coding | RET | 5979 |
| 692 | ENSG00000171872 | 6.71 | 6.91E-02 | 2.16E-02 | 3.197 | 0.0013883 | 0.03105413 | protein_coding | KLF17 | 128209 |
| 693 | ENSG00000140538 | 621.45 | -2.93E-02 | 9.18E-03 | -3.195 | 0.00139923 | 0.03126389 | protein_coding | NTRK3 | 4916 |
| 694 | ENSG00000226200 | 152.81 | 3.06E-02 | 9.58E-03 | 3.194 | 0.00140485 | 0.03135485 | lncRNA |  |  |
| 695 | ENSG00000131771 | 137.74 | -7.63E-02 | 2.39E-02 | -3.192 | 0.0014141 | 0.03149153 | protein_coding | PPP1R1B | 84152 |
| 696 | ENSG00000170579 | 75.63 | -2.60E-02 | 8.17E-03 | -3.190 | 0.00142498 | 0.03169871 | protein_coding | DLGAP1 | 9229 |
| 697 | ENSG00000265787 | 57.99 | 4.00E-02 | 1.26E-02 | 3.188 | 0.00143477 | 0.0318813 | transcribed_unprocessed_pseudogene | CYP4F35P | 284233 |
| 698 | ENSG00000112183 | 162.49 | -3.01E-02 | 9.45E-03 | -3.184 | 0.0014517 | 0.0321865 | protein_coding | RBM24 | 221662 |
| 699 | ENSG00000127328 | 3741.00 | 2.57E-02 | 8.07E-03 | 3.184 | 0.00145375 | 0.03219652 | protein_coding | RAB3IP | 117177 |
| 700 | ENSG00000099282 | 90.13 | 2.88E-02 | 9.04E-03 | 3.183 | 0.00145851 | 0.0322051 | protein_coding | TSPAN15 | 23555 |
| 701 | ENSG00000171241 | 16.42 | -4.81E-02 | 1.51E-02 | -3.183 | 0.00145949 | 0.0322051 | protein_coding | SHCBP1 | 79801 |
| 702 | ENSG00000108018 | 262.88 | -4.82E-02 | 1.51E-02 | -3.182 | 0.00146053 | 0.0322051 | protein_coding | SORCS1 | 114815 |
| 703 | ENSG00000183036 | 1165.33 | 2.72E-02 | 8.56E-03 | 3.179 | 0.00147889 | 0.03243253 | protein_coding | PCP4 | 5121 |
| 704 | ENSG00000067840 | 55.20 | -2.95E-02 | 9.30E-03 | -3.177 | 0.00148685 | 0.03254377 | protein_coding | PDZD4 | 57595 |
| 705 | ENSG00000140600 | 16.84 | -5.00E-02 | 1.57E-02 | -3.177 | 0.00148847 | 0.03254377 | protein_coding | SH3GL3 | 6457 |
| 706 | ENSG00000130707 | 406.17 | -2.75E-02 | 8.64E-03 | -3.177 | 0.0014888 | 0.03254377 | protein_coding | ASS1 | 445 |
| 707 | ENSG00000175084 | 15.82 | -6.88E-02 | 2.17E-02 | -3.175 | 0.0014985 | 0.03268492 | protein_coding | DES | 1674 |
| 708 | ENSG00000258734 | 42.40 | 4.87E-02 | 1.53E-02 | 3.174 | 0.00150256 | 0.03273792 | processed_pseudogene |  |  |
| 709 | ENSG00000234553 | 24.95 | 4.28E-02 | 1.35E-02 | 3.174 | 0.0015047 | 0.03274906 | lncRNA |  |  |
| 710 | ENSG00000235621 | 12.07 | 4.05E-02 | 1.28E-02 | 3.169 | 0.00152741 | 0.03316079 | lncRNA | LINC00494 | 284749 |
| 711 | ENSG00000120217 | 141.40 | -2.70E-02 | 8.51E-03 | -3.168 | 0.00153336 | 0.03322055 | protein_coding | CD274 | 29126 |
| 712 | ENSG00000179915 | 72.46 | -4.38E-02 | 1.38E-02 | -3.167 | 0.00153938 | 0.03328818 | protein_coding | NRXN1 | 9378 |
| 713 | ENSG00000153162 | 188.85 | 3.39E-02 | 1.07E-02 | 3.165 | 0.00155186 | 0.03345045 | protein_coding | BMP6 | 654 |
| 714 | ENSG00000198590 | 63.89 | -3.34E-02 | 1.06E-02 | -3.160 | 0.00157506 | 0.03387815 | lncRNA | C3orf35 | 339883 |
| 715 | ENSG00000206549 | 5.34 | -6.60E-02 | 2.09E-02 | -3.150 | 0.00163513 | 0.03490261 | protein_coding |  |  |
| 716 | ENSG00000240253 | 7.91 | 5.29E-02 | 1.68E-02 | 3.149 | 0.00163602 | 0.03490261 | transcribed_unprocessed_pseudogene |  |  |
| 717 | ENSG00000171189 | 110.80 | -5.42E-02 | 1.72E-02 | -3.148 | 0.00164592 | 0.03495464 | protein_coding | GRIK1 | 2897 |
| 718 | ENSG00000240758 | 36.40 | -5.15E-02 | 1.64E-02 | -3.144 | 0.00166436 | 0.03530905 | lncRNA |  |  |
| 719 | ENSG00000244242 | 180.40 | 2.86E-02 | 9.10E-03 | 3.143 | 0.00167413 | 0.03544161 | protein_coding | IFITM10 | 402778 |
| 720 | ENSG00000153283 | 99.67 | -3.89E-02 | 1.24E-02 | -3.139 | 0.00169627 | 0.03579258 | protein_coding | CD96 | 10225 |
| 721 | ENSG00000170540 | 7395.89 | 2.77E-02 | 8.84E-03 | 3.138 | 0.0017023 | 0.03580195 | protein_coding | ARL6IP1 | 23204 |
| 722 | ENSG00000050438 | 396.97 | 2.88E-02 | 9.19E-03 | 3.138 | 0.00170265 | 0.03580195 | protein_coding | SLC4A8 | 9498 |
| 723 | ENSG00000252110 | 11.49 | 3.97E-02 | 1.27E-02 | 3.137 | 0.00170536 | 0.03580195 | NA |  |  |
| 724 | ENSG00000147036 | 23.57 | -5.01E-02 | 1.60E-02 | -3.134 | 0.00172487 | 0.0361338 | protein_coding | LANCL3 | 347404 |
| 725 | ENSG00000188707 | 52.06 | 3.18E-02 | 1.01E-02 | 3.134 | 0.00172589 | 0.0361338 | protein_coding | ZBED6CL | 113763 |
| 726 | ENSG00000261051 | 40.81 | -2.97E-02 | 9.49E-03 | -3.134 | 0.00172685 | 0.0361338 | lncRNA |  |  |
| 727 | ENSG00000087250 | 7.87 | -4.89E-02 | 1.56E-02 | -3.133 | 0.0017328 | 0.03615207 | protein_coding | MT3 | 4504 |
| 728 | ENSG00000013293 | 37.57 | -6.48E-02 | 2.07E-02 | -3.132 | 0.00173712 | 0.03618527 | protein_coding | SLC7A14 | 57709 |
| 729 | ENSG00000137819 | 114.47 | -3.12E-02 | 9.97E-03 | -3.132 | 0.00173798 | 0.03618527 | protein_coding | PAQR5 | 54852 |
| 730 | ENSG00000225465 | 37.10 | 2.96E-02 | 9.47E-03 | 3.128 | 0.00175944 | 0.03655645 | lncRNA |  |  |
| 731 | ENSG00000136531 | 80.04 | -4.19E-02 | 1.34E-02 | -3.127 | 0.00176813 | 0.0366992 | protein_coding | SCN2A | 6326 |
| 732 | ENSG00000271401 | 8.95 | -3.93E-02 | 1.26E-02 | -3.122 | 0.00179466 | 0.03713515 | lncRNA |  |  |
| 733 | ENSG00000184305 | 332.57 | -3.43E-02 | 1.10E-02 | -3.122 | 0.00179801 | 0.03715123 | protein_coding | CCSER1 | 401145 |
| 734 | ENSG00000109062 | 995.14 | 2.56E-02 | 8.21E-03 | 3.121 | 0.00179975 | 0.03715123 | protein_coding | SLC9A3R1 | 9368 |
| 735 | ENSG00000162598 | 95.24 | 3.25E-02 | 1.04E-02 | 3.119 | 0.00181741 | 0.03738728 | protein_coding | C1orf87 | 127795 |
| 736 | ENSG00000164051 | 284.58 | 2.55E-02 | 8.18E-03 | 3.117 | 0.00182935 | 0.03751307 | protein_coding | CCDC51 | 79714 |
| 737 | ENSG00000119125 | 54.41 | -2.82E-02 | 9.04E-03 | -3.115 | 0.00183931 | 0.03767217 | protein_coding | GDA | 9615 |
| 738 | ENSG00000144834 | 28.42 | -6.53E-02 | 2.10E-02 | -3.112 | 0.00185776 | 0.03798088 | protein_coding | TAGLN3 | 29114 |
| 739 | ENSG00000178568 | 1433.56 | 3.07E-02 | 9.85E-03 | 3.112 | 0.00185841 | 0.03798088 | protein_coding | ERBB4 | 2066 |
| 740 | ENSG00000113749 | 31.19 | 3.07E-02 | 9.86E-03 | 3.111 | 0.00186274 | 0.03798276 | protein_coding | HRH2 | 3274 |
| 741 | ENSG00000120756 | 1409.74 | 3.04E-02 | 9.76E-03 | 3.111 | 0.0018639 | 0.03798276 | protein_coding | PLS1 | 5357 |
| 742 | ENSG00000125409 | 8.15 | -4.42E-02 | 1.42E-02 | -3.108 | 0.0018812 | 0.03823464 | protein_coding | TEKT3 | 64518 |
| 743 | ENSG00000119614 | 107.22 | -6.77E-02 | 2.18E-02 | -3.108 | 0.00188173 | 0.03823464 | protein_coding | VSX2 | 338917 |
| 744 | ENSG00000261185 | 10.65 | -8.87E-02 | 2.86E-02 | -3.104 | 0.00191015 | 0.03861302 | NA |  |  |
| 745 | ENSG00000137766 | 872.82 | -3.53E-02 | 1.14E-02 | -3.099 | 0.00193965 | 0.03901375 | protein_coding | UNC13C | 440279 |
| 746 | ENSG00000175229 | 21.18 | -3.34E-02 | 1.08E-02 | -3.094 | 0.00197248 | 0.03947703 | protein_coding | GAL3ST3 | 89792 |
| 747 | ENSG00000136960 | 37845.93 | 3.46E-02 | 1.12E-02 | 3.094 | 0.00197483 | 0.03948479 | protein_coding | ENPP2 | 5168 |
| 748 | ENSG00000166426 | 292.46 | -2.86E-02 | 9.26E-03 | -3.086 | 0.00202602 | 0.04018941 | protein_coding | CRABP1 | 1381 |
| 749 | ENSG00000203930 | 10.72 | -4.70E-02 | 1.52E-02 | -3.086 | 0.00203155 | 0.04025945 | lncRNA | LINC00632 | 286411 |
| 750 | ENSG00000089250 | 23.18 | -4.00E-02 | 1.30E-02 | -3.082 | 0.00205375 | 0.04061267 | protein_coding | NOS1 | 4842 |
| 751 | ENSG00000254781 | 7.45 | 4.92E-02 | 1.60E-02 | 3.082 | 0.00205547 | 0.04061267 | processed_pseudogene | GVINP2 | 100128135 |
| 752 | ENSG00000105889 | 42.76 | 3.39E-02 | 1.10E-02 | 3.082 | 0.00205743 | 0.04061267 | protein_coding | LOC401312 | 401312 |
| 753 | ENSG00000131188 | 8.97 | 4.66E-02 | 1.51E-02 | 3.080 | 0.00207313 | 0.04071218 | protein_coding | PRR7 | 80758 |
| 754 | ENSG00000111199 | 184.23 | 2.85E-02 | 9.26E-03 | 3.079 | 0.0020763 | 0.04071218 | protein_coding | TRPV4 | 59341 |
| 755 | ENSG00000170264 | 1536.17 | 2.62E-02 | 8.51E-03 | 3.077 | 0.00208754 | 0.04076764 | protein_coding | FAM161A | 84140 |
| 756 | ENSG00000120708 | 741.18 | -3.02E-02 | 9.81E-03 | -3.075 | 0.00210775 | 0.04111956 | protein_coding | TGFBI | 7045 |
| 757 | ENSG00000187398 | 9.21 | -4.52E-02 | 1.47E-02 | -3.073 | 0.00212035 | 0.04128838 | protein_coding | LUZP2 | 338645 |
| 758 | ENSG00000228613 | 5.90 | -6.30E-02 | 2.05E-02 | -3.070 | 0.00213799 | 0.04155158 | lncRNA |  |  |
| 759 | ENSG00000238922 | 8.88 | 4.03E-02 | 1.31E-02 | 3.069 | 0.00215135 | 0.0416674 | NA |  |  |
| 760 | ENSG00000169891 | 1543.01 | 2.68E-02 | 8.74E-03 | 3.068 | 0.00215148 | 0.0416674 | protein_coding | REPS2 | 9185 |
| 761 | ENSG00000186487 | 25.67 | -6.13E-02 | 2.00E-02 | -3.068 | 0.00215222 | 0.0416674 | protein_coding | MYT1L | 23040 |
| 762 | ENSG00000173598 | 1634.15 | -2.51E-02 | 8.17E-03 | -3.068 | 0.00215687 | 0.04167964 | protein_coding | NUDT4 | 11163 |
| 763 | ENSG00000157693 | 1278.04 | 2.59E-02 | 8.44E-03 | 3.065 | 0.0021782 | 0.04193557 | protein_coding | TMEM268 | 203197 |
| 764 | ENSG00000143028 | 29.07 | 2.51E-02 | 8.18E-03 | 3.064 | 0.00218059 | 0.04193557 | protein_coding | SYPL2 | 284612 |
| 765 | ENSG00000258670 | 13.56 | -5.79E-02 | 1.89E-02 | -3.063 | 0.00218952 | 0.04205365 | lncRNA |  |  |
| 766 | ENSG00000165566 | 580.93 | -3.56E-02 | 1.16E-02 | -3.063 | 0.00219095 | 0.04205365 | protein_coding | AMER2 | 219287 |
| 767 | ENSG00000121039 | 18546.67 | 3.09E-02 | 1.01E-02 | 3.062 | 0.00219728 | 0.04209495 | protein_coding | RDH10 | 157506 |
| 768 | ENSG00000054938 | 27.80 | -3.99E-02 | 1.30E-02 | -3.057 | 0.00223262 | 0.04273144 | protein_coding | CHRDL2 | 25884 |
| 769 | ENSG00000163618 | 342.87 | -6.47E-02 | 2.12E-02 | -3.056 | 0.00224387 | 0.04278943 | protein_coding | CADPS | 8618 |
| 770 | ENSG00000168453 | 149.10 | 3.11E-02 | 1.02E-02 | 3.052 | 0.00226946 | 0.04300419 | protein_coding | HR | 55806 |
| 771 | ENSG00000245248 | 7.78 | -4.46E-02 | 1.46E-02 | -3.052 | 0.00227007 | 0.04300419 | lncRNA |  |  |
| 772 | ENSG00000260512 | 123.28 | -3.21E-02 | 1.05E-02 | -3.052 | 0.00227124 | 0.04300419 | NA |  |  |
| 773 | ENSG00000168280 | 542.97 | -3.06E-02 | 1.00E-02 | -3.050 | 0.00228992 | 0.04325301 | protein_coding | KIF5C | 3800 |
| 774 | ENSG00000154118 | 18.73 | -5.91E-02 | 1.94E-02 | -3.048 | 0.00230476 | 0.04345203 | protein_coding | JPH3 | 57338 |
| 775 | ENSG00000169562 | 21.83 | 7.89E-02 | 2.59E-02 | 3.045 | 0.00232923 | 0.04376148 | protein_coding | GJB1 | 2705 |
| 776 | ENSG00000107295 | 128.90 | -4.95E-02 | 1.63E-02 | -3.044 | 0.00233075 | 0.04376148 | protein_coding | SH3GL2 | 6456 |
| 777 | ENSG00000079435 | 172.54 | 3.16E-02 | 1.04E-02 | 3.044 | 0.00233638 | 0.04376148 | protein_coding | LIPE | 3991 |
| 778 | ENSG00000087495 | 10.13 | -5.15E-02 | 1.69E-02 | -3.042 | 0.00234679 | 0.04387498 | protein_coding | PHACTR3 | 116154 |
| 779 | ENSG00000103257 | 1173.94 | 2.56E-02 | 8.43E-03 | 3.040 | 0.00236438 | 0.04402904 | protein_coding | SLC7A5 | 8140 |
| 780 | ENSG00000154898 | 34.75 | -2.94E-02 | 9.67E-03 | -3.040 | 0.00236878 | 0.04402904 | transcribed_processed_pseudogene |  |  |
| 781 | ENSG00000239467 | 57.39 | 2.83E-02 | 9.32E-03 | 3.040 | 0.00236968 | 0.04402904 | lncRNA | ERICH2 | 285141 |
| 782 | ENSG00000150551 | 48.51 | -4.08E-02 | 1.34E-02 | -3.039 | 0.00237291 | 0.04402904 | protein_coding | LYPD1 | 116372 |
| 783 | ENSG00000227475 | 6.40 | 5.51E-02 | 1.82E-02 | 3.037 | 0.00238673 | 0.04417101 | lncRNA |  |  |
| 784 | ENSG00000178538 | 197.20 | 2.75E-02 | 9.05E-03 | 3.035 | 0.00240585 | 0.04443231 | protein_coding | CA8 | 767 |
| 785 | ENSG00000177483 | 30.57 | -3.14E-02 | 1.03E-02 | -3.033 | 0.00242448 | 0.04466449 | protein_coding | RBM44 | 375316 |
| 786 | ENSG00000180287 | 5047.20 | 3.02E-02 | 9.96E-03 | 3.031 | 0.0024339 | 0.04479705 | protein_coding | PLD5 | 200150 |
| 787 | ENSG00000180875 | 31.78 | -3.16E-02 | 1.04E-02 | -3.031 | 0.00243893 | 0.04484869 | protein_coding | GREM2 | 64388 |
| 788 | ENSG00000167588 | 41.48 | 4.02E-02 | 1.33E-02 | 3.029 | 0.00245553 | 0.04503075 | protein_coding | GPD1 | 2819 |
| 789 | ENSG00000128655 | 101.21 | -5.26E-02 | 1.74E-02 | -3.027 | 0.0024707 | 0.04522159 | protein_coding | PDE11A | 50940 |
| 790 | ENSG00000249695 | 15.43 | 4.04E-02 | 1.33E-02 | 3.027 | 0.00247267 | 0.04522159 | lncRNA | LOC574538 | 574538 |
| 791 | ENSG00000088899 | 208.39 | -2.74E-02 | 9.06E-03 | -3.025 | 0.00248676 | 0.04539685 | protein_coding | LZTS3 | 9762 |
| 792 | ENSG00000188933 | 23.55 | -5.90E-02 | 1.95E-02 | -3.021 | 0.00252057 | 0.0456809 | transcribed_unprocessed_pseudogene | USP32P1 | 162632 |
| 793 | ENSG00000269235 | 9.75 | -3.70E-02 | 1.22E-02 | -3.020 | 0.0025238 | 0.0456809 | lncRNA | ZNF350-AS1 | 101669766 |
| 794 | ENSG00000165164 | 40.21 | -3.26E-02 | 1.08E-02 | -3.019 | 0.00253991 | 0.04582754 | protein_coding | CFAP47 | 286464 |
| 795 | ENSG00000179869 | 193.05 | 2.57E-02 | 8.51E-03 | 3.017 | 0.00255558 | 0.0460279 | protein_coding | ABCA13 | 154664 |
| 796 | ENSG00000248528 | 5.32 | -5.33E-02 | 1.77E-02 | -3.016 | 0.0025651 | 0.046158 | lncRNA |  |  |
| 797 | ENSG00000260254 | 111.52 | -9.71E-02 | 3.22E-02 | -3.012 | 0.00259721 | 0.04665258 | lncRNA |  |  |
| 798 | ENSG00000250846 | 25.46 | 2.92E-02 | 9.71E-03 | 3.010 | 0.00261257 | 0.04688663 | lncRNA | EPHA5-AS1 | 100144602 |
| 799 | ENSG00000054690 | 3044.27 | 2.51E-02 | 8.35E-03 | 3.007 | 0.00263641 | 0.04712044 | protein_coding | PLEKHH1 | 57475 |
| 800 | ENSG00000166707 | 12.24 | -3.55E-02 | 1.18E-02 | -3.007 | 0.00263991 | 0.04712044 | protein_coding | ZCCHC18 | 644353 |
| 801 | ENSG00000267194 | 159.30 | 2.73E-02 | 9.08E-03 | 3.004 | 0.00266309 | 0.047314 | NA |  |  |
| 802 | ENSG00000197385 | 62.15 | 2.61E-02 | 8.67E-03 | 3.004 | 0.00266695 | 0.047314 | protein_coding | ZNF860 | 344787 |
| 803 | ENSG00000125166 | 3962.08 | 2.72E-02 | 9.06E-03 | 3.004 | 0.00266726 | 0.047314 | protein_coding | GOT2 | 2806 |
| 804 | ENSG00000155761 | 109.15 | -3.88E-02 | 1.29E-02 | -3.003 | 0.00267321 | 0.047314 | protein_coding | SPAG17 | 200162 |
| 805 | ENSG00000270163 | 172.28 | 2.54E-02 | 8.46E-03 | 3.003 | 0.00267394 | 0.047314 | NA |  |  |
| 806 | ENSG00000154429 | 1945.54 | 2.55E-02 | 8.49E-03 | 3.002 | 0.00267868 | 0.04734067 | protein_coding | CCSAP | 126731 |
| 807 | ENSG00000181856 | 53.13 | -2.89E-02 | 9.64E-03 | -3.002 | 0.00268015 | 0.04734067 | protein_coding | SLC2A4 | 6517 |
| 808 | ENSG00000170703 | 15.58 | 3.81E-02 | 1.27E-02 | 3.001 | 0.00269411 | 0.04750394 | protein_coding | TTLL6 | 284076 |
| 809 | ENSG00000100234 | 113778.50 | 2.64E-02 | 8.82E-03 | 2.998 | 0.00271959 | 0.04783587 | protein_coding | TIMP3 | 7078 |
| 810 | ENSG00000184845 | 71.24 | -3.51E-02 | 1.17E-02 | -2.997 | 0.00272959 | 0.04796184 | protein_coding | DRD1 | 1812 |
| 811 | ENSG00000234602 | 4.92 | 7.03E-02 | 2.35E-02 | 2.994 | 0.00275661 | 0.04820776 | protein_coding | MCIDAS | 345643 |
| 812 | ENSG00000197977 | 757.68 | 4.17E-02 | 1.39E-02 | 2.993 | 0.00276033 | 0.04820776 | protein_coding | ELOVL2 | 54898 |
| 813 | ENSG00000144771 | 22.39 | -7.33E-02 | 2.45E-02 | -2.992 | 0.00277042 | 0.04834198 | protein_coding | LRTM1 | 57408 |
| 814 | ENSG00000161958 | 33.81 | -3.45E-02 | 1.15E-02 | -2.992 | 0.00277362 | 0.04835604 | protein_coding | FGF11 | 2256 |
| 815 | ENSG00000271141 | 10.52 | -3.21E-02 | 1.07E-02 | -2.991 | 0.00278365 | 0.04848892 | lncRNA |  |  |
| 816 | ENSG00000233844 | 16.84 | -3.67E-02 | 1.23E-02 | -2.985 | 0.00283268 | 0.04908844 | NA |  |  |
| 817 | ENSG00000232599 | 8.33 | -4.69E-02 | 1.57E-02 | -2.985 | 0.00283719 | 0.04912052 | processed_pseudogene |  |  |
| 818 | ENSG00000171596 | 17.01 | -3.51E-02 | 1.17E-02 | -2.985 | 0.00283941 | 0.04912052 | protein_coding | NMUR1 | 10316 |
| 819 | ENSG00000256518 | 28.25 | 5.68E-02 | 1.90E-02 | 2.983 | 0.00285862 | 0.04934705 | unprocessed_pseudogene |  |  |
| 820 | ENSG00000152969 | 42.03 | -4.45E-02 | 1.49E-02 | -2.981 | 0.00286865 | 0.04940712 | protein_coding | JAKMIP1 | 152789 |
| 821 | ENSG00000179452 | 6.11 | -8.83E-02 | 2.96E-02 | -2.981 | 0.00287307 | 0.04940712 | lncRNA | LINC01699 | 100287948 |
| 822 | ENSG00000135298 | 101.90 | -3.50E-02 | 1.17E-02 | -2.981 | 0.00287338 | 0.04940712 | protein_coding | ADGRB3 | 577 |
